# Substrate selectivity and inhibition of the human lysyl hydroxylase JMJD7

**DOI:** 10.1002/pro.5162

**Published:** 2024-09-14

**Authors:** Nurgül Bilgin, Anthony Tumber, Siddhant Dhingra, Eidarus Salah, Aziza Al‐Salmy, Sandra Pinzón Martín, Yicheng Wang, Christopher J. Schofield, Jasmin Mecinović

**Affiliations:** ^1^ Department of Physics, Chemistry and Pharmacy University of Southern Denmark Odense Denmark; ^2^ Chemistry Research Laboratory, Department of Chemistry and the Ineos Oxford Institute for Antimicrobial Research University of Oxford Oxford UK

**Keywords:** biocatalysis, DRG1, enzymology, JMJD7 hydroxylase, lysine, oxidation

## Abstract

Jumonji‐C (JmjC) domain‐containing protein 7 (JMJD7) is a human Fe(II) and 2‐oxoglutarate dependent oxygenase that catalyzes stereospecific C3‐hydroxylation of lysyl‐residues in developmentally regulated GTP binding proteins 1 and 2 (DRG1/2). We report studies exploring a diverse set of lysine derivatives incorporated into the DRG1 peptides as potential human JMJD7 substrates and inhibitors. The results indicate that human JMJD7 has a relatively narrow substrate scope beyond lysine compared to some other JmjC hydroxylases and lysine‐modifying enzymes. The geometrically constrained (*E*)‐dehydrolysine is an efficient alternative to lysine for JMJD7‐catalyzed C3‐hydroxylation. γ‐Thialysine and γ‐azalysine undergo C3‐hydroxylation, followed by degradation to formylglycine. JMJD7 also catalyzes the S‐oxidation of DRG1‐derived peptides possessing methionine and homomethionine residues in place of lysine. Inhibition assays show that DRG1 variants possessing cysteine/selenocysteine instead of the lysine residue efficiently inhibit JMJD7 via cross‐linking. The overall results inform on the substrate selectivity and inhibition of human JMJD7, which will help enable the rational design of selective small‐molecule and peptidomimetic inhibitors of JMJD7.

## 1 INTRODUCTION

Non‐heme Fe(II) and 2‐oxoglutarate (2OG) dependent oxygenases catalyze a wide range of oxidative modifications on proteins and nucleic acids, most commonly involving hydroxylations and demethylations (Islam et al., 2018; Markolovic et al., 2015). The plethora of biological functions of 2OG oxygenases in animals include roles in lipid metabolism and carnitine biosynthesis (Loenarz & Schofield, 2008; Maas et al., 2020), DNA/RNA damage repair, and chromatin modifications that have important roles in transcriptional regulation (Greer & Shi, 2012; Leśniak et al., 2017; Loenarz & Schofield, 2011; Zhuang et al., 2015). The Jumonji‐C (JmjC) enzymes form a distinct structural subfamily of 2OG‐dependent oxygenases, some of which act as lysine‐residue hydroxylases and/or *N* ^ε^‐methyllysine demethylases (KDMs) (Johansson et al., 2014; Markolovic et al., 2016). Following the identification of factor inhibiting hypoxia inducible factor (FIH) as a JmjC asparagine‐residue hydroxylase (Hewitson et al., 2002), multiple JmjC KDMs have been identified; the latter catalyze the oxidative demethylation of *N* ^ε^‐methylated lysine and N‐methylated arginine residues on histone tails, initially forming a hemiaminal intermediate, which fragments to give the demethylated product and formaldehyde (Markolovic et al., 2015, 2016). Multiple human JmjC KDM subfamilies have been characterized and many JmjC KDMs are linked to diseases, including cancer and genetic disorders (Islam et al., 2018). Another set of structurally discrete JmjC subfamily members catalyze the oxidation of methylene groups in amino acid residue side chains to form stable hydroxylated residues (Del Rizzo et al., 2012; Hewitson et al., 2002; Lando et al., 2002). Three of these JmjC hydroxylases are reported to catalyze the oxidation of lysine‐residues in human proteins: JMJD4 catalyzes C4 Lys hydroxylation, JMJD6 catalyzes C5 Lys hydroxylation, and the subject of our studies, JMJD7 catalyzes C3 Lys hydroxylation (Feng et al., 2014; Markolovic et al., 2018; Webby et al., 2009).

**FIGURE 1:**
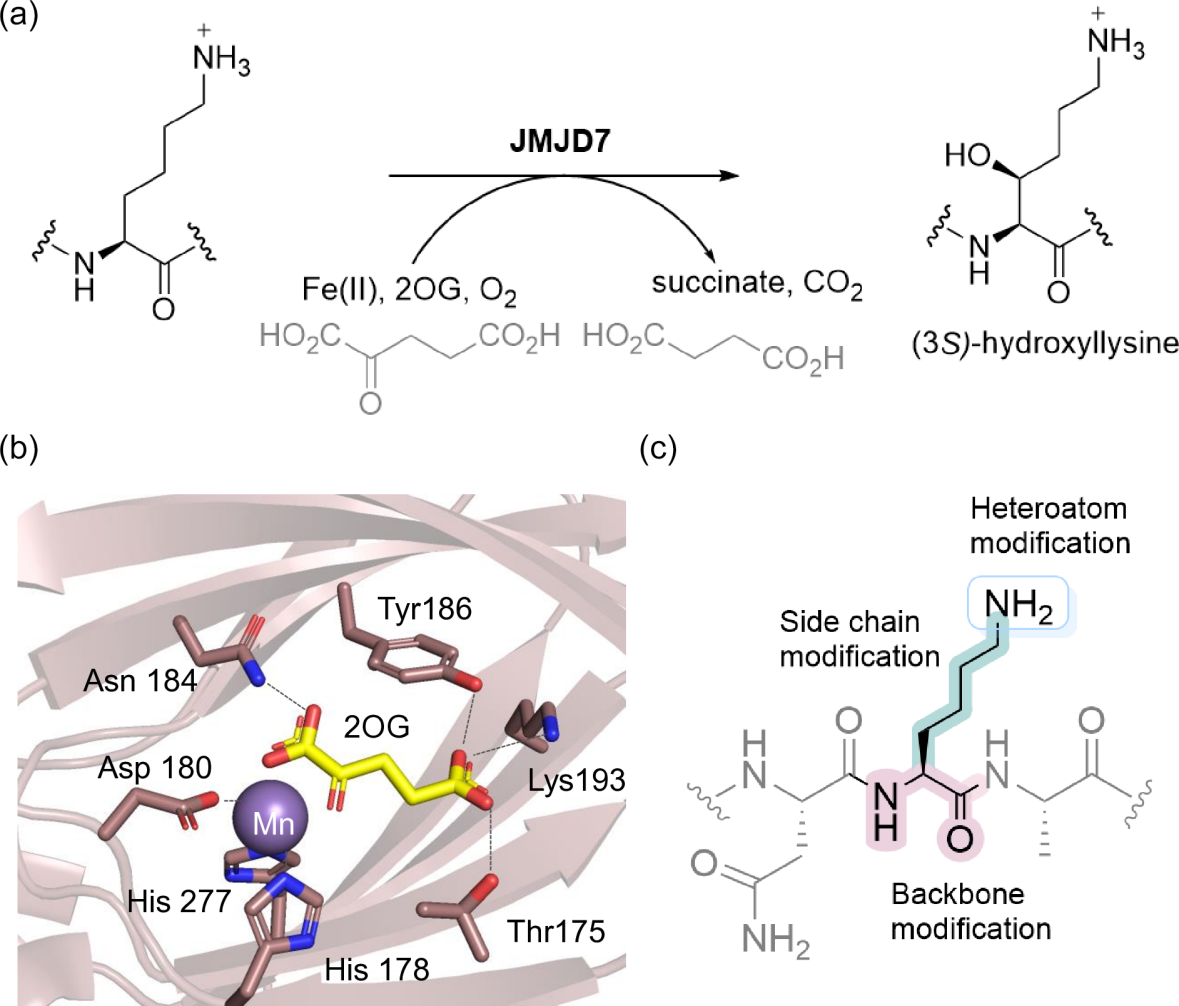
JMJD7‐catalyzed hydroxylation of Lys22 of DRG1. (a) Stereospecific C3‐lysine hydroxylation catalyzed by JMJD7, (b) View from a crystal structure of JMJD7 (pink) in complex with 2‐oxoglutarate (2OG, yellow) and Mn(II) (substituting for catalytically active Fe(II)) (purple) (PDB:5NFN) (Markolovic et al., 2018), (c) Rationale behind the synthesis of selected lysine analogs for screening as JMJD7 substrates and inhibitors.

Jumonji domain‐containing 7 (JMJD7) is a dimeric JmjC 2OG oxygenase that is structurally related to factor inhibiting hypoxia inducible factor (FIH) (Elkins et al., 2003; Hewitson et al., 2002), TYW5 (Kato et al., 2011), JMJD4‐6 (Del Rizzo et al., 2012; Feng et al., 2014; Webby et al., 2009), and the ribosomal oxygenases MYC‐induced nuclear antigen 53 (MINA53) and Nucleolar protein 66 (NO66) (Chowdhury et al., 2014), all of which are proposed to have biological roles in protein synthesis and/or cellular regulation. JMJD7 is a stereospecific (3*S*)‐lysyl hydroxylase, catalyzing the oxidation of two related members of the human translation factor (TRAFAC) GTPase family, i.e. developmentally regulated GTP‐binding proteins 1 and 2 (DRG1/2), though DRG2 is a less efficient substrate than DRG1 (Figure 1a) (Ishikawa et al., 2005; Markolovic et al., 2018). DRG1 and DRG2 are linked to cell growth in diverse cell types and species (de Krom et al., 2009; de Ligt et al., 2012; Ishikawa et al., 2003, 2005; Lu et al., 2016; Wei et al., 2004); in addition to animals, DRG‐like proteins have been identified in yeast and plants (Daugeron et al., 2011; Ishikawa et al., 2009; Nelson et al., 2009). Emerging evidence suggests that JMJD7 and DRG1/DRG2 have roles in diseases, including cancer and mental disorders (Ishikawa et al., 2005; Markolovic et al., 2018). Crystallographic analysis of JMJD7 reveals a characteristic dimer interface formed by α‐helices present in both N‐ and C‐terminal regions relative to the distorted double‐stranded β‐helix (DSBH) 2OG oxygenase core fold and a disulfide cross‐linkage formed between cysteines (Cys47) of each monomer (Chowdhury et al., 2022; Markolovic et al., 2018). The catalytic DSBH core comprises eight β‐strands, arranged as two antiparallel sheets, which are responsible for binding the 2OG cosubstrate and the Fe(II) cofactor, in the latter case via a HisXAsp. His triad of chelating residues (Figure 1b). A crystal structure of the JMJD7‐substrate complex is not yet available, though the hydroxylation of Lys22 residue of DRG1/2 appears to resemble that of the JMJD4‐catalyzed C4‐lysyl hydroxylation of eRF1, as in both cases the hydroxylated lysine is located in the N‐terminal region of conserved helix‐turn‐helix (HTH) motifs (Feng et al., 2014; Markolovic et al., 2018).

The available biomolecular evidence demonstrates that some JmjC hydroxylases are selective, both with respect to the residues that they oxidize and set of proteins that they accept as substrates, whereas others are much less selective. For example, MINA53 and NO66 appear to be selective, whereas FIH and JMJD6 are more promiscuous (Choi et al., 2020; Islam et al., 2018; Türkmen et al., 2023). Factors influencing the selectivity of JmjC hydroxylases include the active site architecture, substrate sequence, and overall substrate fold, making it difficult to predict the selectivity of individual enzymes such as JMJD7.

Here, we report studies on the substrate selectivity and inhibition of human JMJD7 employing peptide fragments of its preferred DRG1 substrate, substituting the DRG1‐substrate Lys22 with a diverse panel of natural and unnatural residues (Figure 1c). The overall results inform on the selectivity of JMJD7 and will help in the rational design of selective small‐molecule or peptidomimetic inhibitors of JMJD7. They also highlight the possibility of using the JmjC hydroxylases for biocatalytic post‐translational modification of proteins.

## 2 RESULTS

### 2.1 The panel of lysine analogs of DRG1 peptides

To investigate whether human JMJD7 exhibits activity on DRG1 peptides possessing diverse lysine derivatives at residue 22, we synthesized a set of 25‐mer fragment peptides of DRG1 (residues 16–40) (Figure 2). Natural and unnatural amino acids were incorporated at position 22 (Figure 2), as follows: (i) epimeric D‐lysine, (ii) shorter and longer “Lys” chain lengths, (iii) modification or substitution of the N^ε^‐amino group, (iv) side chain modifications, and (v) main chain modifications. The DRG1 peptides were synthesized employing Fmoc‐based solid‐phase peptide synthesis (SPPS), and then purified by preparative RP‐HPLC; their purity was assessed by MALDI‐TOF MS and analytical HPLC (Figures S1–S30, Table S1).

**FIGURE 2:**
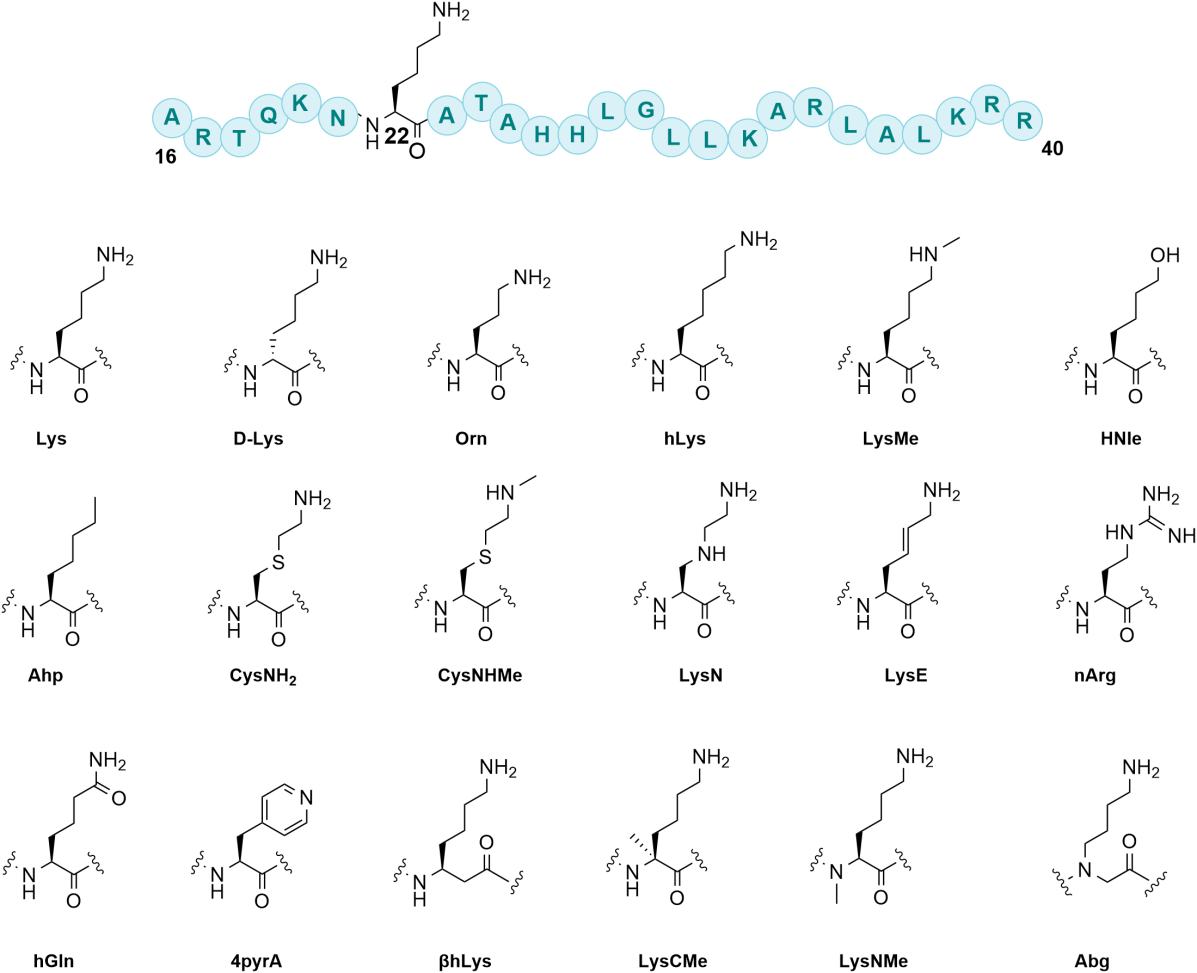
Lysine residue substrate analogs incorporated into 25‐mer DRG1 peptide fragments at position 22.

### 2.2 JMJD7‐catalyzed hydroxylation of DRG1 lysine analogs

**FIGURE 3:**
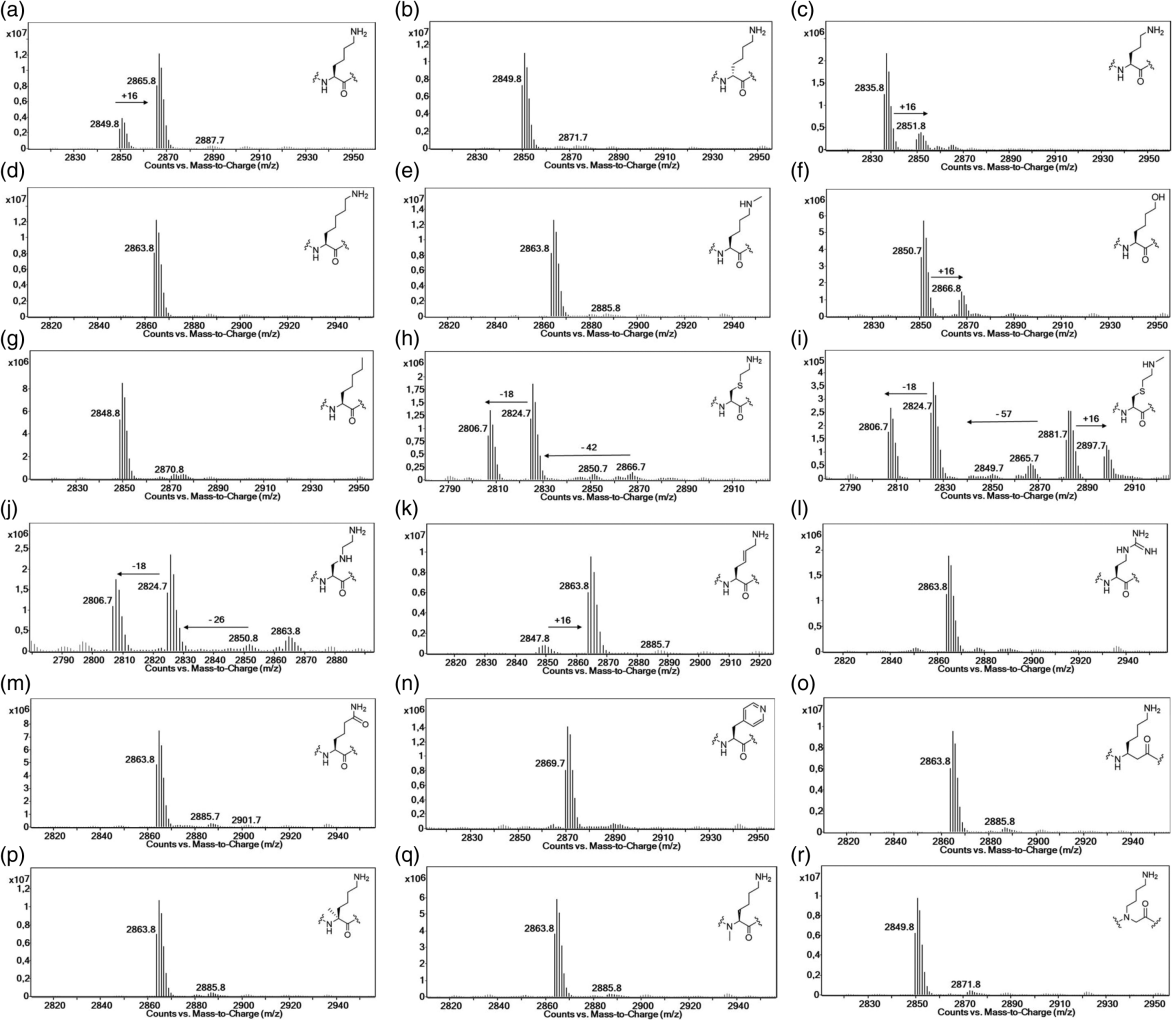
LC–MS data showing potential for JMJD7‐catalyzed (2 μM) hydroxylation of DRG1 peptides (10 μM) in the presence of 2OG (10 μM), FAS (10 μM) and LAA (100 μM). (a) DRG1‐Lys, (b) DRG1‐D‐Lys, (c) DRG1‐Orn, (d) DRG1‐hLys, (e) DRG1‐LysMe, (f) DRG1‐HNle, (g) DRG1‐Ahp, (h) DRG1‐CysNH_2_, (i) DRG1‐CysNHMe, (j) DRG1‐LysN, (k) DRG1‐LysE, (l) DRG1‐nArg, (m) DRG1‐hGln, (n) DRG1‐4pyrA, (o) DRG1‐βhLys, (p) DRG1‐LysCMe, (q) DRG1‐LysNMe, and (r) DRG1‐Abg. See Figure 4 for description of the fragmented products resulting from reaction of DRG1‐CysNH_2_ and DRG1‐LysN.

Initial assays were carried out by incubation of purified recombinant (Markolovic et al., 2018) human JMJD7 (2 μM) in the presence of a DRG1 derived peptide (10 μM), 2OG (10 μM), ferrous ammonium sulfate (FAS, 10 μM), and L‐ascorbate (LAA, 100 μM) in Tris buffer at pH 7.5 for 2 h at room temperature. Potential conversion to a hydroxylated product (+16 Da mass shift) was monitored by liquid‐chromatography‐mass‐spectrometry (LC–MS). Under these standard conditions, the extent of hydroxylation of the DRG1‐Lys substrate by JMJD7 was ~78% (Figure 3a), but the DRG1‐Lys substrate epimer DRG1‐D‐Lys was not hydroxylated within detection limits (Figure 3b). The assay results also implied that DRG1‐Orn was hydroxylated, but to a lower extent (~21%) than DRG1‐Lys. DRG1‐hLys did not undergo hydroxylation within detection limits, indicating that lysine possesses an optimal side chain length for productive binding and catalysis at the JMJD7 active site (Figure 3c,d). DRG1 peptides bearing LysMe and Ahp residues were not JMJD7 substrates (Figure 3e,g). The alcohol‐containing peptide hydroxynorleucine (DRG1‐HNle), was hydroxylated at relatively low levels (~24%), implying that H‐bonding interactions and positive charge of the N^ɛ^‐amino group of lysine are important for efficient binding and catalysis, but that a neutral alcohol can still be accommodated at the active site (Figure 3f) (Markolovic et al., 2018).

**FIGURE 4:**
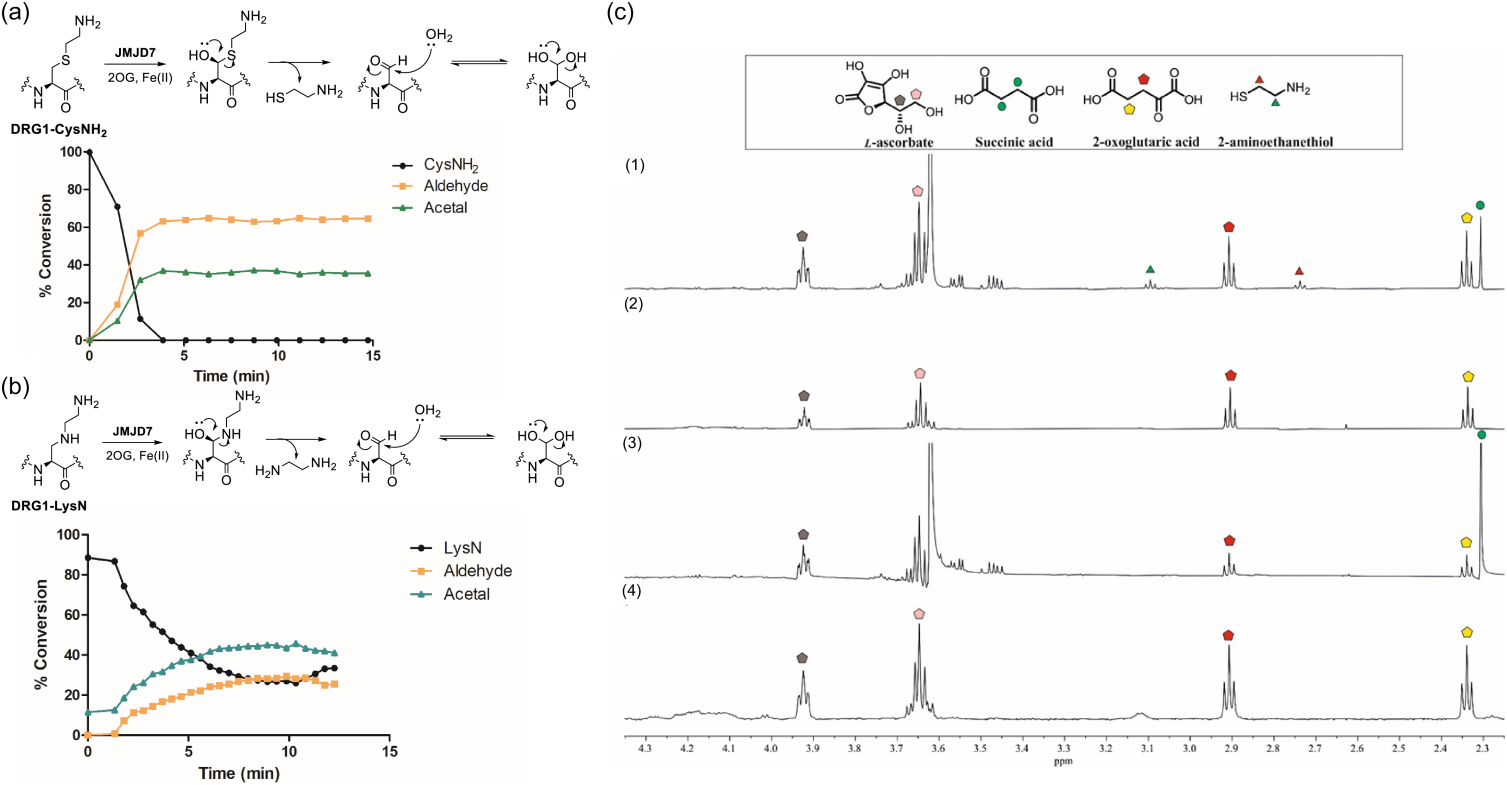
JMJD7‐catalyzed hydroxylation of DRG1 peptides possessing γ‐thialysine (DRG1‐CysNH_2_) and γ‐azalysine (DRG1‐LysN). (a) SPE‐MS assays showing time‐course of the DRG1‐CysNH_2_ (1.0 μM) in the presence of JMJD7 (50 nM), following the fragmentation of a hemithioacetal intermediate to cysteamine and the formylglycine DRG1 peptide that is in equilibrium with its hydrated acetal form, (b) SPE‐MS assays showing time‐course of DRG1‐LysN (1.0 μM) in the presence of JMJD7 (50 nM), following the fragmentation of a hemiacetal intermediate to ethylenediamine and the formylglycine peptide that is in equilibrium with its hydrated acetal form, and (c) NMR studies of DRG1‐CysNH_2_ (200 μM) (1; JMJD7‐catalyzed hydroxylation (10 μM), *t* = 15 min), 2; no‐enzyme control) in comparison with DRG1‐Lys substrate (200 μM) (3; JMJD7‐catalyzed hydroxylation (10 μM), *t* = 15 min), 4; no‐enzyme control).

Notably, the JMJD7‐catalyzed hydroxylation of the DRG1‐CysNH_2_, DRG1‐CysNHMe and DRG1‐LysN peptides provides evidence that hydroxylation proceeds via initial C3‐hydroxylation to form unstable hemithioacetal/hemiaminal intermediates, which undergo subsequent (likely) non‐enzymatic fragmentation to form DRG1‐formylglycine and its hydrated products (Figure 3h–j, Figure 4, Figure S31). Note that JMJD7 displays a lower level of activity for the DRG1‐CysNHMe compared to the DRG1‐CysNH_2_ and DRG1‐LysN derivatives, as observed by MS detection of a possibly stable hemithioacetal product (12%, Figure 3i). These observations demonstrate the potential of γ‐thialysine and γ‐azalysine‐containing peptides to compete with the lysine‐containing DRG1 peptide.

The geometrically constrained DRG1‐LysE peptide appeared to be efficiently hydroxylated by JMJD7, yielding 90% conversion, likely at C3, though this is uncertain as there is a possibility for allylic radical intermediate rearrangement to give a C5 hydroxylated product (Islam et al., 2018) (Figure 3k), in accord with similar observations reported for KMT‐ or KAT‐catalyzed modifications of (*E*)‐dehydrolysine in histone peptides (Al Temimi, White, et al., 2020; Proietti et al., 2021). None of the other tested DRG1 peptides with altered side chains and main chains were hydroxylated by JMJD7 under our standard assay conditions. Overall, the results indicate that the substrate lysine likely adopts an extended conformation, and that the positive charge of *N* ^ε^‐lysine, the lysine side chain length and stereochemistry are important for substrate binding at the JMJD7 active site; however, these are not essential for productive binding and JMJD7 catalysis, as also observed for some other lysine‐modifying 2OG oxygenases and related enzymes (Al Temimi, Amatdjais‐Groenen, et al., 2019; Al Temimi, Teeuwen, et al., 2019; Belle et al., 2017; Choi et al., 2020; Leissing et al., 2022; Tarhonskaya et al., 2015).

To further investigate the catalytic efficiency of JMJD7, assays with a higher JMJD7 concentration (5 μM) were carried out. Similar levels of hydroxylation were observed as observed for DRG1 substrates under the standard conditions, with no observed clear evidence for hydroxylation of those DRG1 peptides not previously observed to be substrates (Figure 3, Figure S32). With a higher 2OG concentration (2 μM JMJD7, 20 μM 2OG, 10 μM FAS, 100 μM LAA, pH 7.5) than used with the standard conditions, hydroxylation of the DRG1‐Lys substrate proceeds efficiently, reaching ~98% after 2 h incubation (Figure S32). Low levels of potential hydroxylation were detected for DRG1 containing D‐Lys, hLys and LysMe analogs (~6%, ~5%, and ~ 7%, Figure S33), though we cannot rule out the observed oxidation results from partial epimerization during synthesis of the D‐Lys substrate to yield the DRG1‐Lys substrate. Notably, the DRG1‐Orn and DRG1‐HNle peptides underwent ~31% and ~56% hydroxylation, respectively (Figure S32). By contrast, the DRG1 peptide bearing analogs Ahp, nArg, hGln and 4pyrA did not manifest evidence for hydroxylation even under the optimized conditions (Figure 3g,l–n, Figure S32). As shown in our initial assays, the peptides with backbone alterations, as in DRG1‐βhLys, DRG1‐LysCMe, DRG1‐LysNMe and DRG1‐Abg, were not hydroxylated within our detection limits, demonstrating that the backbone plays a role in optimal positioning of the lysine side chain in the JMJD7 active site (Figure 3o–r, Figure S33).

### 2.3 JMJD7‐catalyzed fragmentation studies of DRG1‐CysNH_2_ and DRG1‐LysN

**FIGURE 5:**
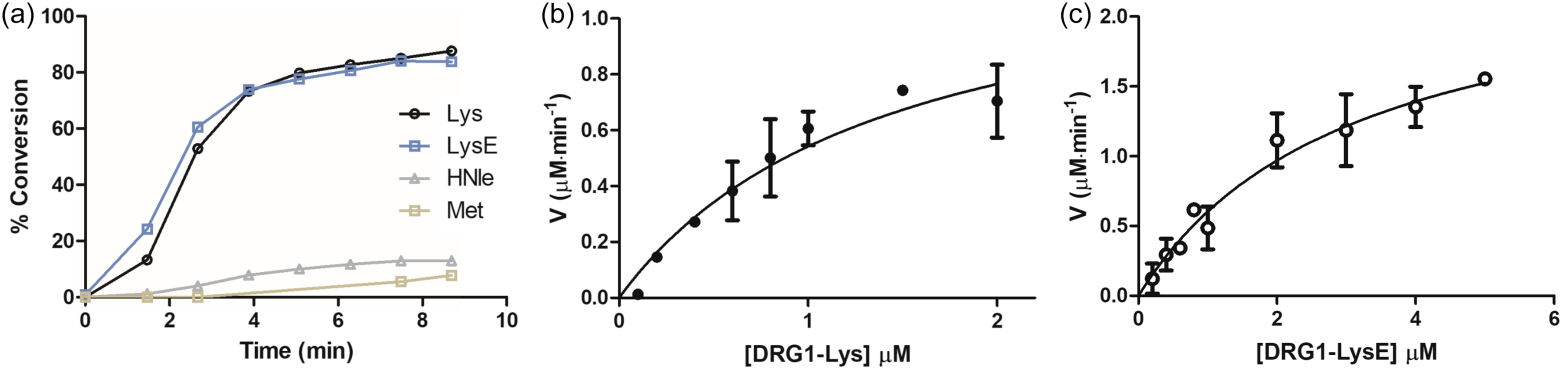
Time‐course and steady‐state kinetics or the JMJD7‐catalyzed hydroxylation of DRG1 peptides using SPE‐MS assays. (a) Time‐course data of the JMJD7‐catalyzed (50 nM) hydroxylation of DRG1‐Lys (1.0 μM), DRG1‐LysE (1.0 μM), DRG1‐HNle (1.0 μM), and DRG1‐Met (1.0 μM), (b) Determination of the kinetic parameters for the DRG1‐Lys substrate, and (c) Determination of kinetic parameters for the DRG1‐LysE substrate analog.

Having shown that introduction of γ‐thialysine and γ‐azalysine residues into DRG1 peptides generates substrates that are efficiently hydroxylated by JMJD7, giving nascent products that fragment to give formylglycine, we explored whether these two peptides could compete with the DRG1‐Lys substrate for JMJD7 catalysis. The reactions of DRG1‐Lys, DRG1‐CysNH_2_ and DRG1‐LysN (1.0 μM) by JMJD7 (50 nM) were investigated as a function of time using SPE‐MS assays. The results revealed that JMJD7‐catalyzed hydroxylation of DRG1‐CysNH_2_ is complete within 4 min, yielding the corresponding DRG1‐formylglycine in an apparent equilibrium with its hydrated acetal form (Figure 4a). DRG1‐LysN appeared to be oxidized more slowly than DRG1‐CysNH_2_ (Figure 4b), whereas DRG1‐Lys manifested ~90% hydroxylation within 9 min under the same conditions (Figure 5a). The results indicate that the selectivity of JMJD7 for lysine and its analogs bearing a heteroatom at the γ/C3 position follows the trend: S > C > N (DRG1‐CysNH_2_ > DRG1‐Lys > DRG1‐LysN) and that the introduction of γ‐thialysine increases the reaction rate of JMJD7‐catalyzed hydroxylation (Figure S33).

To validate the fragmentation of the product on hydroxylation of the DRG1‐CysNH_2_ peptide, we performed NMR studies focusing on the appearance of small molecules, comparing the results with the DRG1‐Lys substrate. Reactions were carried out by incubating the DRG1 peptide (200 μM) in the presence or absence of enzyme (10 μM JMJD7, 30 μM FAS, 200 μM 2OG, 500 μM LAA, phosphate buffer in 10% D_2_O v/v, pH 7.4). Consistent with LC–MS data, the NMR assays revealed that in each case, JMJD7‐catalyzed hydroxylation of DRG1‐Lys (spectrum 3) and DRG1‐CysNH_2_ (spectrum 1) is tightly coupled to oxidation of 2OG to succinate, as typical for Fe(II)/2OG‐dependent oxygenases (Flashman et al., 2010; McNeill et al., 2005; Wilkins et al., 2018) (Figure 4c, Figures S34 and S35). The observation of a DRG1‐CysNH_2_ derived product with two methylene groups (triplets, δ ~ 3.13 ppm, δ ~ 2.75 ppm) together with spiking using a standard solution confirmed the formation of cysteamine (Figure 4c (1), Figure S35a). Comparisons with no‐enzyme controls with the DRG1‐Lys and DRG1‐CysNH_2_ peptides showed a lack of fragmentation and succinate formation in the absence of JMJD7 (Figure 4c, spectra 2 and 4), in line with controls in LC–MS assays (Figure S37). Time‐course NMR assays revealed that the cysteamine fragment is formed within 15 min in the case of JMJD7‐catalyzed DRG1‐CysNH_2_ oxidation. A loss in intensity of the cysteamine peaks was observed over time, possibly indicating oxidation and/or interactions with JMJD7 (Figure S35).

### 2.4 Enzyme kinetic studies

To quantify the relative efficiencies of DRG1‐Lys and the substrate analog DRG1‐LysE, we carried out kinetics analyses using SPE‐MS assays as the levels of JMJD7‐catalyzed hydroxylation were sufficient for these two substrates. DRG1‐HNle was not evaluated since its turnover was low (Figure 5a). The results revealed that the DRG1‐LysE analog is a comparable substrate to DRG1‐Lys, as shown by similar *k*_cat_/*K*_m_ values (8.0 μM^−1^ min^−1^ and 9.3 μM^−1^ min^−1^, respectively) (Figure 5b,c). The small differences in *k*_cat_ and *K*_m_ values for DRG1‐Lys (*k*_cat_ 13.0 ± 0.36, *K*_m_ 1.4 ± 0.59) and DRG1‐LysE (*k*_cat_ 24.7 ± 0.36, *K*_m_ 3.1 ± 0.81), suggest that flexibility of the lysine side chain may be important for efficient JMJD7 binding and catalysis. It should be noted that substrate inhibition of JMJD7 was observed in the case of increased concentrations of the DRG1‐Lys substrate, as observed for some other 2OG oxygenases (Tarhonskaya et al., 2015), implying that DRG1 may be an efficient substrate within a narrow concentration range (Figure S38).

### 2.5 JMJD7‐catalyzed oxidation of DRG1 methionine analogs

**FIGURE 6:**
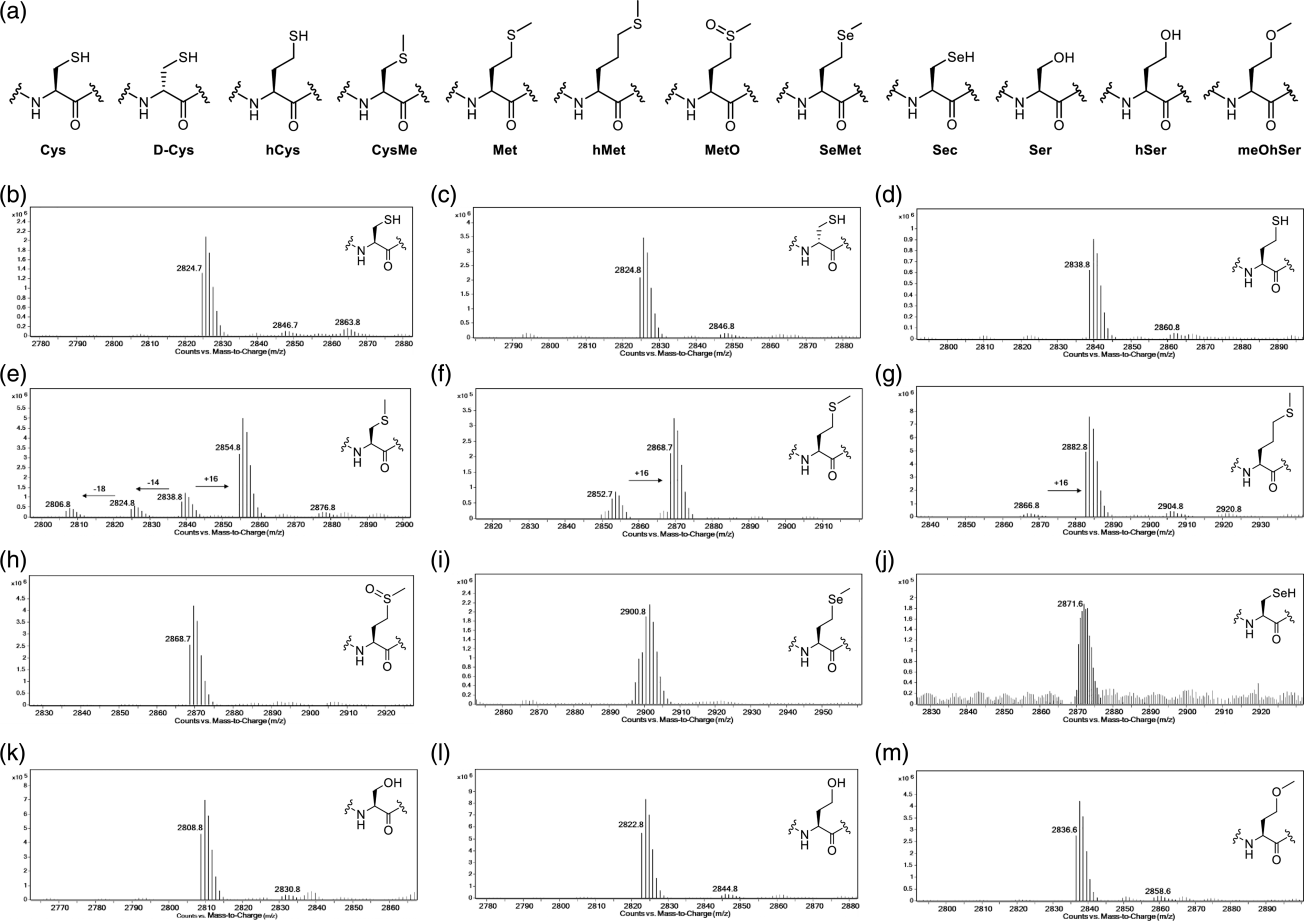
Cysteine and methionine analogs as JMJD7 substrates. (a) Structures of cysteine/methionine analogs. LC–MS data showing potential for JMJD7‐catalyzed (2 μM) hydroxylation of DRG1 peptides (10 μM) in the presence of 2OG (10 μM), FAS (10 μM) and LAA (100 μM). (b) DRG1‐Cys, (c) DRG1‐D‐Cys, (d) DRG1‐hCys, (e) DRG1‐CysMe, (f) DRG1‐Met, (g) DRG1‐hMet, (h) DRG1‐MetO, (i) DRG1‐SeMet, (j) DRG1‐Sec, (k) DRG1‐Ser, (l) DRG1‐hSer, and (m) DRG1‐meOhSer.

Having found that the cysteine‐based lysine analog peptides (DRG1‐CysNH_2_ and DRG1‐CysNHMe) undergo fragmentation following oxidation by JMJD7, we proceeded to investigate cysteine and methionine analogs (D‐Cys, hCys, CysMe, Met) as JMJD7 substrates and inhibitors by incorporating these amino acids into DRG1 peptide fragments at position 22 (Figure 6). LC–MS assays reveal that the DRG1 peptides bearing Cys, D‐Cys and hCys were not JMJD7 substrates (Figure 6b–d). The DRG1‐CysMe peptide, however, was oxidized relatively efficiently by JMJD7 as manifested by a + 16 Da mass shift, with possible fragmentation to give formylglycine and the hydrated acetal product, but to lesser extent than observed for DRG1‐CysNH_2_ (Figure 6e). Interestingly, JMJD7‐catalyzed hydroxylation of DRG1‐Met and DRG1‐hMet yielded high degree of oxidation (~86% and ~89%, respectively), without apparent fragmentation (Figure 6f,g). This observation suggests that DRG1‐Met and DRG1‐hMet are oxidized to methionine sulfoxide and homomethionine sulfoxide, respectively. Kinetics data were not obtained in case of DRG1‐Met, as the turnover appeared to be low (Figure 5a). Changing the sulfur of methionine to selenium or oxygen, as in DRG1‐SeMet and DRG1‐meOhSer, or to sulfoxide (DRG1‐MetO) showed no evidence for oxidation (Figure 6h,i,m). The lack of activity with DRG1‐SeMet compared to DRG1‐Met is interesting given DRG1‐SeMet may be expected to be intrinsically more prone to oxidation than DRG1‐Met. Furthermore, DRG1‐Sec, DRG1‐Ser, DRG1‐hSer and DRG1‐meOhSer did also not undergo oxidation in the presence of JMJD7 (Figure 6j–m).

To validate the selectivity of the Met oxidation reaction, we performed chemoselective oxidation reactions using Selectfluor (Emenike et al., 2024). DRG1‐Ser and DRG1‐MetO peptides were used as negative controls, both remaining unmodified in the presence of Selectfluor (10 eq.) and pyridine (5 eq.) (Figure S39a,b). Conversely, the DRG1‐Met peptide underwent oxidation to methionine sulfoxide as manifested by a + 16 Da shift (Figure S39c). The oxidation by Selectfluor of JMJD7‐catalyzed reaction of the DRG1‐Met peptide did not manifest any further oxidation (Figure S39d,e). Notably, these results suggest that JMJD7 catalyzes S‐oxidation of Met22 on the DRG1 peptide fragment. Moreover, DRG1‐hMet also undergoes S‐oxidation to a high degree (without fragmentation) (Figure 6g, Figure S40).

### 2.6 Inhibition studies

**FIGURE 7:**
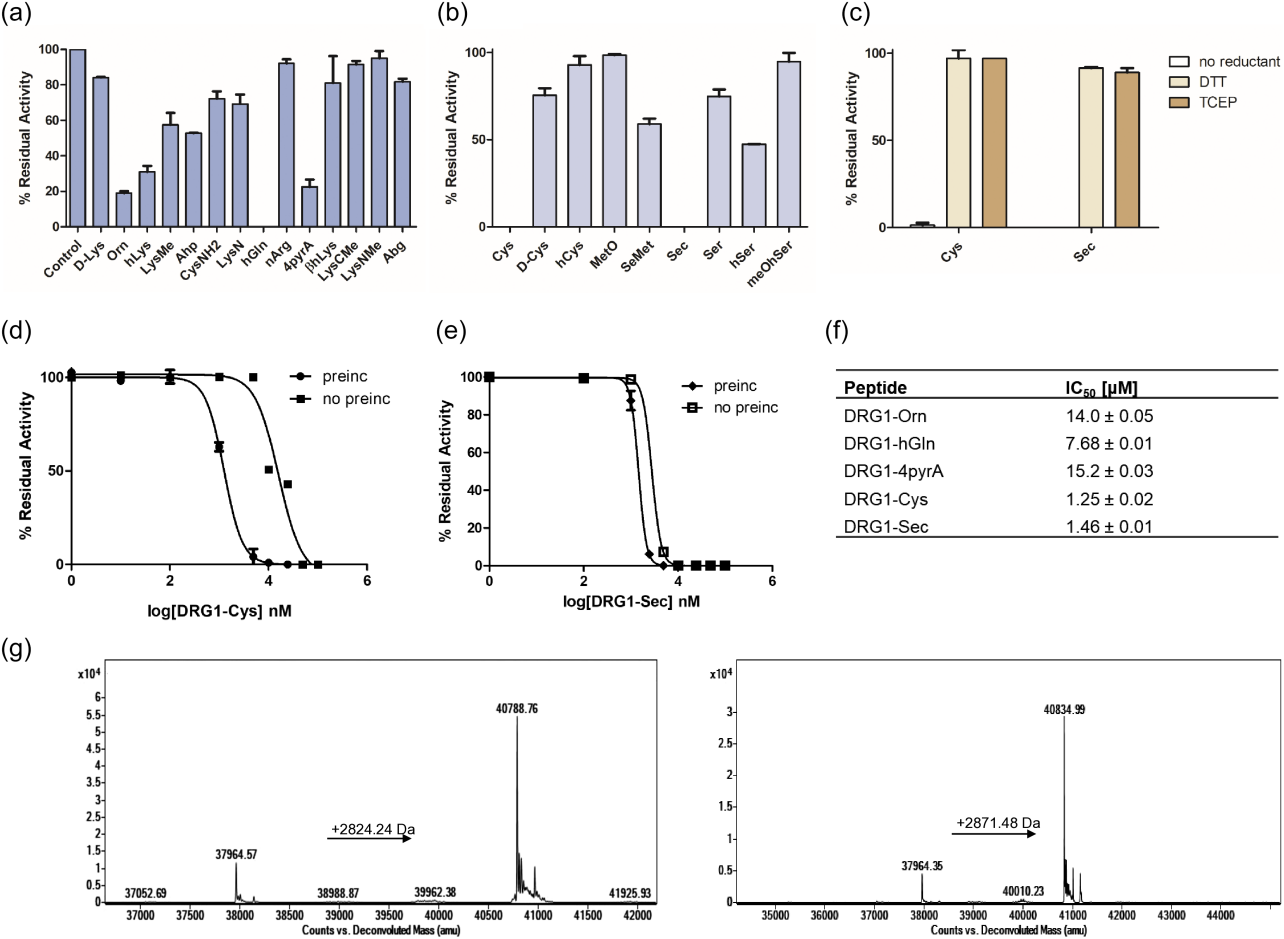
Inhibition assays of JMJD7‐catalyzed hydroxylation of DRG1‐Lys. (a) Single point inhibition data of JMJD7 (200 nM) by DRG1 lysine analog peptides (50 μM) in the presence of the DRG1‐Lys substrate (5 μM), (b) Single point inhibition data of JMJD7 (200 nM) by DRG1 cysteine analog peptides (50 μM) in the presence of DRG1‐Lys substrate (5 μM), (c) Single point inhibition data of JMJD7 (200 nM) by DRG1‐Cys or DRG1‐Sec peptides (10 μM) in the presence of DRG1‐Lys substrate (5 μM), in the presence and absence of a reductant in reaction buffer (DTT or TCEP), (d) Dose–response curves showing inhibition of JMJD7 in the presence of the DRG1‐Cys, with or without 15 min preincubation, (e) Dose–response curves showing JMJD7 inhibition by DRG1‐Sec, with or without 15 min preincubation, (f) IC_50_ values for DRG1 peptides as JMJD7 inhibitors. Error bars reported as SE, carried out in duplicate, and (g) Q‐TOF‐MS data of intact JMJD7 (2 μM) after 15 min incubation with DRG1‐Cys (20 μM, left) and DRG1‐Sec (20 μM, right). The observed mass shifts correspond to S‐S and S‐Se cross‐linking: JMJD7‐DRG1‐Cys, observed: 40.79 kDa; JMJD7‐DRG1‐Sec, observed: 40.84 kDa.

Many of the DRG1 peptides possessing lysine/cysteine analogs from our panel were not JMJD7 substrates, but since they may still bind at the active site, we investigated whether they are JMJD7 inhibitors by employing MALDI‐TOF MS assays. Due to signal overlaps between some DRG1 peptides and the reference DRG1‐Lys substrate, we synthesized a shorter reference substrate peptide (DRG1‐Lys, residues 17–40) for use in the MS‐based inhibition assays. Synthetic DRG1 peptides (residues 16–40, 10 or 50 μM) were preincubated with JMJD7 (200 nM) for 15 min, after which time the DRG1‐Lys substrate (residues 17–40, 5 μM), 2OG (10 μM), FAS (20 μM) and LAA (200 μM) were added, and the reaction were carried out for 25 min at room temperature. Most of the DRG1 peptides were not active as JMJD7 inhibitors at 10 μM (residual activity of JMJD7 >50%) (Figure S41a,b), with exceptions including the DRG1‐Orn, DRG1‐hGln and DRG1‐4pyrA peptides. The half‐maximum inhibitory concentrations (IC_50_) values were determined for peptides that showed clear inhibition in the presence of 50 μM DRG1 analogs, i.e., the Orn, hGln and 4pyrA peptides (Figure 7a,f). DRG1‐Orn was found to have an IC_50_ of 14.0 μM, suggesting that shortening the side chain can be beneficial for inhibition (Figure S42); note, however, that DRG1‐Orn is apparently a poor JMJD7 substrate (Figure 3). DRG1‐hGln manifested a slightly increased inhibition (IC_50_ 7.68 μM), while the more bulky side chain containing DRG1‐4pyrA was also an inhibitor (IC_50_ 15.2 μM) (Figure S42).

Interestingly, of the cysteine‐based analogs, the most efficient JMJD7 inhibition was observed for DRG1‐Cys (IC_50_ 1.25 μM) and DRG1‐Sec (IC_50_ 1.46 μM); for all the other tested cysteine analogs, no substantial inhibition was detected (Figure 7b). We considered that the potent inhibition with DRG1‐Cys/Sec peptides may involve covalent reaction with the active site proximate cysteine residue (Cys132) of JMJD7 (Figure 7b–e, Figure S43) (Brewitz et al., 2020; Markolovic et al., 2018; Sekirnik et al., 2009; Sun et al., 2021; Thun‐Hohenstein et al., 2022). We investigated this possibility by performing single point inhibition assays at 10 and 50 μM of DRG1‐Cys/Sec peptides in the presence of dithiothreitol (DTT) or tris(2‐carboxyethyl)phosphine (TCEP) (1 mM) as a reductant in the reaction buffer. The results show that both peptides lack inhibitory activity under the reducing conditions (Figure 7c, Figure S41c). The DRG1‐Cys and DRG1‐Sec inhibitory profiles against JMJD7 were also evaluated under same assay conditions but without preincubation, which resulted in increased IC_50_ values of 16.0 μM and 2.8 μM, respectively. These results indicate that with DRG1‐Cys preincubation is important to achieve substantial inhibition (Figure 7d,e). The more potent inhibition of JMJD7 by DRG1‐Sec, however, was sustained without preincubation. No inhibition of JMJD7 was observed with a shorter peptide fragment DRG1‐Cys (residues 19–25) under standard conditions, possibly reflecting a need for additional residues of DRG1 for productive JMJD7 binding.

To investigate inhibition by the DRG1‐Cys/Sec peptides, we performed protein‐observed analysis of JMJD7 (2 μM) in the presence of the DRG1‐Cys/Sec peptides (20 μM) under likely non‐denaturing conditions (Figure 7g). The observed masses correspond to the JMJD7‐DRG1‐Cys (40.79 kDa) and JMJD7‐DRG1‐Sec (40.84 kDa) adducts, implying that DRG1‐Cys/Sec might inhibit JMJD7 via formation of S‐S/Se‐S bonds, plausibly with Cys132, though further work is required to validate this (Figure 7g). Notably, by contrast with results for DRG1‐Met and DRG1‐SeMet, where the former was the better substrate, DRG1‐Sec resulted in a higher degree of adduct formation with JMJD7, possibly reflecting the higher intrinsic reactivity of selenocysteine compared to cysteine. QTOF‐MS results show that the adduct formation of JMJD7‐DRG1‐Sec occurs within 1 min (Figure S44b) and that the JMJD7‐DRG1‐Cys adduct formation was observed in lower extent at 1 min (Figure S44a), whereas no cross‐linking is observed in the presence of DTT in the buffer (Figure S44e,f). Adduct formation was not observed in case of the DRG1‐Ser analog with JMJD7, consistent with the proposal that the reaction proceeds via oxidative S‐S/Se‐S bond formation (Figure S45). Notably, consistent with MS observed evidence for cross‐linking, with sufficient reaction time DRG1‐Sec outcompetes DRG1‐Lys for binding to JMJD7 even when DRG1‐Lys is preincubated in excess (Figure S46). Taken together, these results indicate that DRG1‐Cys and DRG1‐Sec inhibit JMJD7 via a cross‐linking mechanism.

## 3 DISCUSSION

Numerous lysine residues in proteins are subject to posttranslational modifications with diverse biological consequences (Walsh et al., 2005). To investigate the substrate selectivity and inhibition of enzymes catalyzing post‐translational modifications, it is useful to explore a broad chemical space of substrate mimics (Islam et al., 2018). In this regard unnatural residues can act as chemical tools for understanding lysine‐modifying enzymes (Maas et al., 2022; Nadal et al., 2018; Wright et al., 2016), in particular 2OG oxygenases, which have the capacity to catalyze diverse and unexpected reactions (for JmjC demethylases / hydroxylases, see e.g (Choi et al., 2020; Hopkinson et al., 2013; Walport et al., 2016).). Following pioneering studies on the collagen prolyl‐ and lysyl‐hydroxylases, the substrate selectivity of some human 2OG oxygenases catalyzing post‐translational hydroxylations has more recently been studied. Although there are discrepancies in the literature, the available evidence is that some 2OG dependent protein hydroxylases are highly selective (at least in isolated form), e.g., the hypoxia inducible factor prolyl hydroxylases (Cockman et al., 2019) and the ribosomal histidine residue hydroxylases MINA53 and NO66 (Türkmen et al., 2023). By contrast FIH, JMJD6, and AspH are more promiscuous in terms of the range of substrates that interact with and at least in the case of FIH in terms of the reaction types catalyzed (Brewitz et al., 2022).

The results reported here, employing a structurally and chemically diverse panel of lysine analogs as substrates and inhibitors for human lysyl C3‐hydroxylase JMJD7, imply that it has a relatively narrow substrate residue selectivity, though can accept certain lysine analogs. The LC–MS assays results of the JMJD7‐catalyzed hydroxylation of DRG1 peptides bearing lysine mimics were carried out under different assay conditions and reveal that substrate oxidation requires H‐bonding/ionic interactions of N^ɛ^ of lysine, as evidenced by a lack of activity with many of the analogs under standard conditions (e.g., DRG1‐Ahp and DRG1‐LysMe).

Most of the DRG1 analogs are poor inhibitors, with exceptions being the Orn‐, hGln‐, 4pyrA‐, Cys‐ and Sec‐containing DRG1 peptides that efficiently inhibit human JMJD7. DRG1‐Cys and DRG1‐Sec may inhibit, at least in part via a S‐S/Se‐S cross‐linking mechanism, and their inhibition potency drops under reducing conditions. It is notable that DRG1‐Sec cross‐links to JMJD7 more efficiently than does DRG1‐Cys (Figure S44). In this regard, although structures of JMJD7‐substrate complexes are not yet available, it is notable that JMJD7 contains a cysteine residue (Cys132) close to its active site (Figure S43) (Markolovic et al., 2018) that is likely a candidate for cross‐linking with DRG1‐Cys and DRG1‐Sec. Reaction with Cys132 could be exploited in the future development of JMJD7 selective covalently reacting inhibitors.

γ‐Thialysine has been shown to act as an effective lysine mimic for histone lysine methylation and acetylation by histone methyltransferases (KMTs) (Al Temimi, van der Wekken‐de, et al., 2019) and acetyltransferases (KATs) (Proietti et al., 2020); further *N* ^ε^‐methylated γ‐thialysine is recognized by epigenetic reader domains to a similar extent as natural *N* ^ε^‐methylated lysine residues (Hintzen et al., 2020), while γ‐azalysine is a good substrate for KMTs and KATs (Al Temimi, Merx, et al., 2020; Proietti et al., 2021). Interestingly, the results presented here demonstrate that DRG1 peptides possessing γ‐thialysine (DRG1‐CysNH2), γ‐methylthialysine (DRG1‐CysNHMe) and γ‐azalysine (DRG1‐LysN) are JMJD7 substrates likely forming unstable C3 hemithioacetal/hemiaminal intermediates that fragment to give cysteamine/ethylenediamine and a formylglycine residue (Figure 4 and Figures S33–S36). The finding that γ‐thialysine containing DRG1 peptides are efficient JMJD7 substrates, in some cases (DRG1‐CysNH_2_) being better substrates than the wildtype sequence (DRG1‐Lys), highlights the biocatalytic potential of JMJD7.

The observation that γ‐thialysine‐containing DRG1 peptide (DRG1‐CysNH_2_) sustains substantial JMJD7 activity, yielding a cysteamine product is of potential biological interest. Cysteamine is a natural product present in humans produced by breakdown of coenzyme A and is used as a drug to treat nephropathic cystinosis (Ariceta et al., 2015). Evidence has also been reported that γ‐thialysine is cytotoxic, at least in part because it induces apoptotic DNA fragmentations and interrupts the cell cycle progression (Jun et al., 2003). A formylglycine residue is present in Type I sulfatases, where it has a catalytically important role, that its hydrated form acts as a nucleophilic catalyst (Appel & Bertozzi, 2015). The JmjC hydroxylase FIH can also catalyze formylglycine formation by oxidation of a serine residue though the biological relevance of this observation is unclear (Yang et al., 2013). It is possible that the cytoxicity of γ‐thialysine in part results from its mis‐incorporation into proteins with subsequent JmjC / other enzyme catalyzed oxidation resulting in formation of formylglycine residues, with cytotoxic consequences for protein stability / reactivity.

## 4 METHODS

### 4.1 Peptide synthesis

DRG1 peptides (residues 16–40, ARTQKNKATAHHLGLLKARLAKLRR) were synthesized with C‐terminal amides on Rink amide resin (0.78 g mmol^−1^ loading) employing automated Fmoc‐SPPS chemistry on PurePep® Chorus Synthesizer. Coupling of amino acids were carried out adding a mixture of amino acid (3.0 eq) activated with hexafluorophosphate azabenzotriazole tetramethyl uronium (HATU) (3.0 eq) and *N*,*N*‐diisopropylethylamine (DIPEA) (5.0 eq) in dimethylformamide (DMF) at 75°C for 20 min; for Thr and Arg residues the coupling procedure was carried out twice. Natural or unnatural lysine analog amino acids were manually incorporated on resin with the 18‐mer DRG1 peptide at position 22 by standard HATU/DIPEA coupling overnight at room temperature. Fmoc deprotection was achieved by a solution of 20% (v/v) piperidine in DMF at 75°C for 7 min. Elongations after the incorporation of the lysine analogs were either carried out via manual couplings or automated synthesis. Manual couplings were performed via standard couplings for 1 h and deprotections for 20 min at room temperature. Fmoc‐Sec(Xan)‐OH (2.0 eq) was manually coupled employing DIC (2.0 eq) and Oxyma (2.0 eq); this procedure was performed twice. Automated elongation was carried out via standard couplings for 5 min and deprotections for 3 min at 90°C.

After coupling of the N‐terminal amino acid and final Fmoc‐deprotection, the peptides assembled on resin were washed with dichloromethane and dried over diethyl ether. The DRG1‐Lys peptide (residues 17–40) was synthesized using the same procedure. The peptides were cleaved from resin using 95% (v/v) trifluoroacetic acid (TFA), 2.5% (v/v) triisopropylsilane (TIPS) and 2.5% (v/v) milliQ (MQ) water for 4 h. DRG1‐Met/hMet peptides were cleaved in the presence of 10% (v/v) dimethyl sulfoxide (DMSO). DRG1‐Sec peptide was cleaved with 82.5% (v/v) TFA, 5% (v/v) phenol, 5% (v/v) MQ water, 5% (v/v) thioanisole and 2,5% (v/v) 1,2‐ethandithiol for 2.5 h. The crude peptides were precipitated with cold diethyl ether (−20°C) and pelleted via centrifugation. The crude DRG1 peptide analogs were purified by RP‐HPLC, and their purity was assessed by MALDI‐TOF MS and analytical RP‐HPLC (Figures S1–S20).

### 4.2 Synthesis of DRG1‐CysNH_2_ and CysNHme analogs

The DRG1‐Cys22 peptide was site‐specifically alkylated based on the reported protocol (Al Temimi, van der Wekken‐de, et al., 2019; Proietti et al., 2020). The DRG1‐Cys22 peptide was dissolved in a 350 μL alkylation buffer (4 M guanidine hydrochloride, 1 M HEPES pH 7.8 and 10 mM L‐methionine) and incubated for 1 h at 37°C under reducing conditions (20 μL 1 M DTT). The alkylation reagents 2‐bromo‐N‐methylethanamine hydrobromide (30 eq.) or 2‐bromoethylamine hydrobromide (40 eq.) were added to the reaction mixture, which was allowed to react for 2.5 h at 37°C. DTT (1 M, 3 μL) was added to the mixture and the reaction was allowed to proceed for another 2.5 h until full conversion was achieved as monitored by MALDI‐TOF MS. The reaction was quenched with 10 μL 2‐mercaptoethanol for 30 min at room temperature. The reaction sample was then diluted (1:2 v/v) and purified by RP‐HPLC.

### 4.3 JMJD7 expression and purification

A pET‐28a(+) plasmid encoding gene for human JMJD7 (residues 1–316) with an N‐terminal His_6_‐tag was transformed into competent *Escherichia coli* Rosetta (DE3) cells. The cells were cultivated in 2TY media supplemented with kanamycin (30 μg/mL) and chloramphenicol (35 μg/mL) at 37°C with agitation (180 rpm). Upon reaching an optical density of 0.6 at 600 nm, expression was induced by adding isopropyl‐β‐D‐thiogalactopyranoside (final concentration of 0.5 mM), followed by further incubation at 18°C for 16 h. The cells were then harvested by centrifugation (7900 rpm, 15 min, 4°C). The resulting cell pellet was stored at −80°C.

The frozen cell pellet was resuspended in ice‐cold 50 mM HEPES buffer (pH 7.5, 500 mM NaCl, 5 mM imidazole) supplemented with EDTA‐free protease inhibitor cocktail tablets (1 tablet per 50 mL, Roche Diagnostics Ltd) and DNAse I (bovine pancreas, Sigma‐Aldrich). Cell lysis was achieved using a high‐pressure cell breaker (EmulsiFlex‐C5, Avestin; three passages, 4°C) followed by centrifugation (58,545 ***g***, 30 min, 4°C). The resulting supernatant was subjected to purification at 4°C using Ni(II)‐affinity chromatography (HisTrap HP column, GE Healthcare) using an AKTA Pure machine (GE Healthcare) employing a step elution gradient (from 50 mM HEPES, pH 7.5, 500 mM NaCl, 40 mM imidazole to elution buffers containing 500 mM imidazole). Fractions containing JMJD7 were combined, concentrated using Amicon Ultra centrifugal filters (3082 ***g***, 4°C), and further purified by size‐exclusion chromatography utilizing a HiLoad 26/60 Superdex 75 pg 300 mL column with a flow rate of 1 mL/min and 50 mM HEPES, pH 7.5, 200 mM NaCl, 2% glycerol for storage at −80°C until further use.

### 4.4 LC–MS enzymatic assays

All reagents were procured from Sigma Aldrich. 2‐Oxoglutarate (2OG, 10 mM) and L‐ascorbic acid (LAA, 50 mM) solutions were prepared freshly by dissolving the solids in deionized water. Ferrous ammonium sulfate (FAS) solution was prepared freshly by dissolving FAS at 400 mM in 20 mM HCl with subsequent dilution to 1 mM using deionized water. JMJD7 assays using DRG1 (16–40) based peptides were performed with the following conditions: (i) 2 or 5 μM JMJD7, 10 μM DRG1 peptide, 100 μM LAA, 10 μM FAS, 10 μM 2OG in mM 50 Tris‐buffer (pH 7.5), (ii) 2 μM JMJD7, 10 μM DRG1 peptide, 100 μM LAA, 10 μM FAS, 20 μM 2OG in mM 50 Tris‐buffer (pH 7.5). Reactions were initiated by addition of enzyme to the cosubstrate/substrate solution in a final reaction volume of 50 μL with 2 h incubation at room temperature. A positive control reaction containing enzyme and a validated peptide substrate was included in all screening studies. After 2 h, reactions were stopped by addition of 5 μL of 10% (v/v) LC–MS grade formic acid (Fisher Scientific) and transferred to a 96 well plate (Agilent). Intact mass peptide analysis was performed by LC–MS using an Agilent 1290 infinity II LC system equipped with an Agilent 1290 multisampler and an Agilent 1290 high speed pump and connected to an Agilent 6550 accurate mass iFunnel quadrupole time of flight (Q‐TOF) mass spectrometer. The reaction mixture (4 μL) was injected and loaded onto a ZORBAX RRHD Eclipse Plus C18 column (Agilent). Solvent A consisted of LC–MS grade water containing 0.1% (v/v) formic acid and solvent B consisted of MeCN containing 0.1% (v/v) formic acid. Peptides were separated using a step wise gradient (0 min – 95% solvent A, 1.0 min – 80% solvent A, 3.0 min – 45% solvent A, 4.0 min – 45% solvent A, 5.0 min – 0% solvent A, 6.0 min – 0% solvent A, 7.0 min – 95% solvent A). This was followed by a 3 min elution with 95% solvent A to re‐equilibrate the column. Flow rates were 0.2 mL/min. The mass spectrometer was operated in the positive ion mode with a drying gas temperature (280°C), drying gas flow rate (13 L min^−1^), nebulizer pressure (40 psig), sheath gas temperature (350°C), sheath gas flow rate (12 L min^−1^), capillary voltage (4000 V), nozzle voltage (1000 V). Data were analyzed using Agilent MassHunter Qualitative Analysis (Version B.07.00) software.

### 4.5 JMJD7 kinetic assays

JMJD7 turnover assays for kinetic studies were performed in 96‐well polypropylene assay plates with 1.0 mL total reaction volume using a series of concentrations of the DRG1 peptide (0.1–5.0 μM), 2OG (20 μM), FAS (10 μM), LAA (100 μM). Reactions were initiated by the addition of JMJD7 (100 nM). MS‐analyses were performed using a RapidFire RF 365 high‐throughput sampling robot (Agilent) attached to an iFunnel Agilent 6550 accurate mass quadrupole time‐of‐flight (Q‐TOF) mass spectrometer operated in the positive ionization mode. The RapidFire RF 365 high‐throughput sampling robot was programmed to aspirate samples from the reaction mixture at the indicated time intervals. Assay samples were loaded onto a C4 solid phase extraction (SPE) cartridge and peptides were eluted using given previous protocols (Tumber et al., 2023). The % conversion of the hydroxylation reaction were calculated from the ion chromatogram data by integrating peak areas using RapidFire Integrator 4.3.0 software (Agilent).

### 4.6 Chemoselective oxidation reactions with Selectfluor

DRG1 peptides (Ser, MetO, Met, hMet, 10 μM) were incubated in Tris‐buffer (50 mM) at pH 8.0 in the presence of 20 μM 2OG, 20 μM FAS and 50 μM LAA in 2 h. In parallel, the JMJD7‐catalyzed (2 μM) reaction of DRG1‐Met and DRG1‐hMet (10 μM) was also carried out under same conditions. All samples were then lyophilized and reacted with Selectfluor (100 μM) in the presence of pyridine (50 μM) in phosphate buffer (10 mM) at pH 7.0 for 1 h at room temperature. Reaction products were monitored by MALDI‐TIMS‐TOF‐MS or MALDI‐TOF‐MS.

### 4.7 NMR assays

Stock solutions of L‐ascorbate (LAA; 500 mM), 2‐oxoglutarate (2OG; 20 mM), ferrous ammonium sulphate (FAS; 100 mM), and (trimethylsilyl)propionic‐2,2,3,3‐*d*_*4*_ (TSP‐*d*_*4*_; 8 mM) were freshly prepared in water on the day NMR assays were run. These stock solutions of LAA, FAS, and 2OG were diluted using aqueous buffer (50 mM sodium phosphate, pH 7.4, 10% v/v D_2_O) to give FAS (0.3 mM), 2OG (2 mM), and LAA (5 mM). DRG1 peptides were solubilized in buffer to a final concentration of 2 mM for use in NMR assays. JMJD7 was buffer exchanged to 50 mM sodium phosphate buffer (pH 7.4, 10% v/v D_2_O) using 30 kDa Millipore Amicon Ultra‐4 centrifugal filter units. Assays were performed in 3 mM NMR tubes (Bruker, 2,001,724) with a constant total reaction volume of 180 μL using the following assay conditions – 500 μM LAA, 200 μM 2OG, 200 μM peptide, 30 μM FAS, 800 μM TSP‐*d*_*4*_ and 10 μM JMJD7. The stock solutions were added to a 1.5 mL Eppendorf tube in the following order: deuterated buffer (variable volume), LAA (5 mM; 18 μL), 2OG (2 mM; 18 μL), peptides (2 mM; 18 μL), FAS (0.3 mM; 18 μL), TSP‐*d*_*4*_ (8 mM, 18 μL), and JMJD7 (variable volume). The components were thoroughly mixed, transferred to an NMR tube, and hand‐centrifuged. NMR studies were conducted at 298 K using a Bruker AVIII 600 MHz spectrometer quipped with a ^1^H TCI‐inverse cryoprobe optimized for ^1^H observation on Bruker Topspin 4.3.1. The probe was locked to 10% (v/v) D_2_O in water and auto‐tuned and matched sing “atma” on Topspin. The sample was then shimmed (topshim) and the receiver gain (rg) was adjusted to 203. Consecutive acquisitions were started using ‘multizg’ command for 64 scans per acquisition to increase signal‐to‐noise ratio. Turnover was quantified using TSP‐*d*_*4*_ as an internal standard.

### 4.8 JMJD7 inhibition assays

Single point screening assays were as follows; initially, JMJD7 (200 nM) and substituted DRG1 peptide (10 or 50 μM) were incubated for 15 min, followed by the addition of a mixture of the DRG1‐Lys substrate (5 μM), 2OG (10 μM), FAS (10 μM) and LAA (100 μM) in a final volume of 50 μL in Tris‐buffer (pH 7.5) and incubated for additional 25 min at room temperature. IC_50_ measurements were carried out with DRG1 peptides (1 nM–150 μM), 2OG (10 μM), FAS (10 μM) and LAA (100 μM). Reactions were quenched by the addition of 10% (v/v) formic acid and mixed 1:1 with CHCCA matrix (1:1 v/v MeCN:H_2_O) then analyzed by MALDI‐TIMS‐TOF MS. JMJD7 activity was determined by calculating the integral of hydroxylated peptide and normalized to a control reaction in absence of potential inhibitory peptides. Every experiment was performed in duplicate (*n* = 2), error bars are reported as SE.

## AUTHOR CONTRIBUTIONS

**Nurgül Bilgin:** Investigation; methodology; writing – original draft. **Anthony Tumber:** Investigation; methodology. **Siddhant Dhingra:** Methodology. **Eidarus Salah:** Methodology. **Aziza Al‐Salmy:** Methodology. **Sandra Pinzón Martín:** Methodology. **Yicheng Wang:** Methodology. **Christopher J. Schofield:** Funding acquisition; investigation; methodology; supervision; writing – original draft. **Jasmin Mecinović:** Funding acquisition; investigation; methodology; supervision; writing – original draft.

## CONFLICT OF INTEREST STATEMENT

The authors declare that there are no conflicts of interest.

## Supporting information


Appendix S1.
