# Supplementary material for: Substrate selectivity and inhibition of the human lysyl hydroxylase JMJD7
Source: Protein Sci. 2024 Sep 14;33(10):e5162. doi: 10.1002/pro.5162 (PMC11400632; doi:10.1002/pro.5162)
Supplement: Supplementary file 1 — Appendix S1. [file PRO-33-e5162-s001.docx]

**Supporting Information**

**Substrate selectivity and inhibition of the human lysyl hydroxylase JMJD7**

Nurgül Bilgin,^[a]^ Anthony Tumber,^[b]^ Siddhant Dhingra,^[b]^ Eidarus Salah,^[b]^ Aziza Al-Salmy^[a]^, Sandra Pinzón Martín,^[a]^ Yicheng Wang,^[b]^ Christopher J. Schofield*^[b]^ and Jasmin Mecinović*^[a]^

^[a]^ Department of Physics, Chemistry and Pharmacy, University of Southern Denmark, Campusvej 55, 5230 Odense, Denmark.

^[b]^ Chemistry Research Laboratory, Department of Chemistry and the Ineos Oxford Institute for Antimicrobial Research, University of Oxford, 12 Mansfield Road, OX1 3TA Oxford, United Kingdom.

E-mail: [mecinovic@sdu.dk](mailto:%20), [christopher.schofield@ch](mailto:christopher.schofield@ch)em.ox.ac.uk

Table of Contents

1. MALDI-TOF MS characterization and analytical HPLC data 3

2. Hydroxylation reaction of DRG1-CysNH_2_ 33

3. LC-MS assays of lysine analogs 34

4. NMR supporting figures 36

5. Supporting LC-MS assay data 39

**1. MALDI-TOF MS characterization and analytical HPLC data**

Table S1 Synthetic DRG1 peptides used in this work.

| Peptide name | Sequence | Monoisotopic  m/z  observed | Monoisotopic  m/z calculated |
| --- | --- | --- | --- |
| DRG1-Lys | ARTQKNLysATAHHLGLLKARLAKLRR | 2849.9 | 2849.7 |
| DRG1-D-Lys | ARTQKND-LysATAHHLGLLKARLAKLRR | 2850.3 | 2849.7 |
| DRG1-Orn | ARTQKNOrnATAHHLGLLKARLAKLRR | 2836.0 | 2836.7 |
| DRG1-hLys | ARTQKNhLysATAHHLGLLKARLAKLRR | 2864.1 | 2863.8 |
| DRG1-LysMe | ARTQKNLysMeATAHHLGLLKARLAKLRR | 2863.9 | 2863.8 |
| DRG1-Ahp | ARTQKNAhpATAHHLGLLKARLAKLRR | 2848.8 | 2848.8 |
| DRG1-HNle | ARTQKNHNleATAHHLGLLKARLAKLRR | 2850.9 | 2850.7 |
| DRG1-CysNH_2_ | ARTQKNCysNH_2_ATAHHLGLLKARLAKLRR | 2867.3 | 2867.7 |
| DRG1-CysNHMe | ARTQKNCysNHmeATAHHLGLLKARLAKLRR | 2880.8 | 2880.7 |
| DRG1-LysN | ARTQKNLysNATAHHLGLLKARLAKLRR | 2850.0 | 2850.7 |
| DRG1-LysE | ARTQKNLysEATAHHLGLLKARLAKLRR | 2847.8 | 2847.8 |
| DRG1-hGln | ARTQKNhGlnATAHHLGLLKARLAKLRR | 2863.4 | 2863.7 |
| DRG1-nArg | ARTQKNnArgATAHHLGLLKARLAKLRR | 2864.1 | 2864.7 |
| DRG1-4pyrA | ARTQKN(4pyrA)ATAHHLGLLKARLAKLRR | 2870.1 | 2870.7 |
| DRG1-βhLys | ARTQKNβhLysATAHHLGLLKARLAKLRR | 2862.9 | 2862.8 |
| DRG1-LysCMe | ARTQKNLysCMeATAHHLGLLKARLAKLRR | 2862.8 | 2862.8 |
| DRG1-LysNMe | ARTQKNLysNMeATAHHLGLLKARLAKLRR | 2862.9 | 2862.8 |
| DRG1-Abg | ARTQKNAbgATAHHLGLLKARLAKLRR | 2849.2 | 2849.8 |
| DRG1-Cys | ARTQKNCysATAHHLGLLKARLAKLRR | 2824.3 | 2824.7 |
| DRG1-D-Cys | ARTQKN(D-Cys)ATAHHLGLLKARLAKLRR | 2825.5 | 2825.7 |
| DRG1-hCys | ARTQKNhCysATAHHLGLLKARLAKLRR | 2839.1 | 2838.8 |
| DRG1-CysMe | ARTQKNCysMeATAHHLGLLKARLAKLRR | 2839.5 | 2839.8 |
| DRG1-Met | ARTQKNMetATAHHLGLLKARLAKLRR | 2853.8 | 2853.8 |
| DRG1-hMet | ARTQKNhMetATAHHLGLLKARLAKLRR | 2867.3 | 2867.7 |
| DRG1-MetO | ARTQKN(MetO)ATAHHLGLLKARLAKLRR | 2868.9 | 2868.9 |
| DRG1-SeMet | ARTQKNSeMetATAHHLGLLKARLAKLRR | 2901.3 | 2901.6 |
| DRG1-Sec | ARTQKNSecATAHHLGLLKARLAKLRR | 2872.4 | 2872.4 |
| DRG1-Ser | ARTQKNSerATAHHLGLLKARLAKLRR | 2809.7 | 2809.8 |
| DRG1-hSer | ARTQKNhSerATAHHLGLLKARLAKLRR | 2822.8 | 2822.7 |
| DRG1-meOhSer | ARTQKNmeOhSerATAHHLGLLKARLAKLRR | 2837.2 | 2836.7 |


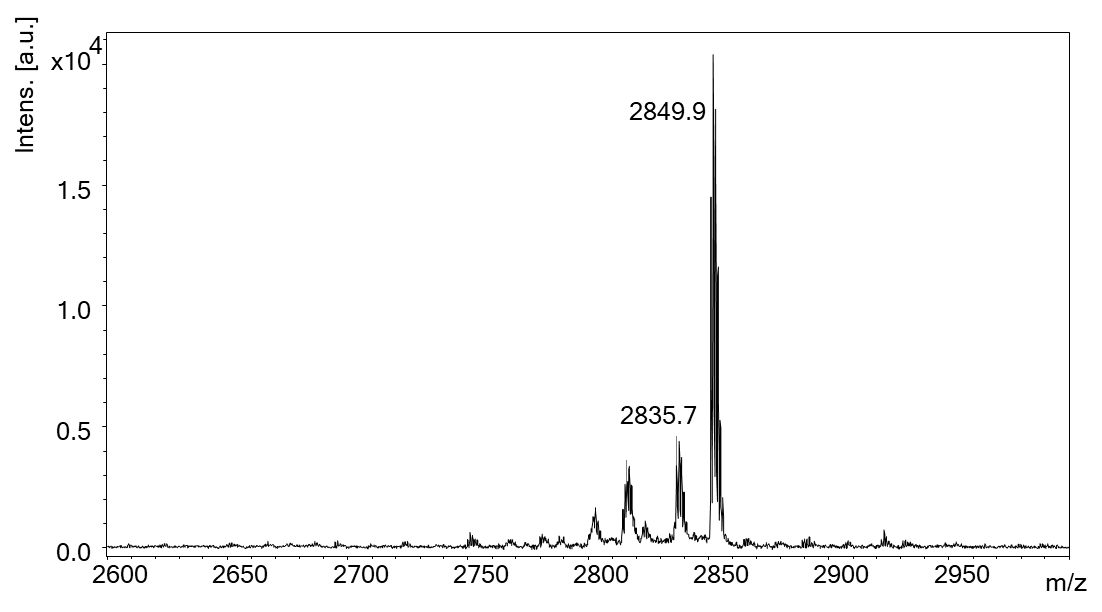


**
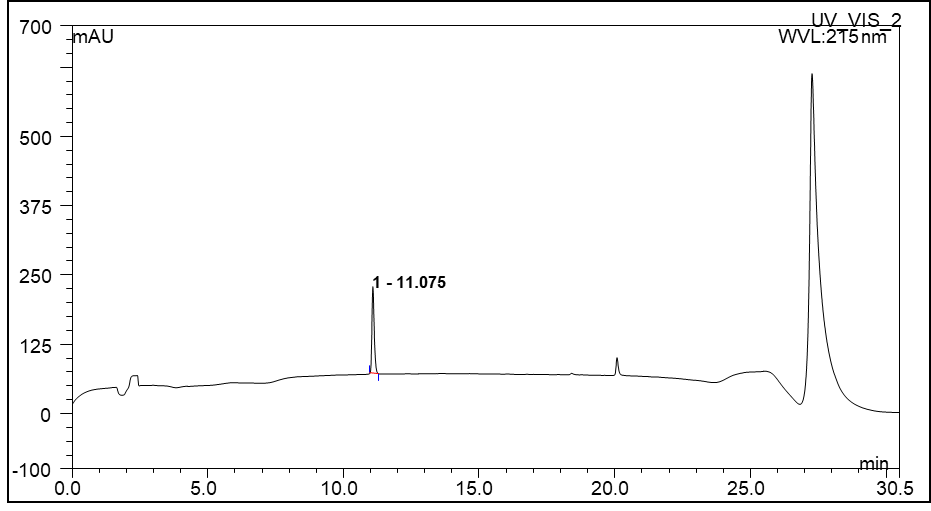
**

Figure S1 MALDI-TOF MS data for the DRG1-Lys peptide (*m/z observed* 2849.9) and analytical HPLC for the DRG1-Lys peptide after RP-HPLC purification. The peptide elutes at 11.1 min. Note that M-14 and M-36 peaks observed in Figures S1-30 are MALDI-TOF-MS artifacts.


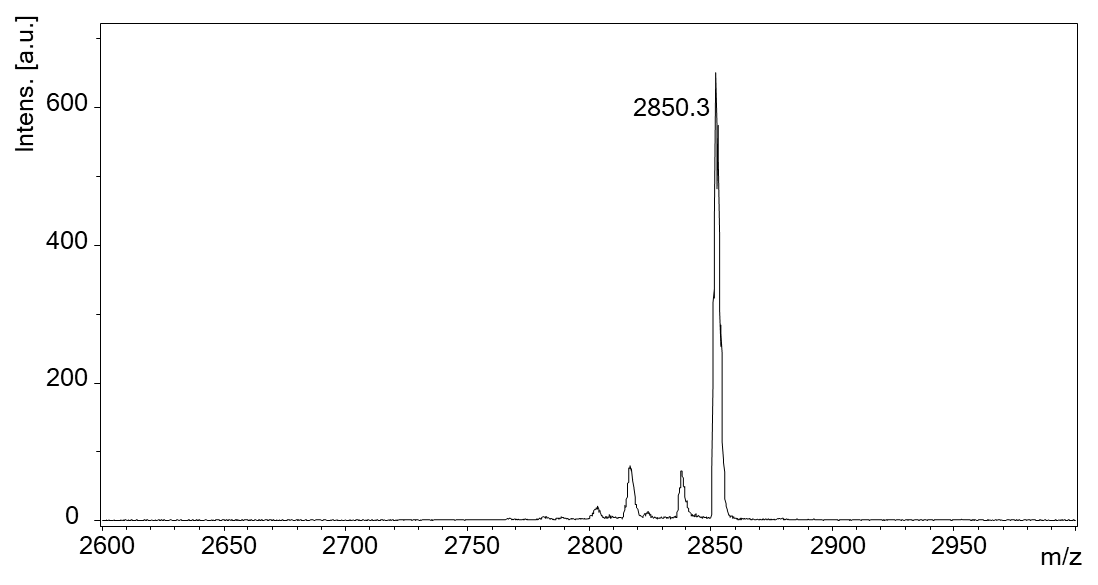


**
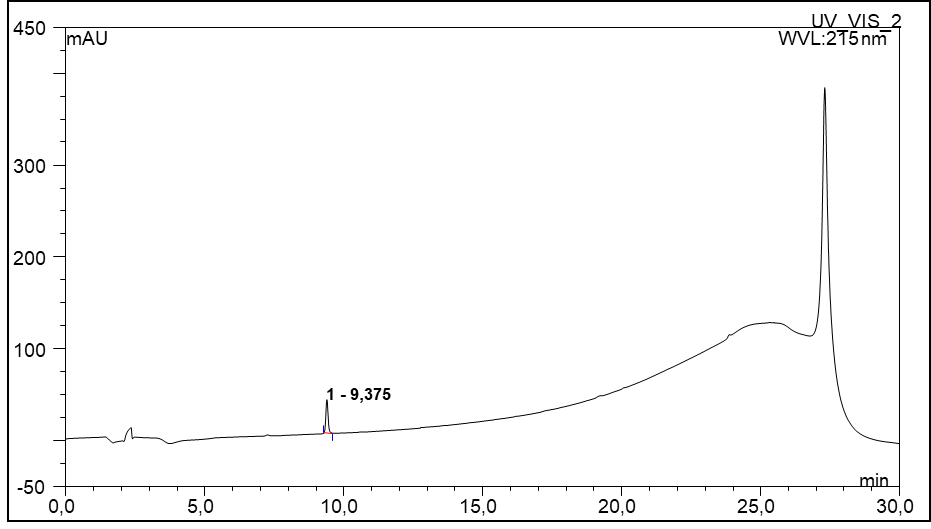
**

Figure S2 MALDI-TOF MS data for the DRG1-D-Lys peptide (*m/z observed* 2850.3) and analytical HPLC for the DRG1-D-Lys peptide after RP-HPLC purification. The peptide elutes at 9.4 min.


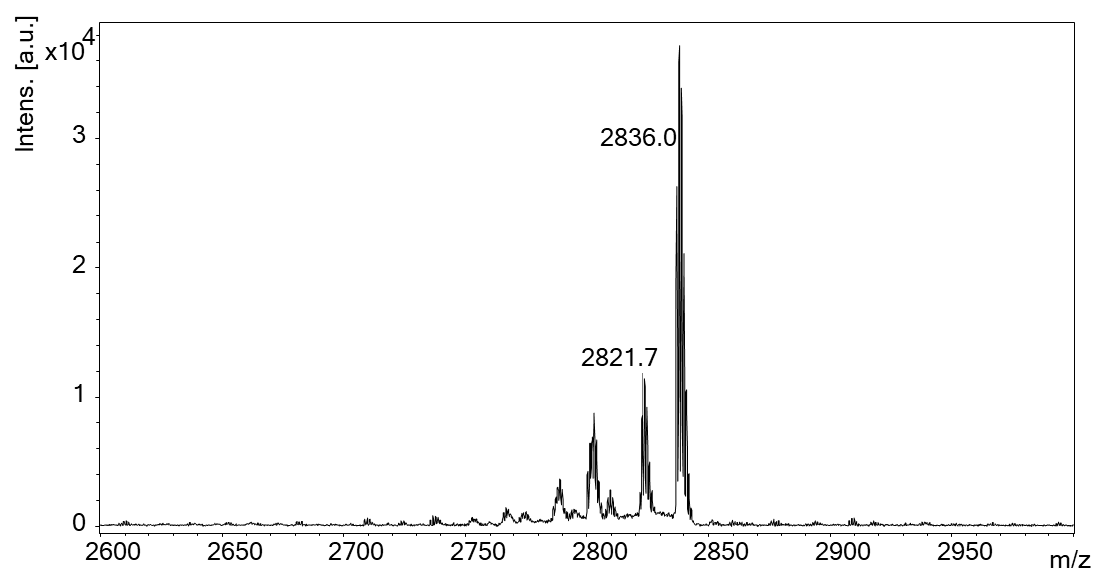


**
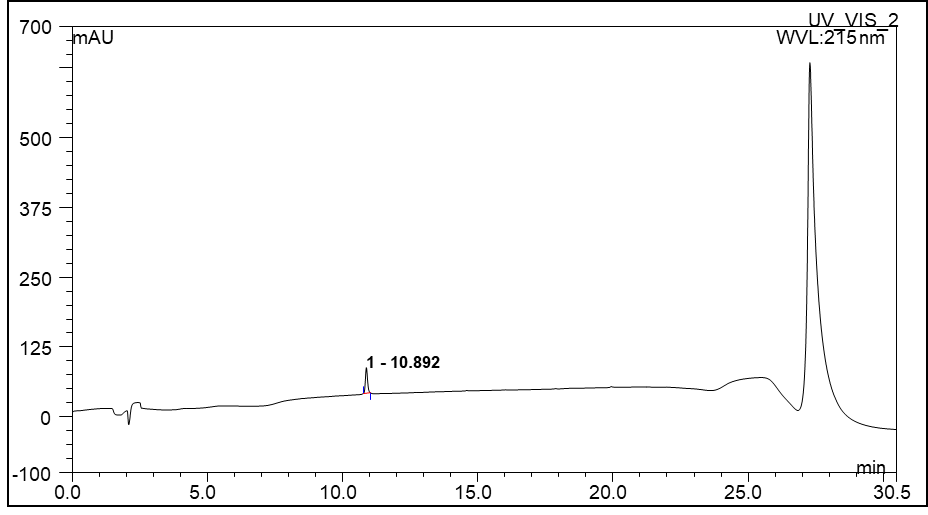
**

Figure S3 MALDI-TOF MS data for the DRG1-Orn peptide (*m/z observed* 2836.0) and analytical HPLC for the DRG1-Orn peptide after RP-HPLC purification. The peptide elutes at 10.9 min.

.


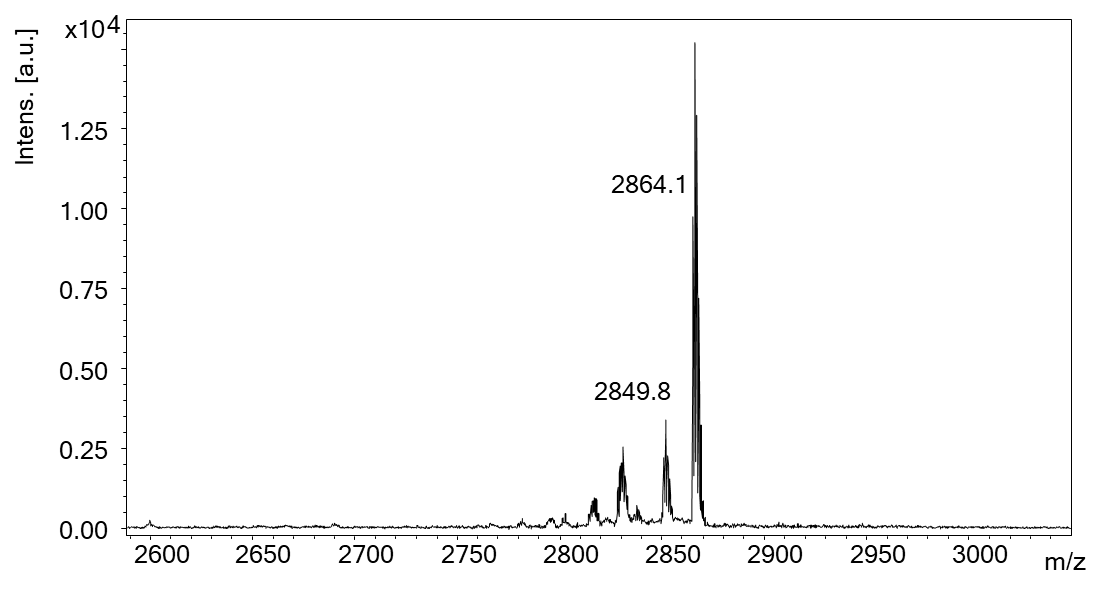


**
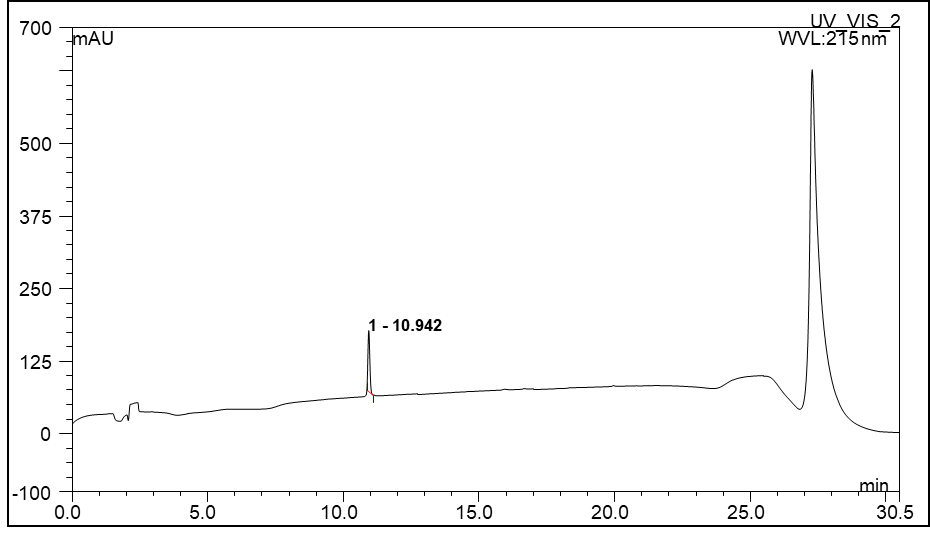
**

Figure S4 MALDI-TOF MS data for the DRG1-hLys peptide (*m/z observed* 2864.1) and analytical HPLC for the DRG1-hLys peptide after RP-HPLC purification. The peptide elutes at 10.9 min.


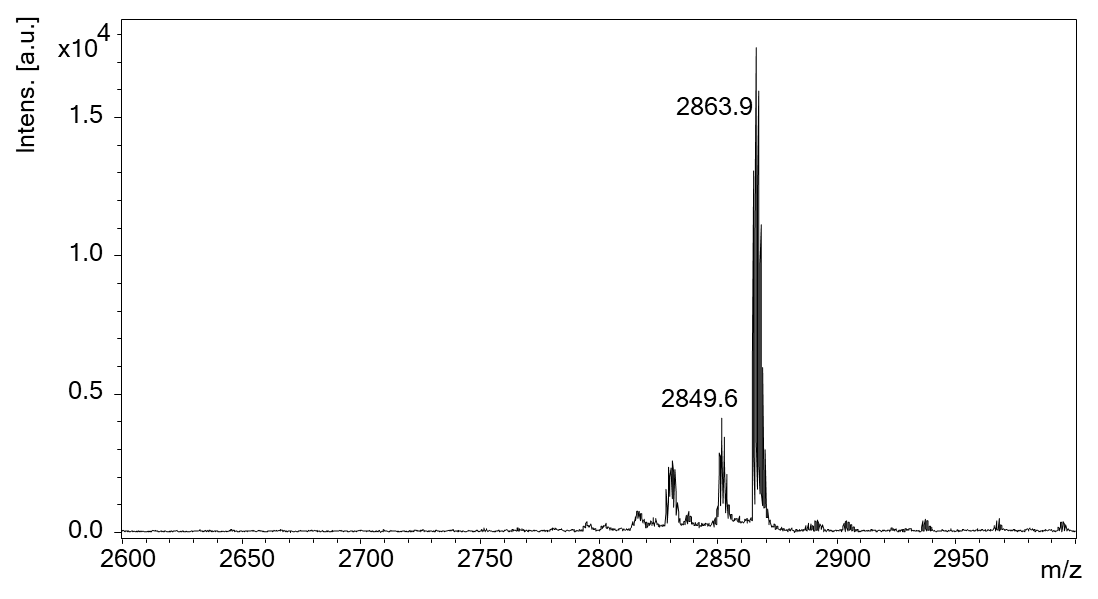


**
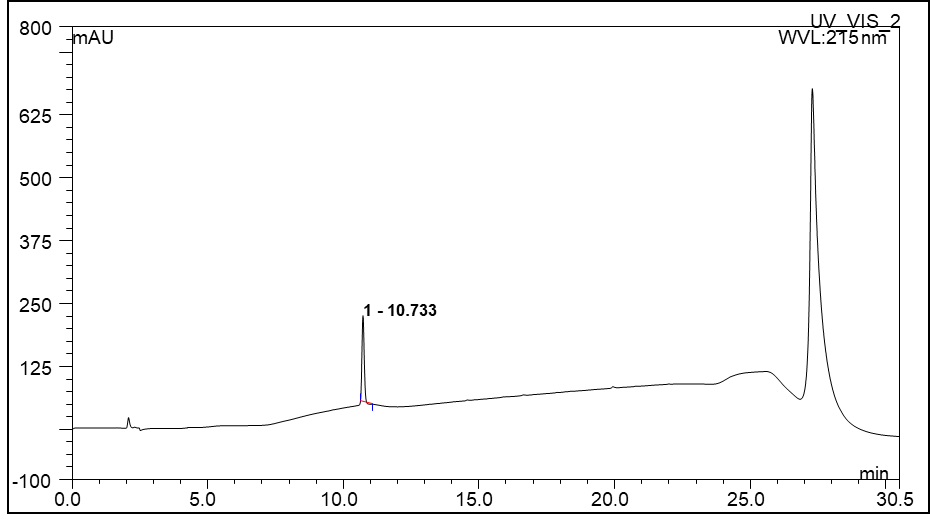
**

Figure S5 MALDI-TOF MS data for the DRG1-LysMe peptide (*m/z observed* 2863.9) and analytical HPLC for the DRG1-LysMe peptide after RP-HPLC purification. The peptide elutes at 10.7 min.


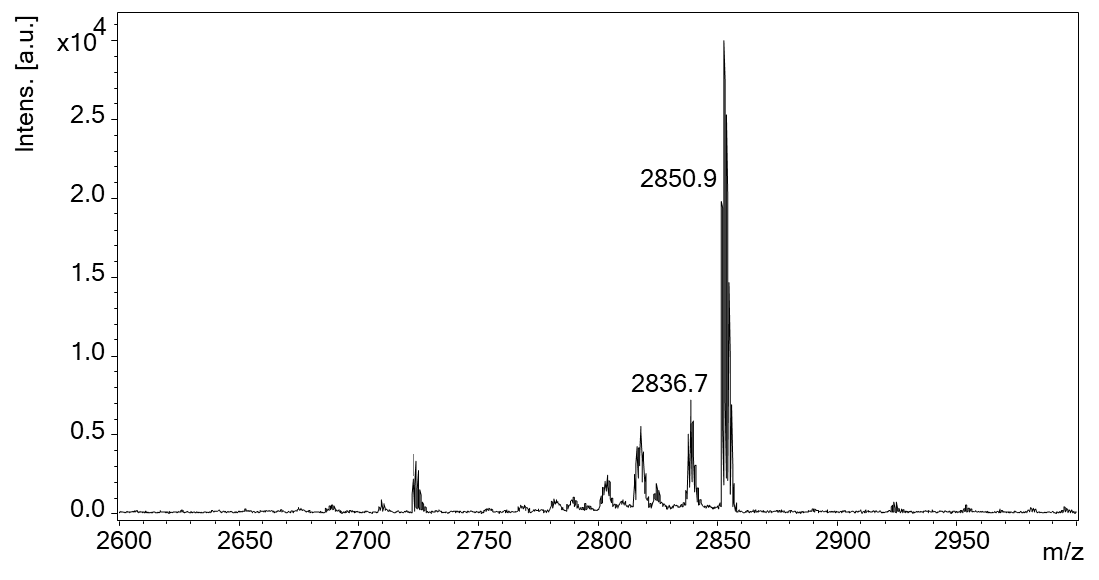


**
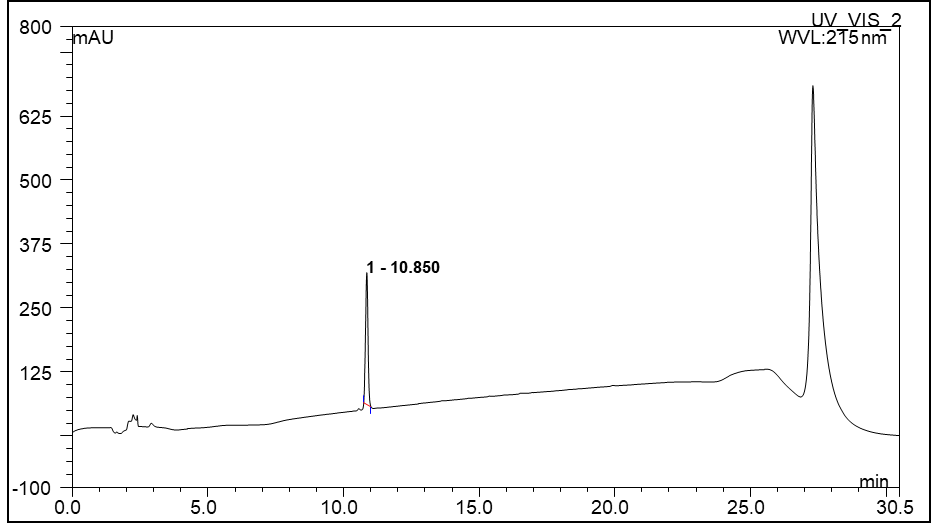
**

Figure S6 MALDI-TOF MS data for the DRG1-HNle peptide (*m/z observed* 2850.9) and analytical HPLC for the DRG1-HNle peptide after RP-HPLC purification. The peptide elutes at 10.9 min.


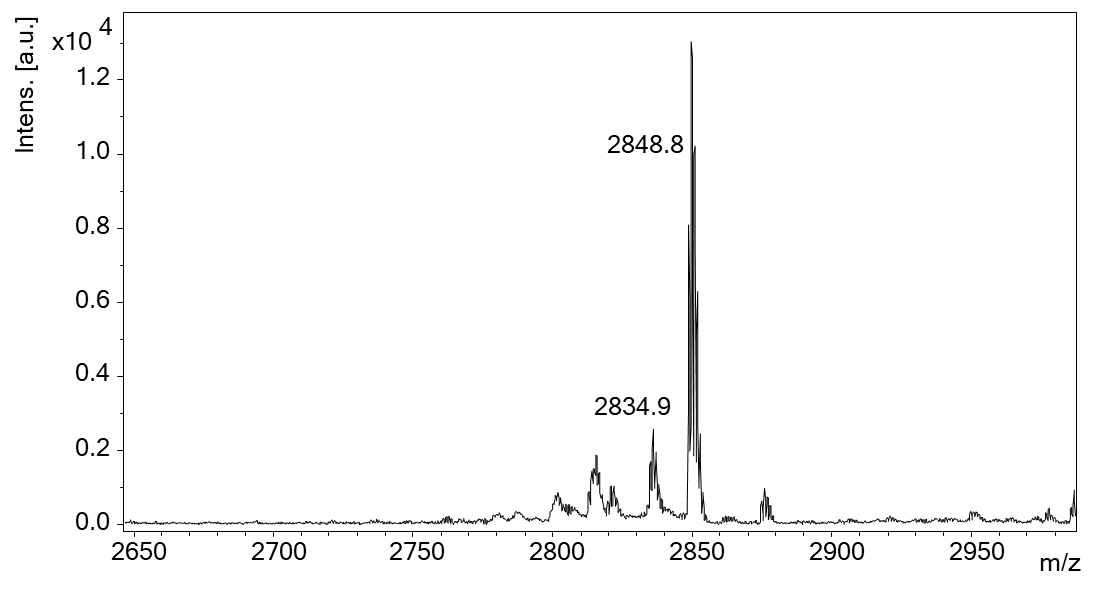


**
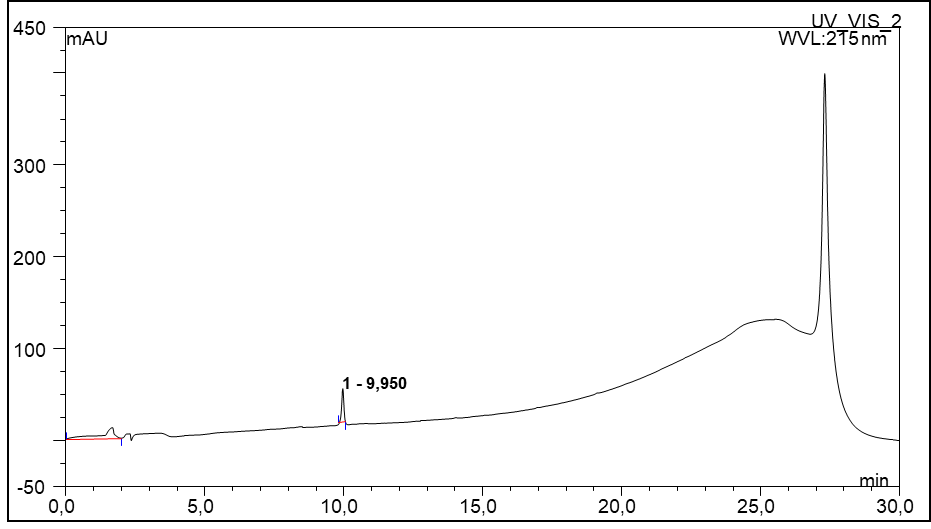
**

Figure S7 MALDI-TOF MS data for the DRG1-Ahp peptide (*m/z observed* 2848.8) and analytical HPLC for the DRG1-Ahp peptide after RP-HPLC purification. The peptide elutes at 10.9 min.


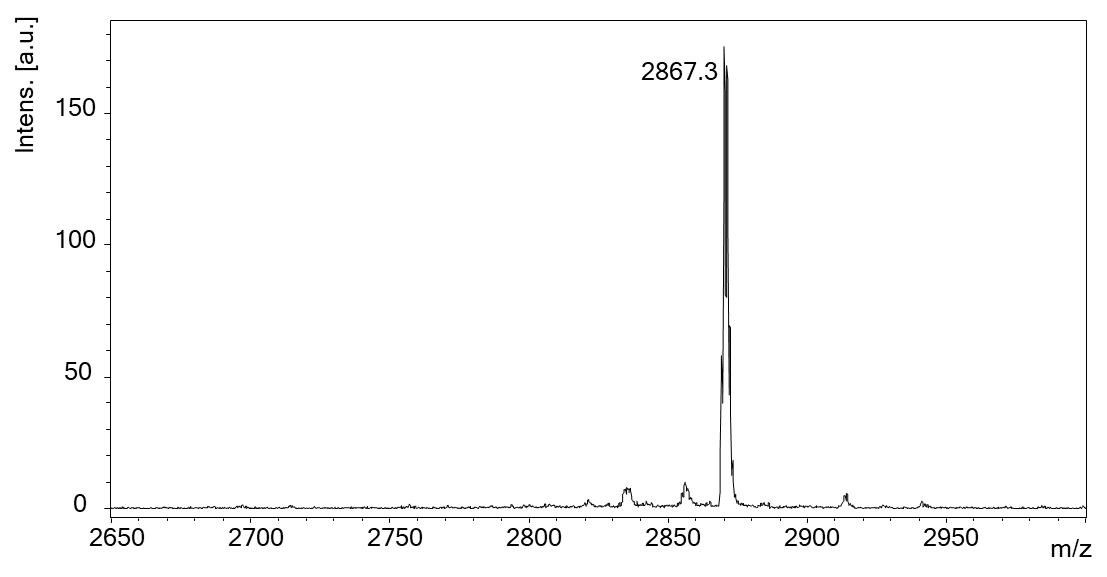


Figure S8 MALDI-TOF MS data for the DRG1-CysNH_2_ peptide (*m/z observed* 2867.3).


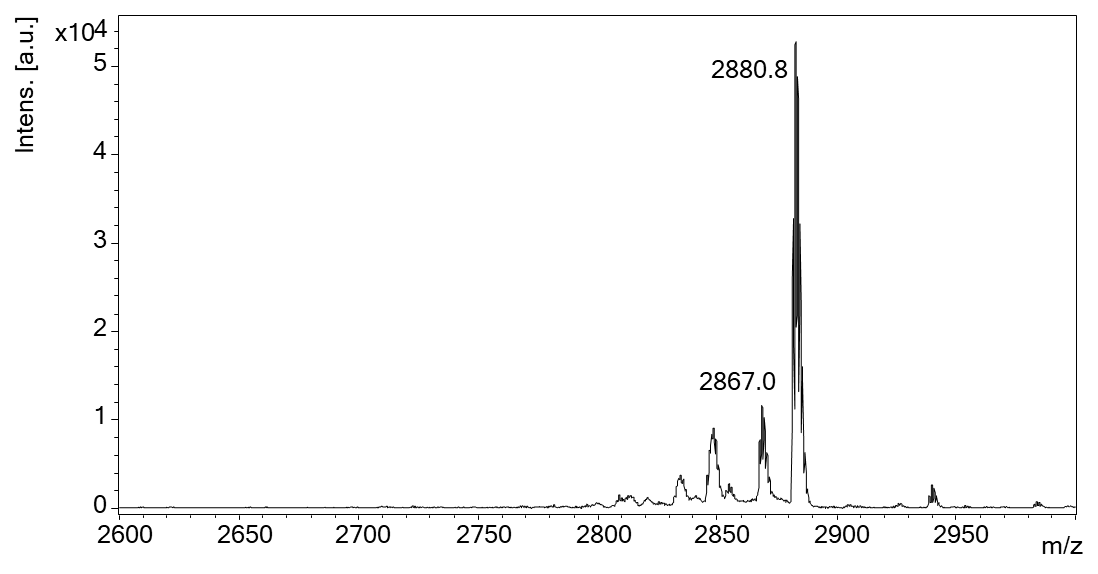


Figure S9 MALDI-TOF MS data for the DRG1-CysNHMe peptide (*m/z observed* 2880.8).


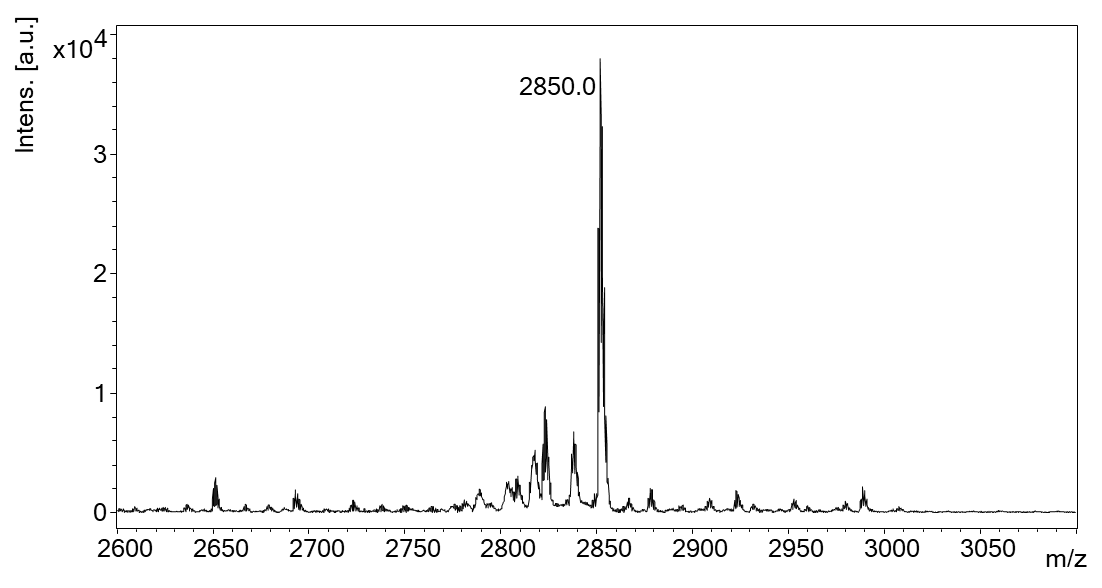


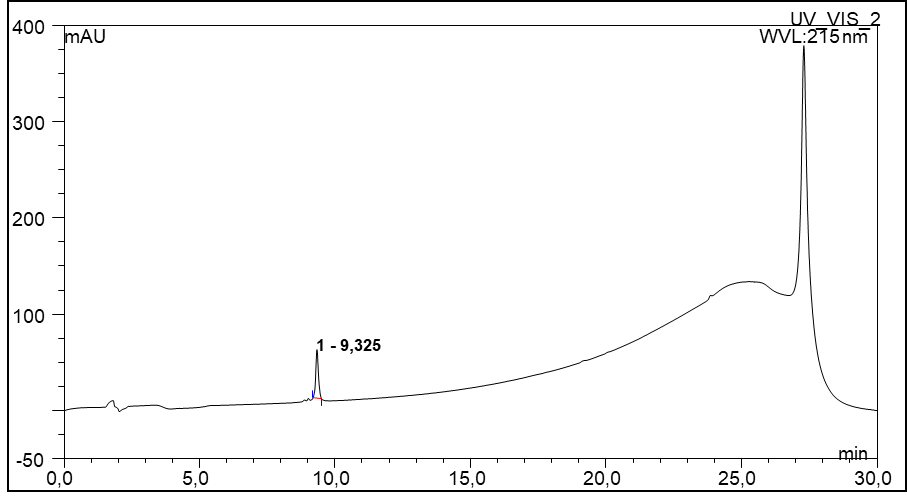


Figure S10 MALDI-TOF MS data for the DRG1-LysN peptide (*m/z observed* 2850.0) and analytical HPLC for the DRG1-LysN peptide after RP-HPLC purification. The peptide elutes at 9.3 min.


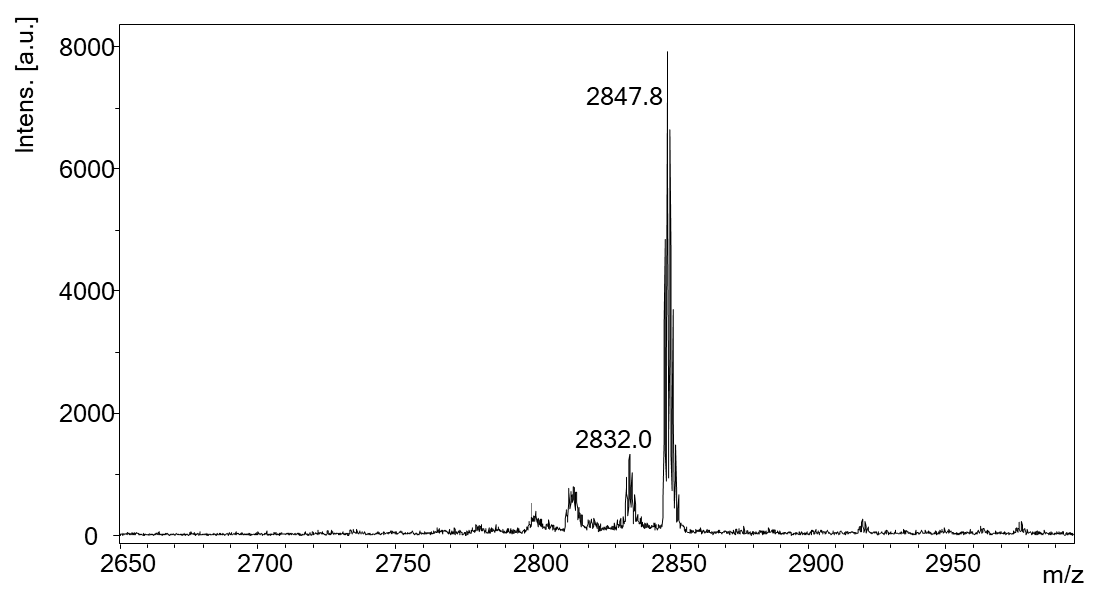


**
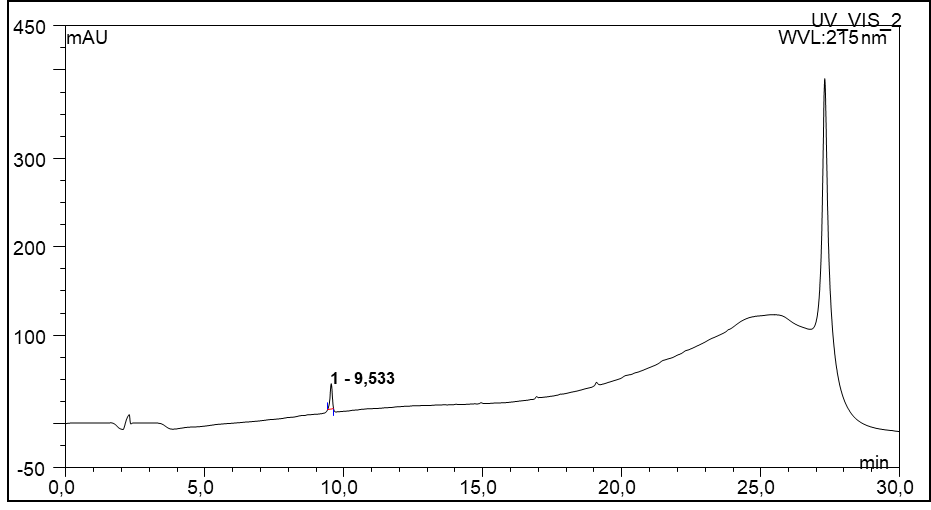
**

Figure S11 MALDI-TOF MS data for the DRG1-LysE peptide (*m/z observed* 2847.8) and analytical HPLC for the DRG1-LysE peptide after RP-HPLC purification. The peptide elutes at 9.5 min.


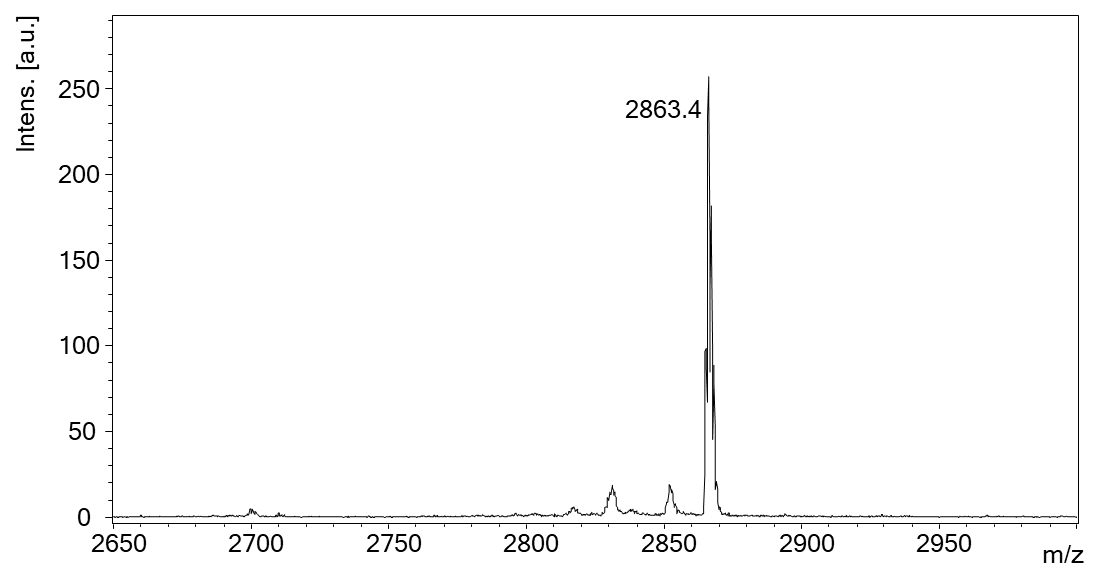


**
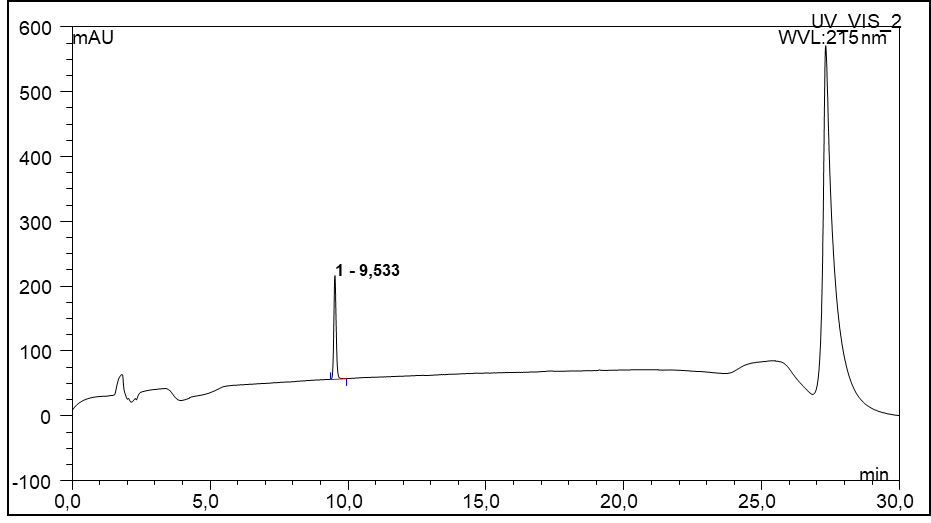
**

Figure S12 MALDI-TOF MS data for the DRG1-hGln peptide (*m/z observed* 2863.4) and analytical HPLC for the DRG1-hGln peptide after RP-HPLC purification. The peptide elutes at 9.5 min.


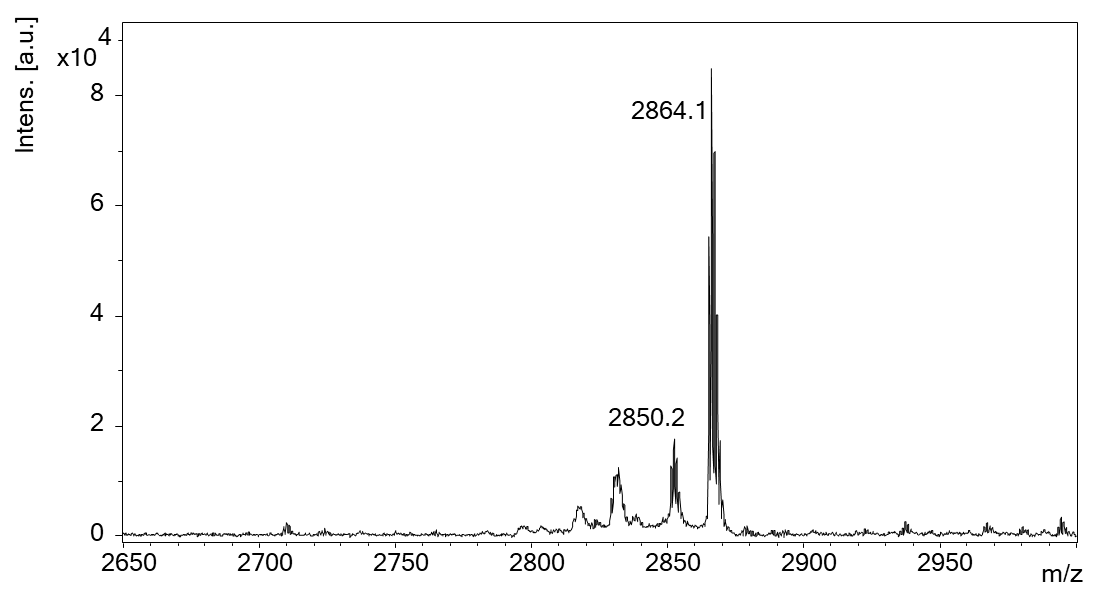


**
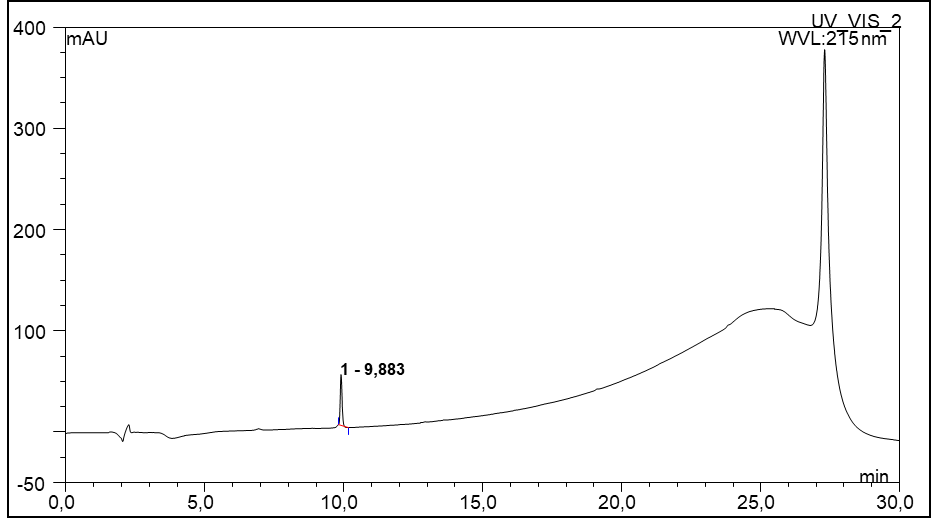
**

Figure S13 MALDI-TOF MS data for the DRG1-nArg peptide (*m/z observed* 2864.1) and analytical HPLC for the DRG1-nArg peptide after RP-HPLC purification. The peptide elutes at 9.9 min.


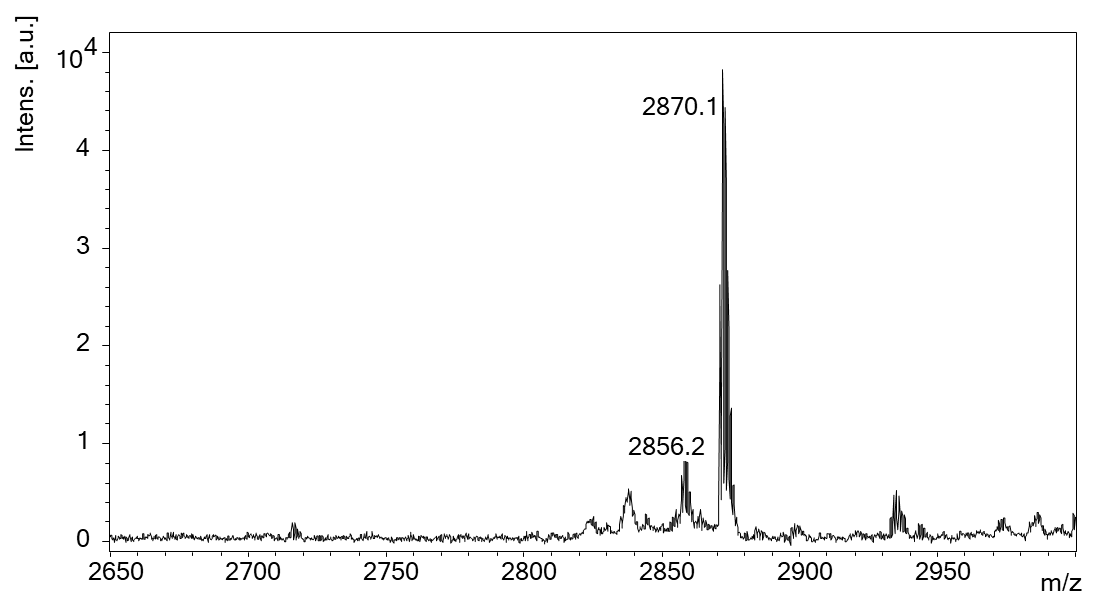


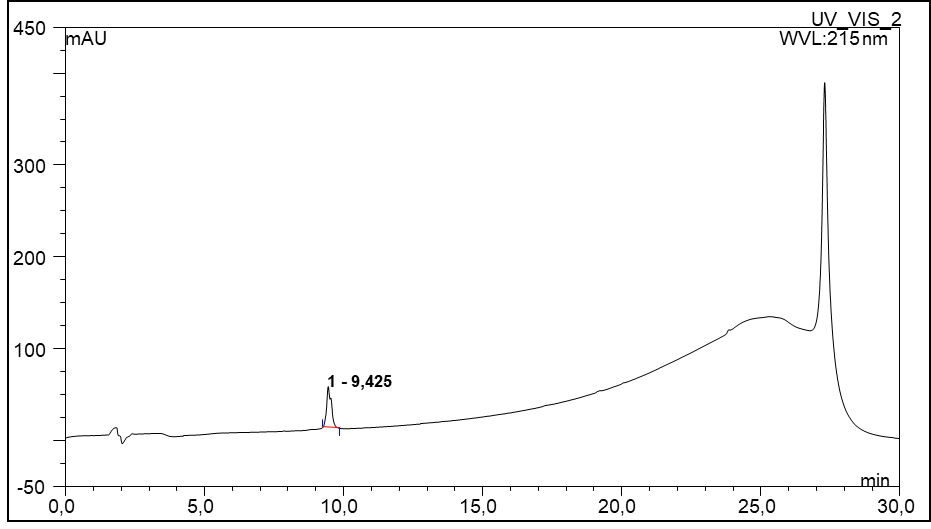


Figure S14 MALDI-TOF MS data for the DRG1-4pyrA peptide (*m/z observed* 2870.1) and and analytical HPLC for the DRG1-4pyrA peptide after RP-HPLC purification. The peptide elutes at 9.4 min.


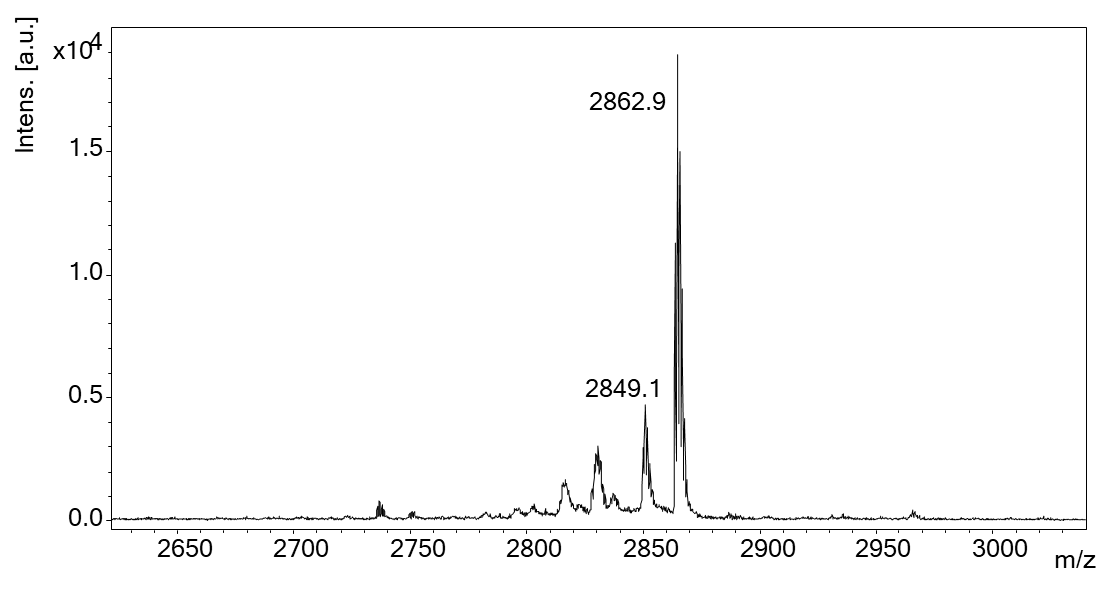


**
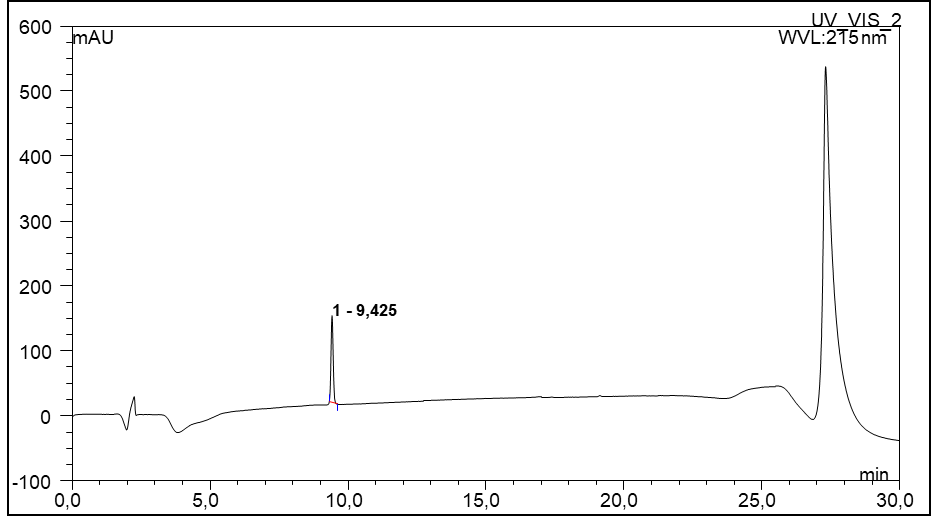
**

Figure S15 MALDI-TOF MS data for the DRG1-βhLys peptide (*m/z observed* 2862.9) and analytical HPLC for the DRG1-βhLys peptide after RP-HPLC purification. The peptide elutes at 9.4 min.


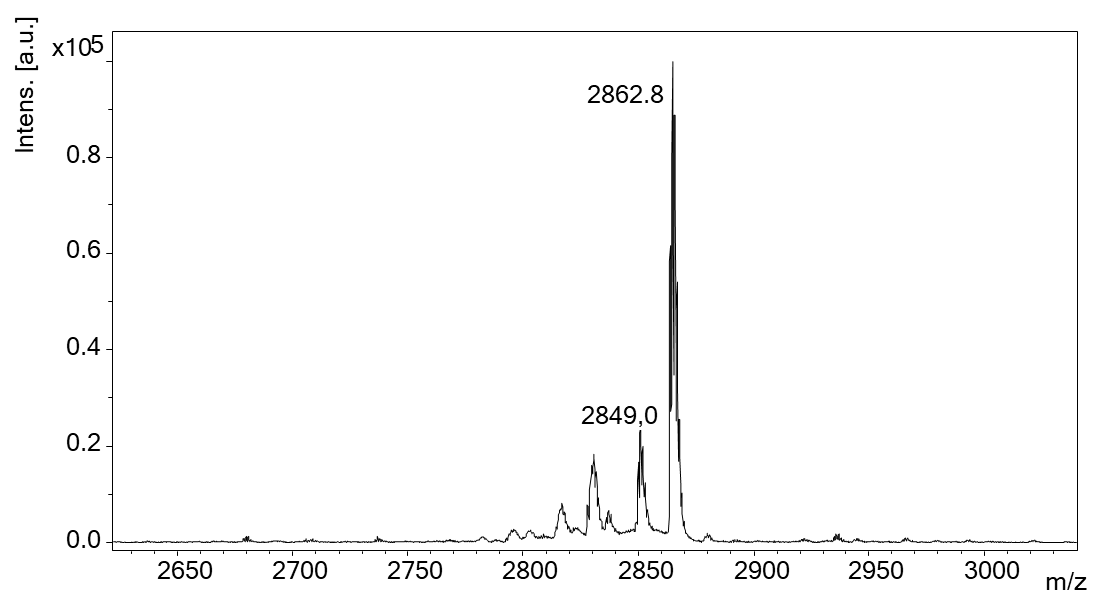


**
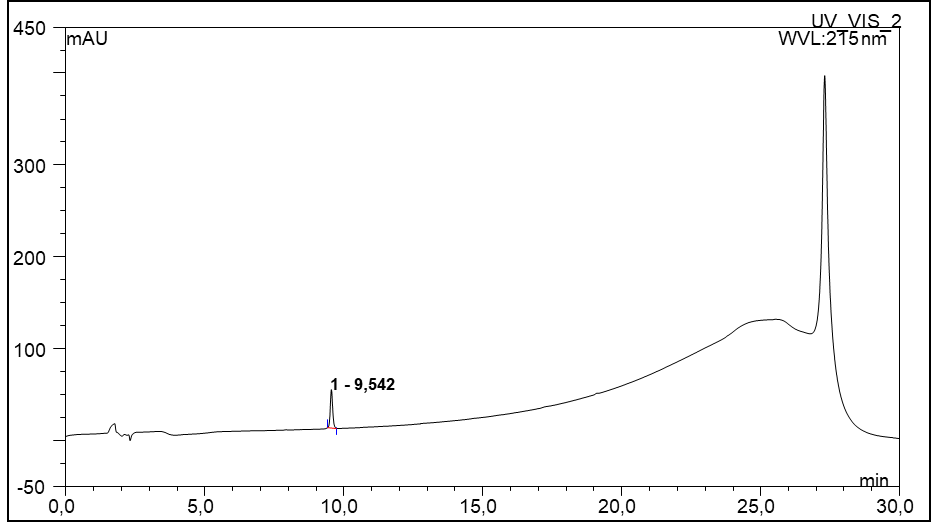
**

Figure S16 MALDI-TOF MS data for the DRG1-LysCMe peptide (*m/z observed* 2862.8) and analytical HPLC for the DRG1-LysCMe peptide after RP-HPLC purification. The peptide elutes at 9.5 min.


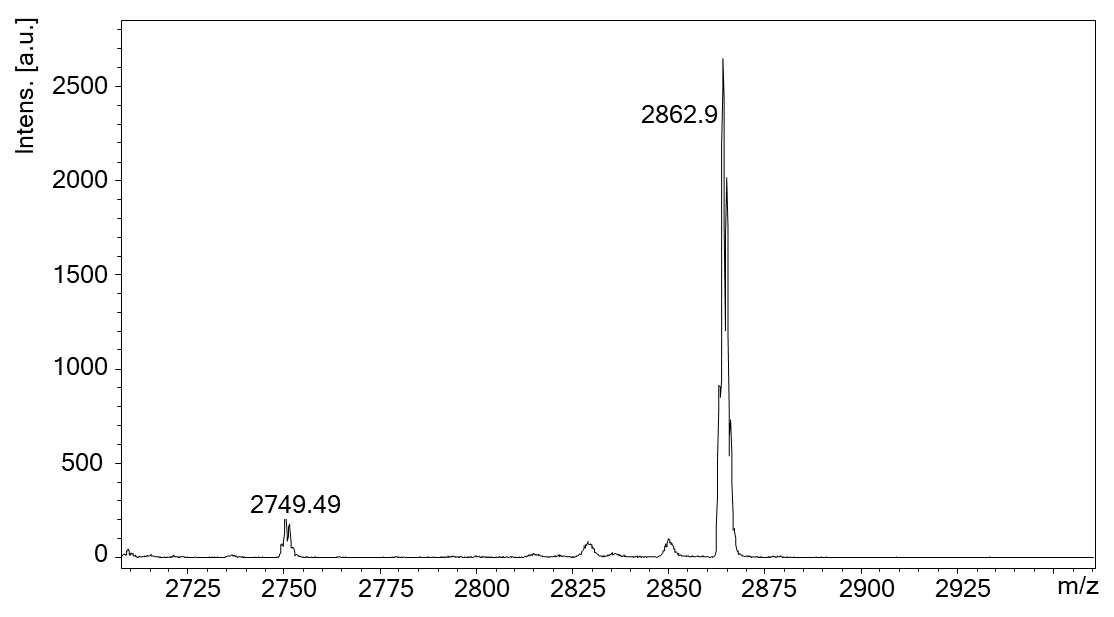


**
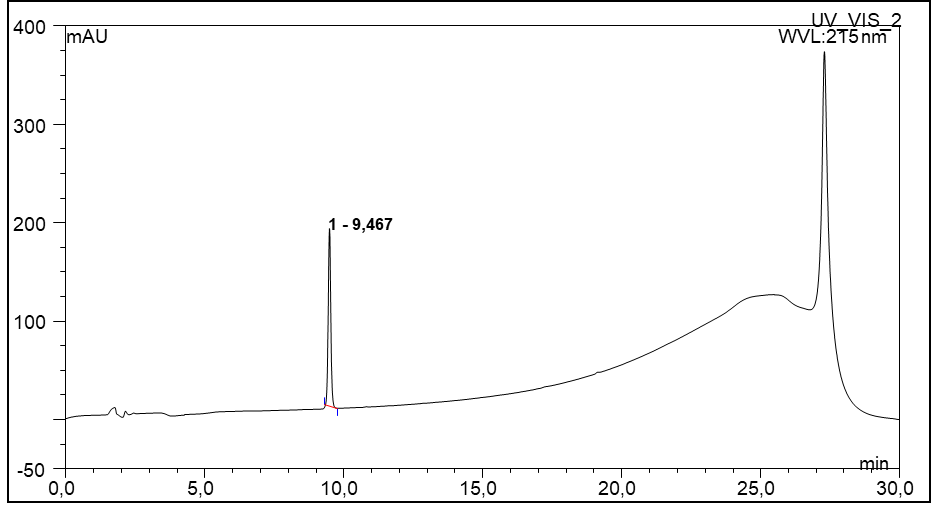
**

Figure S17 MALDI-TOF MS data for the DRG1-LysNMe peptide (*m/z observed* 2862.9) and analytical HPLC for the DRG1-LysNMe peptide after RP-HPLC purification. The peptide elutes at 9.5 min.


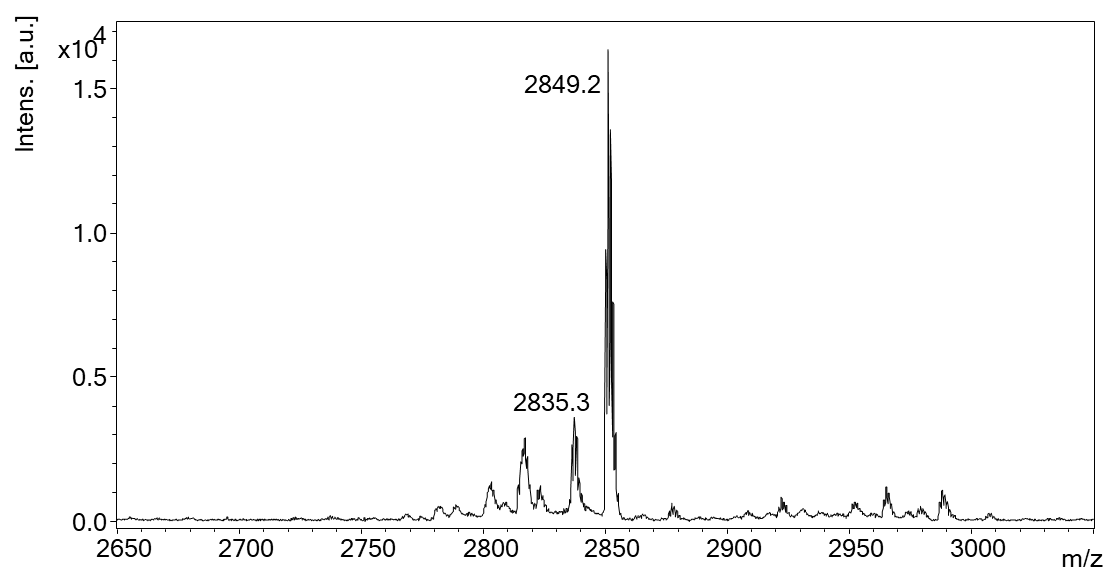


**
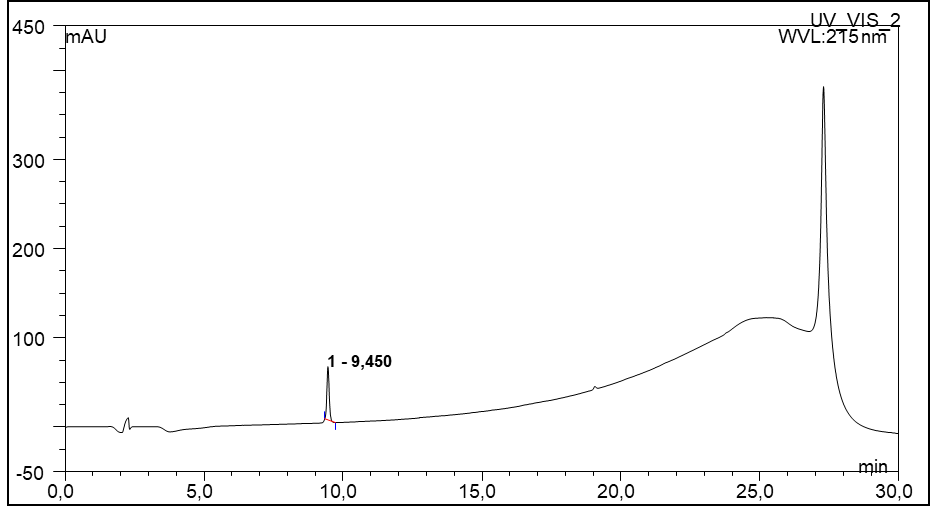
**

Figure S18 MALDI-TOF MS data for the DRG1-Abg peptide (*m/z observed* 2849.2) and analytical HPLC for the DRG1-Abg peptide after RP-HPLC purification. The peptide elutes at 9.5 min.


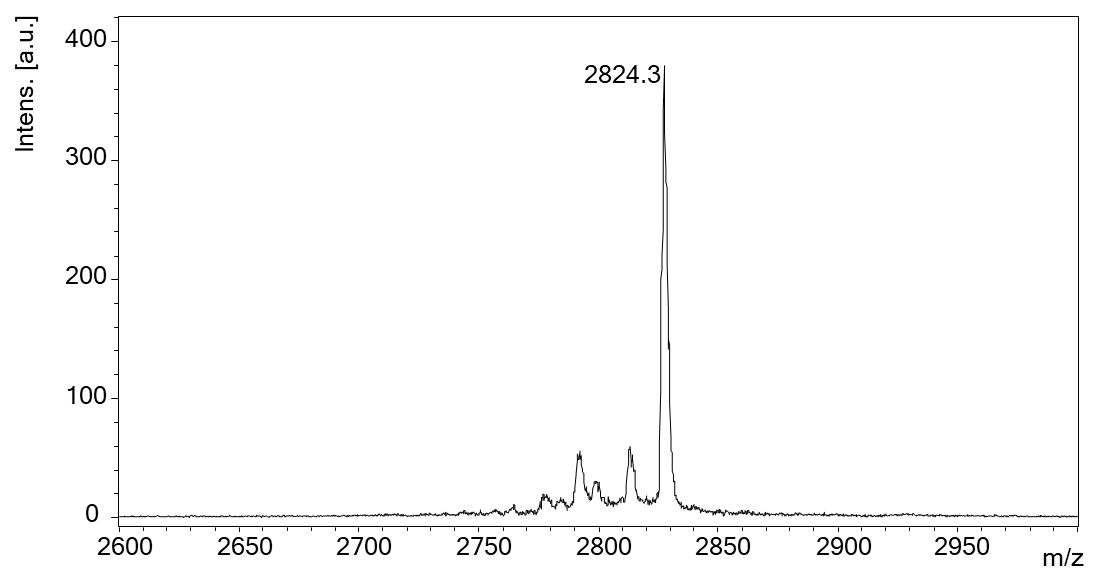


**
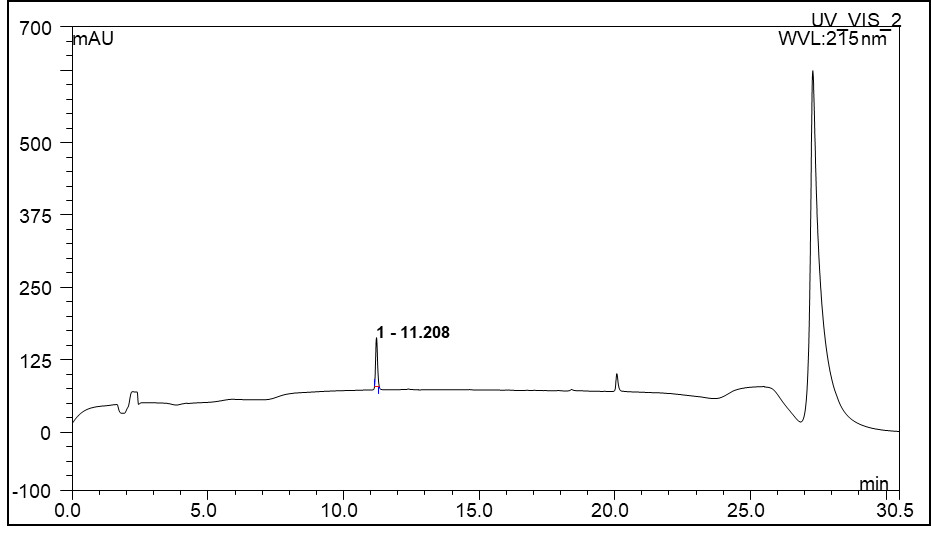
**

Figure S19 MALDI-TOF MS data for the DRG1-Cys peptide (*m/z observed* 2824.3) and analytical HPLC for the DRG1-Cys peptide after RP-HPLC purification. The peptide elutes at 11.2 min.

**
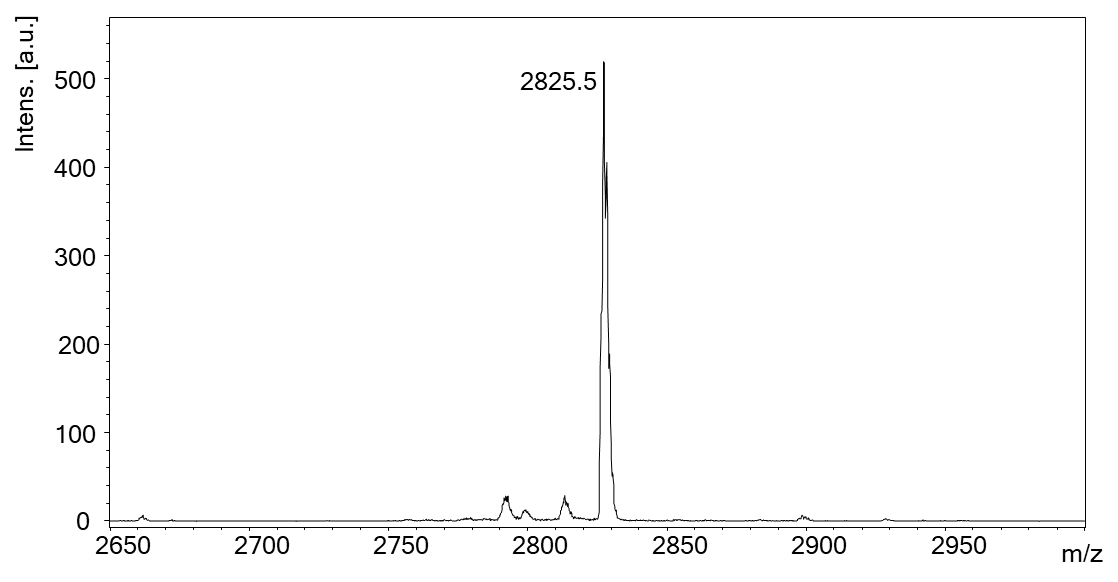
**

**
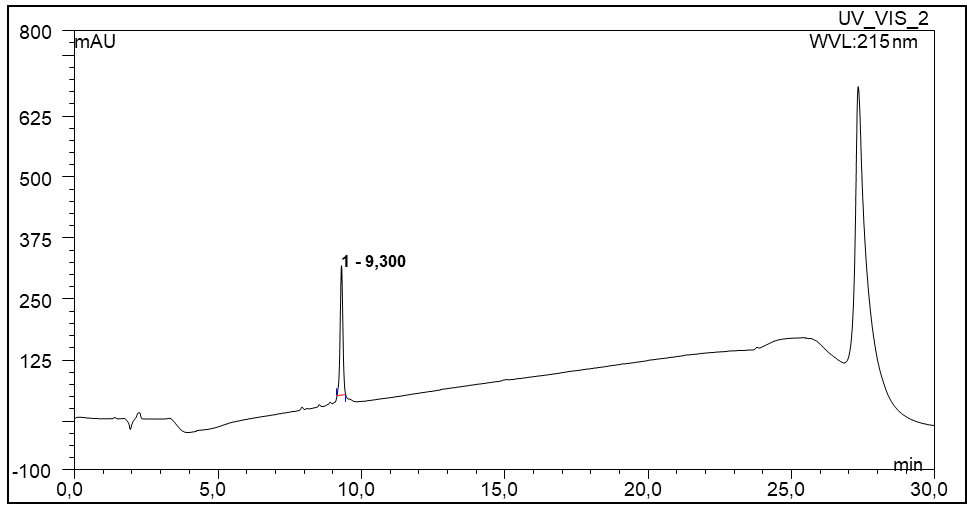
**

Figure S20 MALDI-TOF MS data for the DRG1-D-Cys peptide (*m/z observed* 2825.5) and analytical HPLC for the DRG1-D-Cys peptide after RP-HPLC purification. The peptide elutes at 9.3 min.

**
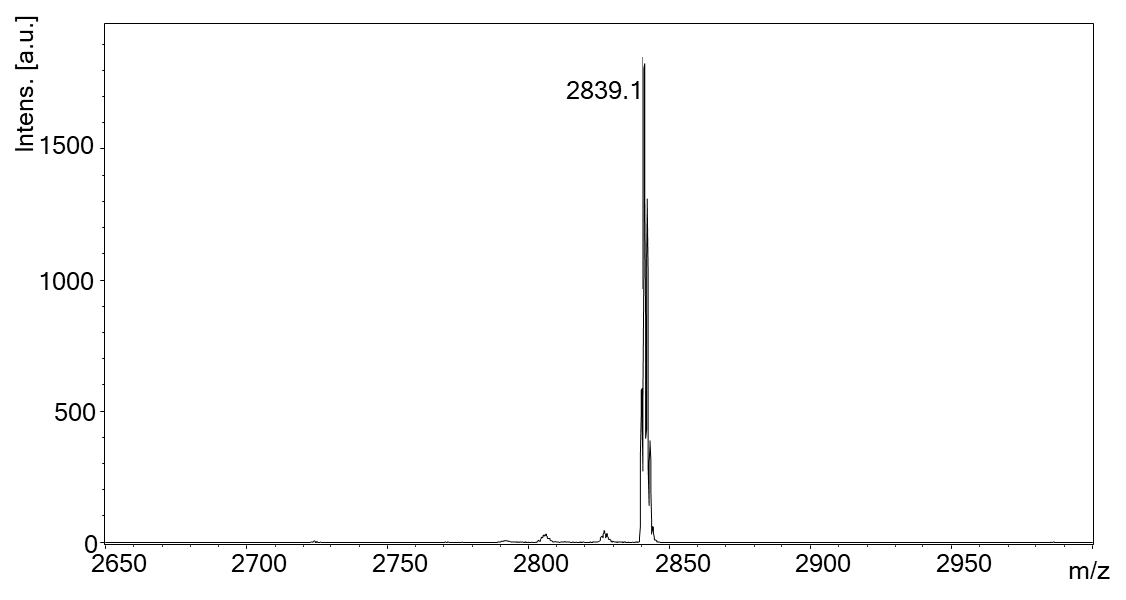
**

**
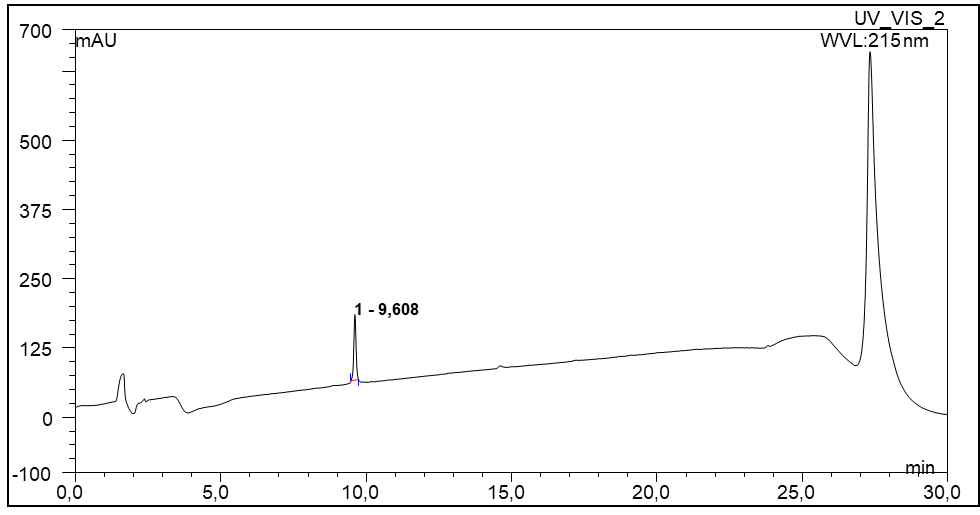
**

Figure S21 MALDI-TOF MS data for the DRG1-hCys peptide (*m/z observed* 2839.1) and analytical HPLC for the DRG1-hCys peptide after RP-HPLC purification. The peptide elutes at 9.6 min.

**
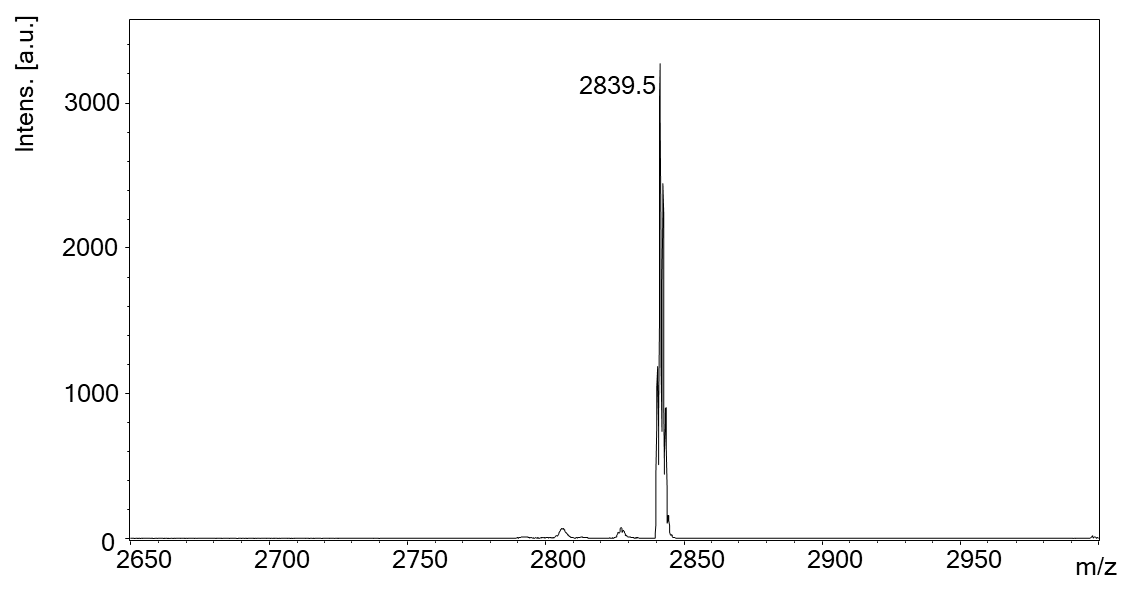
**

**
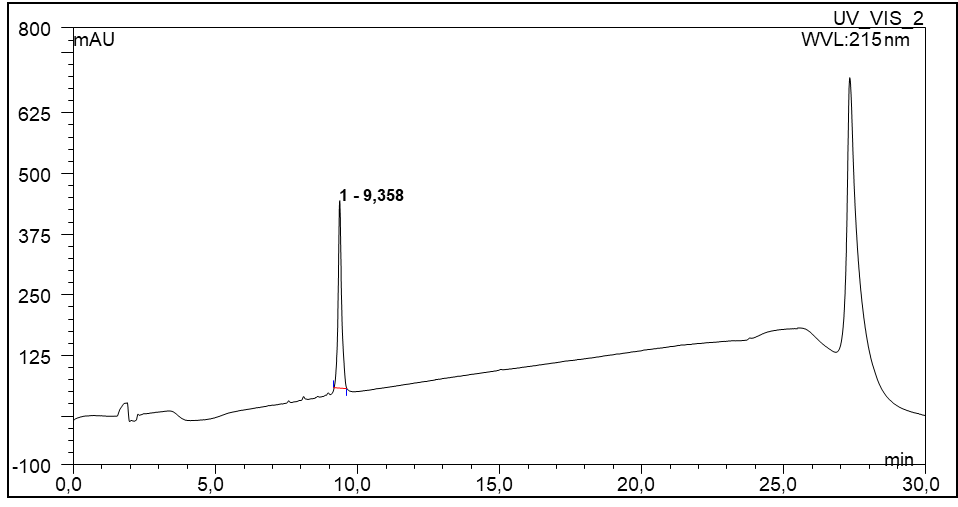
**

Figure S22 MALDI-TOF MS data for the DRG1-CysMe peptide (*m/z observed* 2839.5) and analytical HPLC for the DRG1-CysMe peptide after RP-HPLC purification. The peptide elutes at 9.4 min.


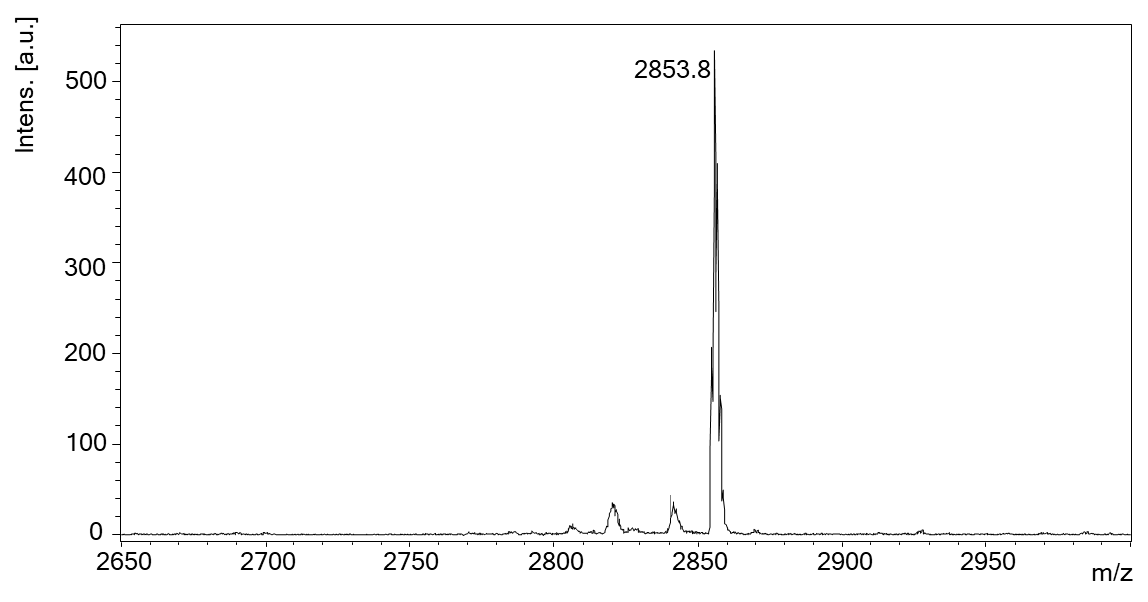


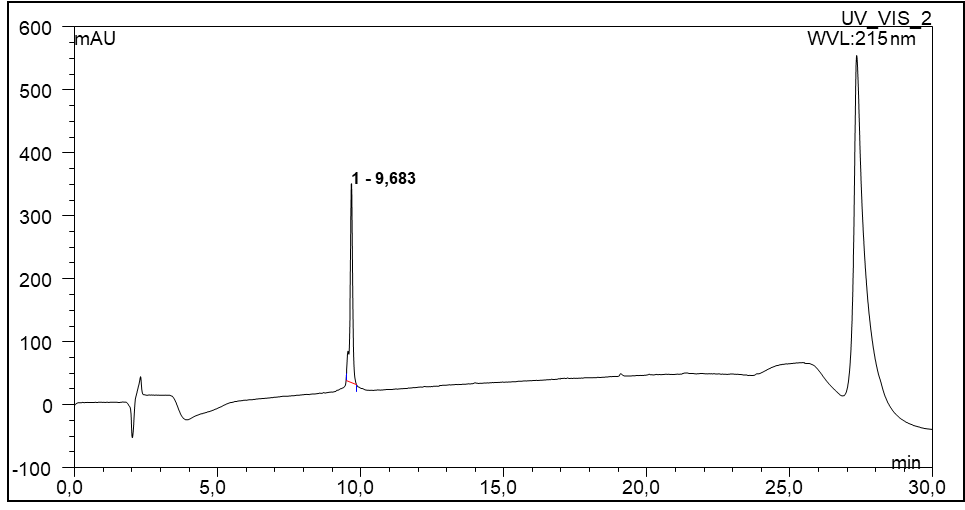


Figure S23 MALDI-TOF MS data for the DRG1-Met peptide (*m/z observed* 2853.8) and analytical HPLC for the DRG1-Met peptide after RP-HPLC purification. The peptide elutes at 9.7 min.


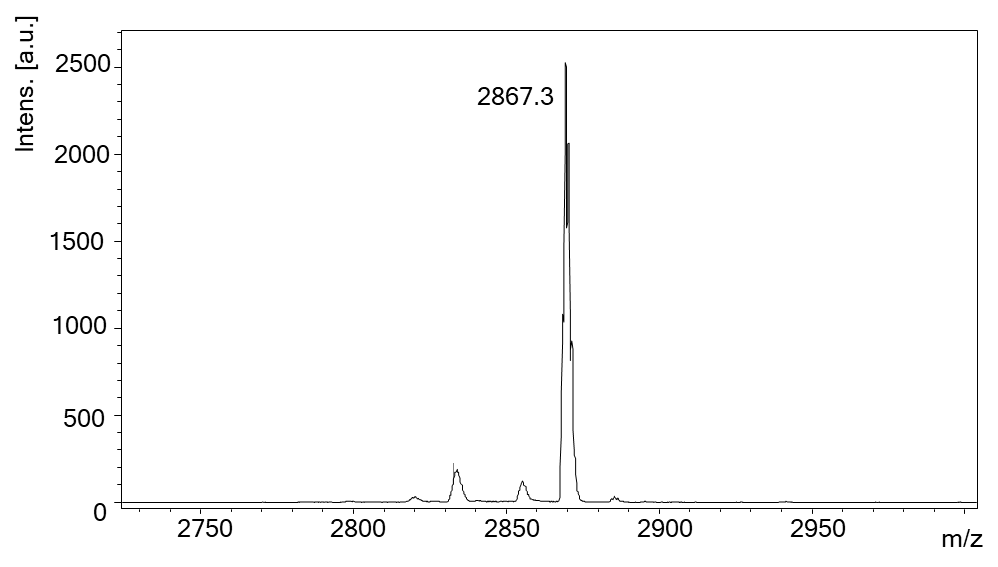


**
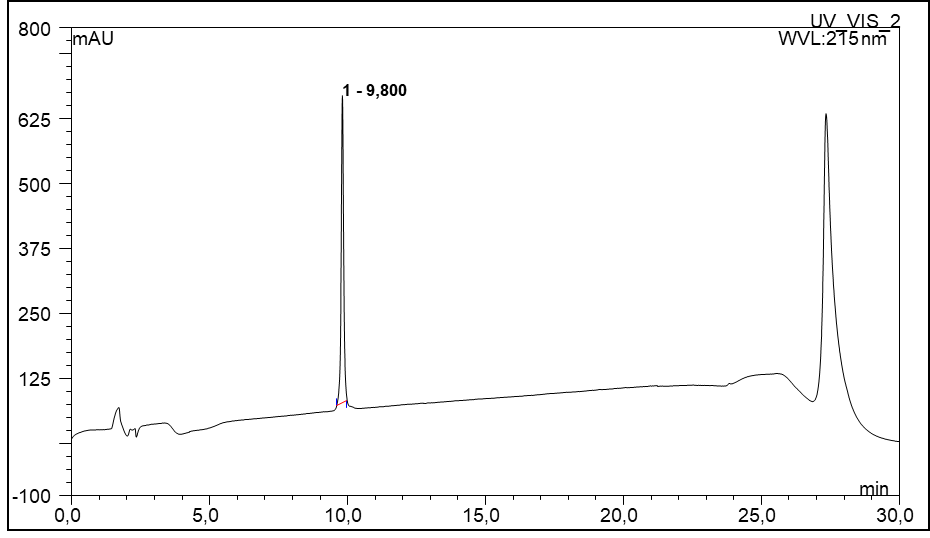
**

Figure S24 MALDI-TOF MS data for the DRG1-hMet peptide (*m/z observed* 2867.3) and analytical HPLC for the DRG1-hMet peptide after RP-HPLC purification. The peptide elutes at 9.8 min.


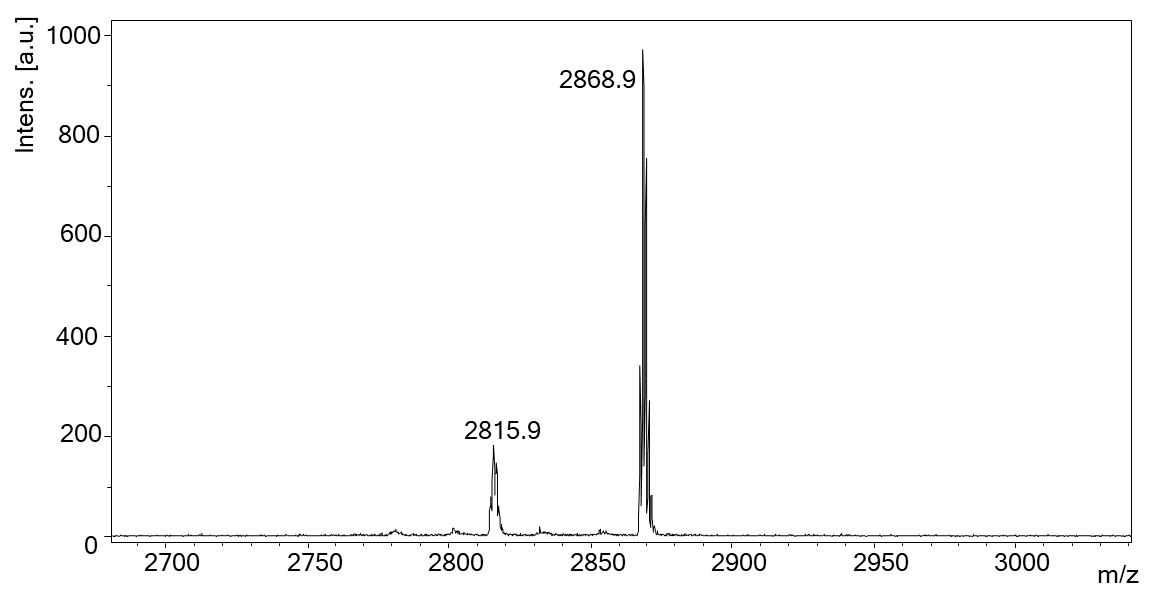


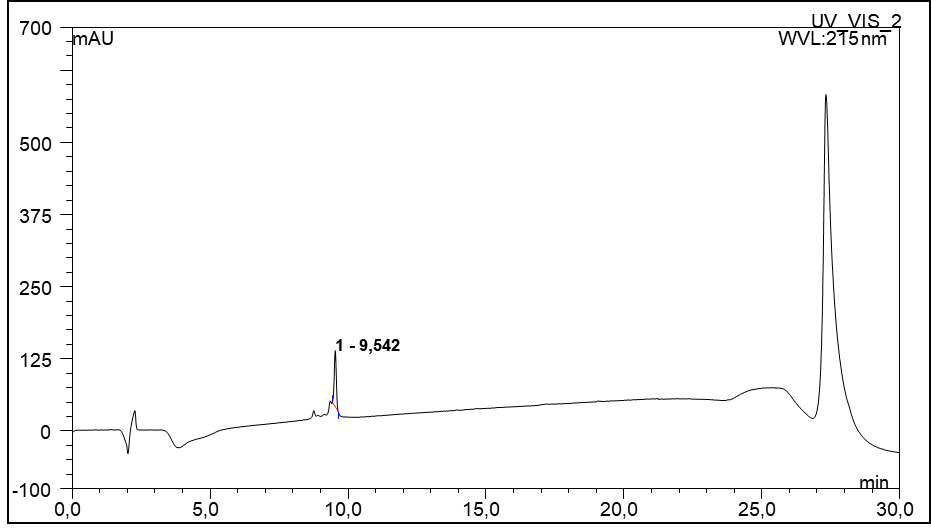


Figure S25 MALDI-TOF MS data for the DRG1-MetO peptide (*m/z observed* 2868.9) and analytical HPLC for the DRG1-MetO peptide after RP-HPLC purification. The peptide elutes at 9.5 min.

**
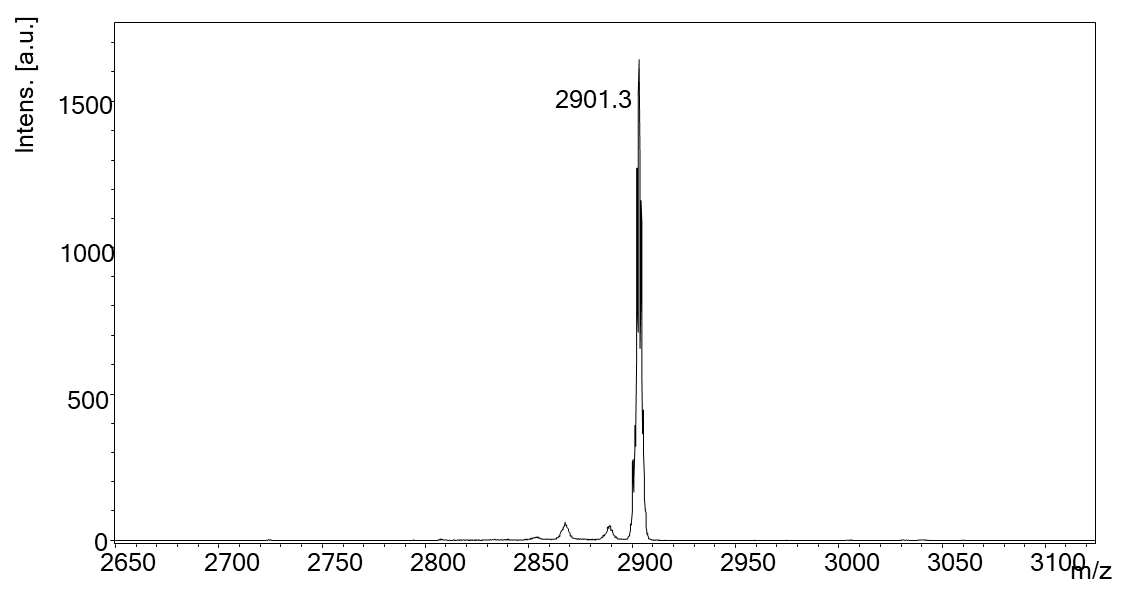
**

**
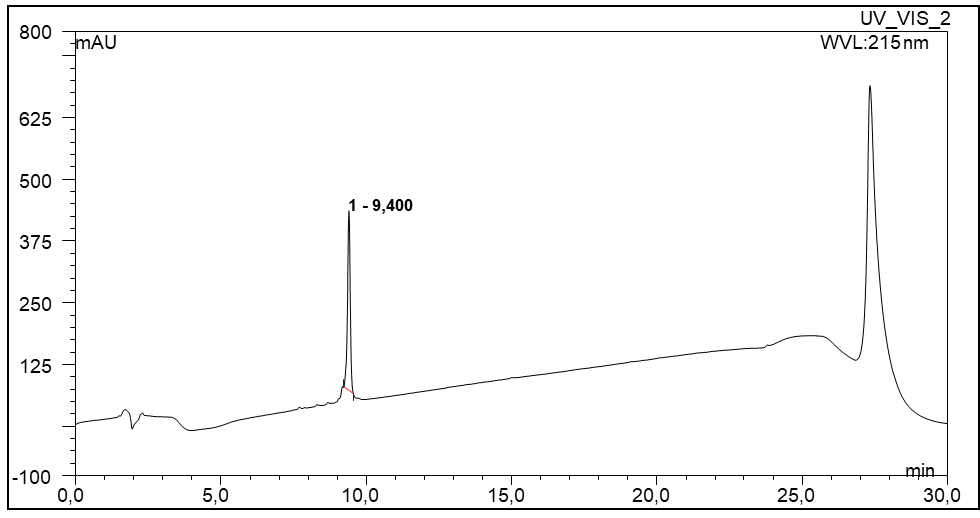
**

Figure S26 MALDI-TOF MS data for the DRG1-SeMet peptide (*m/z observed* 2901.3) and analytical HPLC for the DRG1-SeMet peptide after RP-HPLC purification. The peptide elutes at 9.4 min.

**
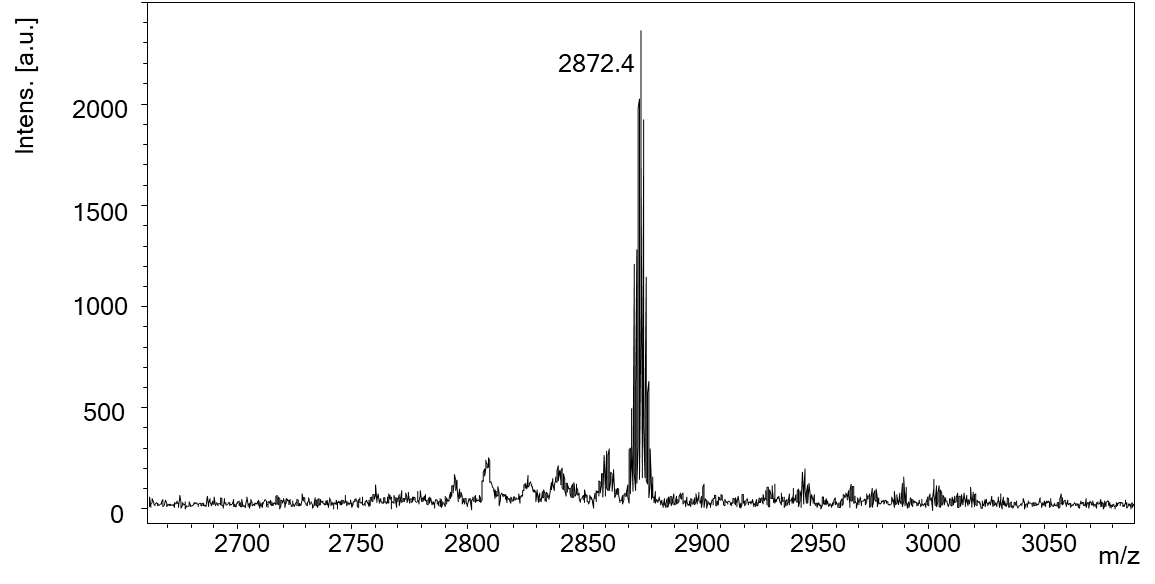
**

**
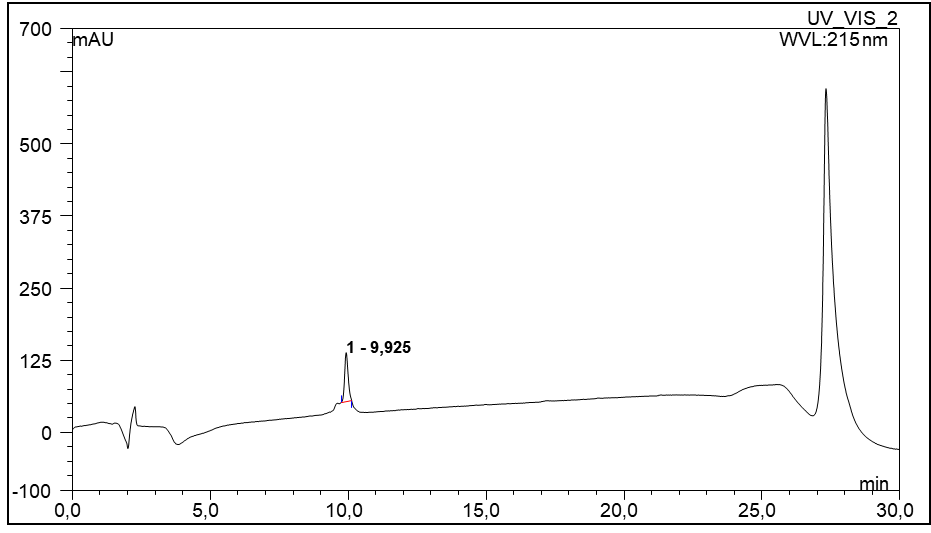
**

Figure S27 MALDI-TOF MS data for the DRG1-Sec peptide (*m/z observed* 2872.4) and analytical HPLC for the DRG1-Sec peptide after RP-HPLC purification. The peptide elutes at 9.9 min.

**
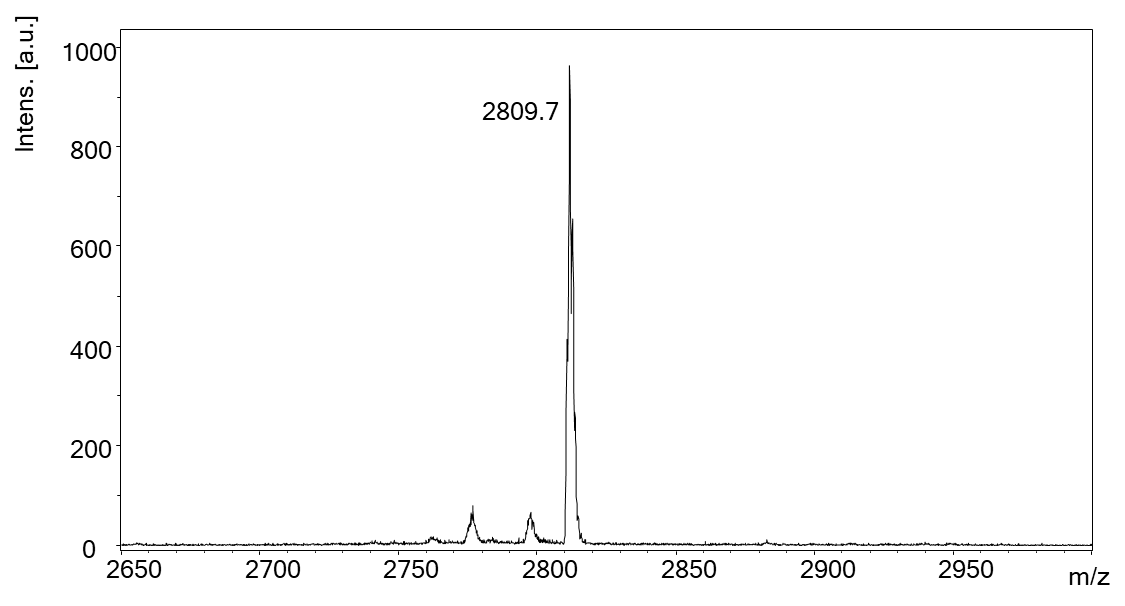
**

**
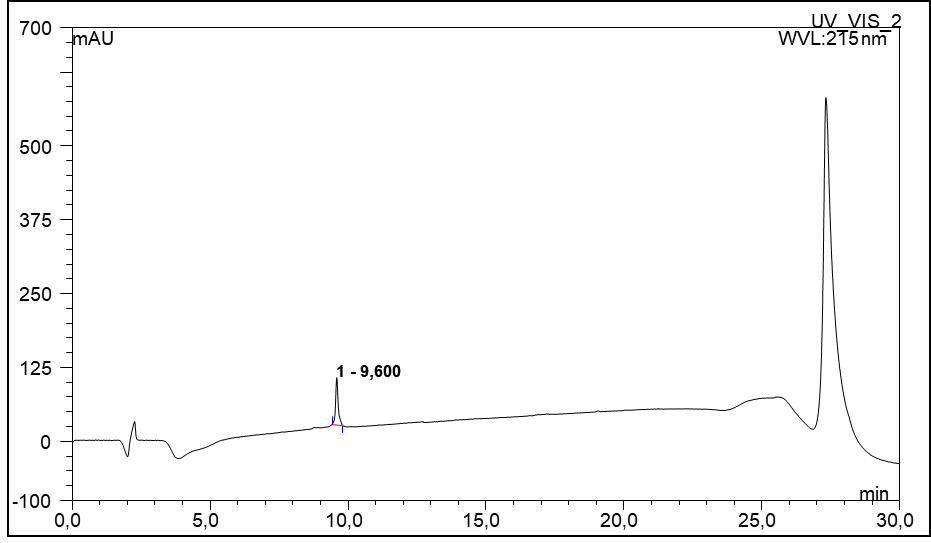
**

Figure S28 MALDI-TOF MS data for the DRG1-Ser peptide (*m/z observed* 2809.7) and analytical HPLC for the DRG1-Ser peptide after RP-HPLC purification. The peptide elutes at 9.6 min.


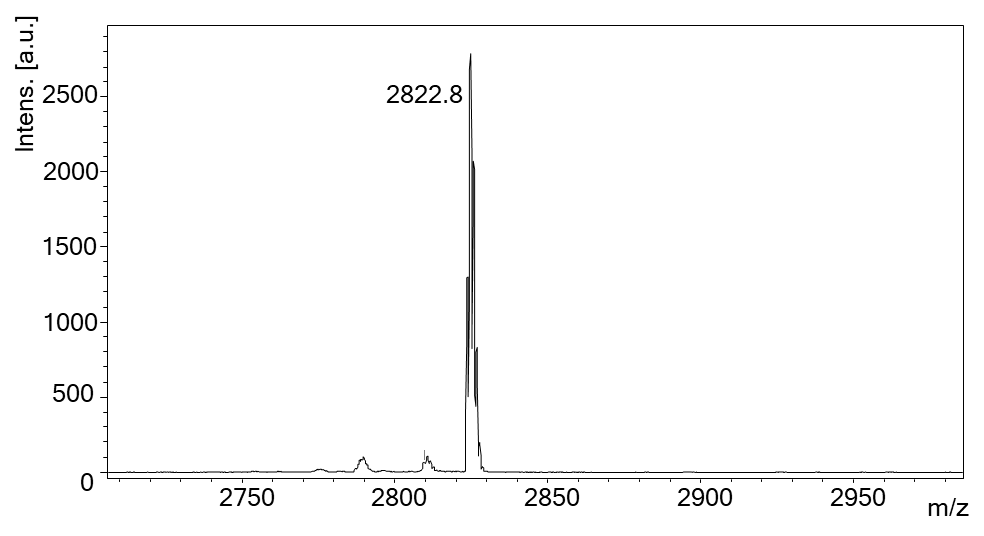


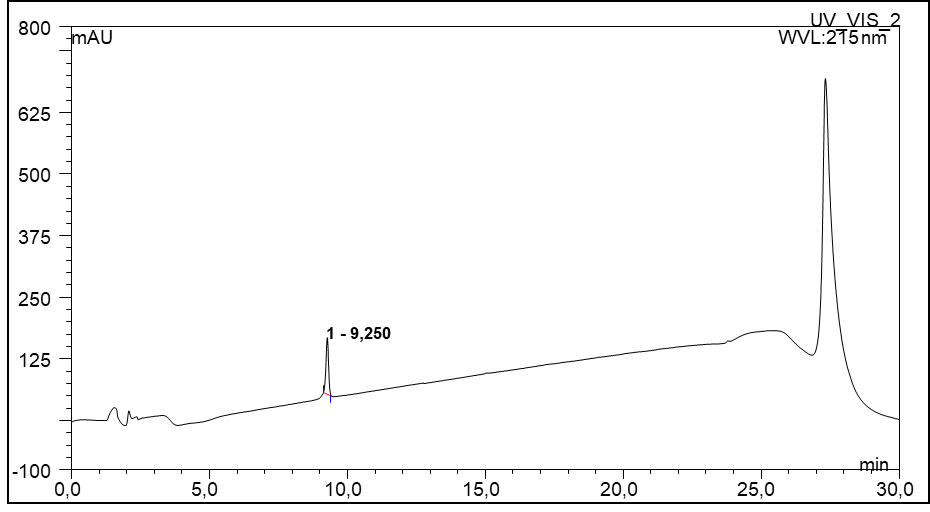


Figure S29 MALDI-TOF MS data for the DRG1-hSer peptide (*m/z observed* 2822.8) and analytical HPLC for the DRG1-hSer peptide after RP-HPLC purification. The peptide elutes at 9.3 min.

**
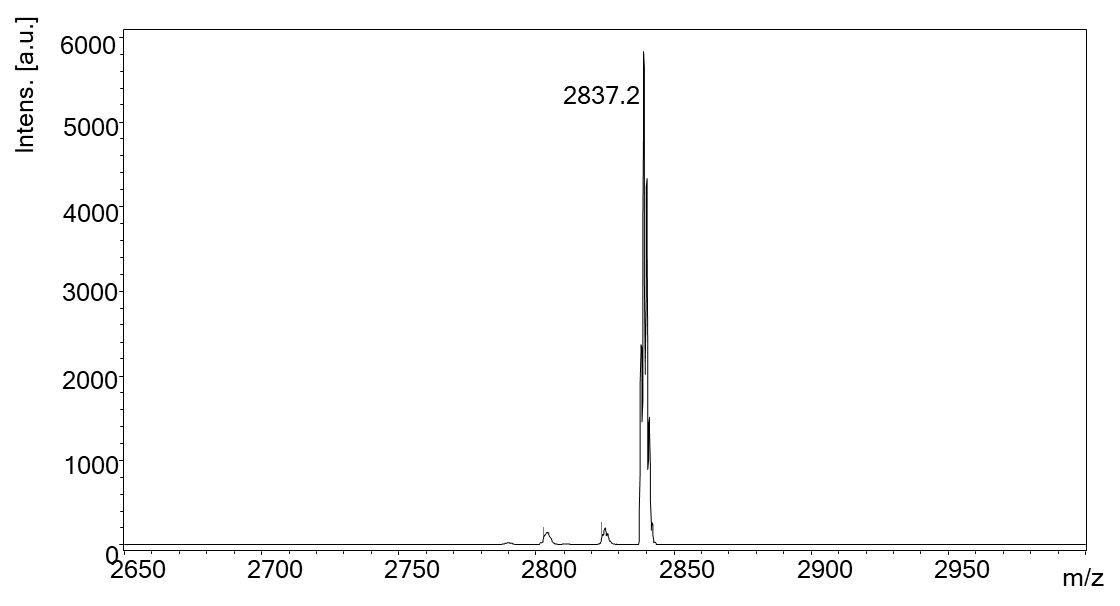
**

**
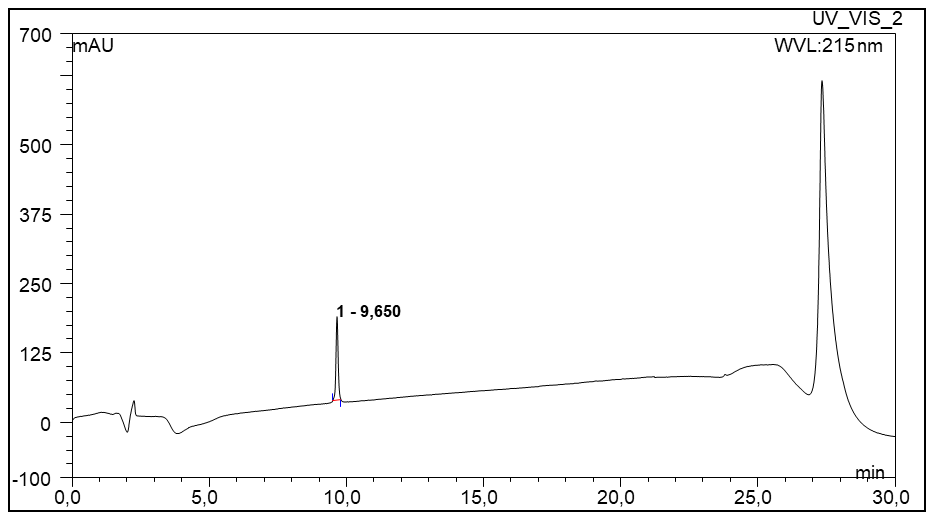
**

Figure S30 MALDI-TOF MS data for the DRG1-meOhSer peptide (*m/z observed* 2837.2) and analytical HPLC for the DRG1-meOhSer peptide after RP-HPLC purification. The peptide elutes at 9.7 min.

**2. Hydroxylation reaction of DRG1-CysNH_2_**


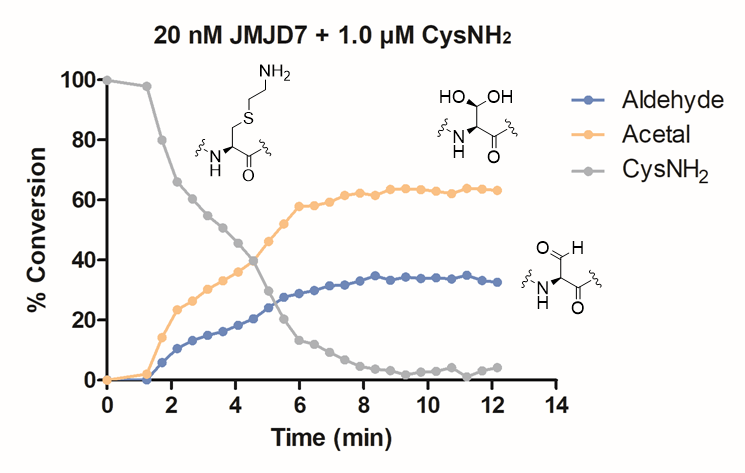


Figure S31 Time-course profile of DRG1-CysNH_2_ peptide (1.0 μM) by JMJD7-catalyzed (20 nM) hydroxylation in the presence of 2OG (20 μM), FAS (10 μM) and LAA (100 μM). Conversions of CysNH_2_ (grey; m/z 2868) to the aldehyde (blue; m/z 2807) and acetal (orange; m/z 2825) fragmented products are observed.

**3.** **LC-MS assays of lysine analogs**

**

**

Figure S32 LC-MS data showing JMJD7-catalyzed (5 μM) hydroxylation of DRG1 peptides (10 μM) in the presence of 2OG (10 μM), FAS (10 μM) and LAA (100 μM). a) DRG1-Lys, b) DRG1-D-Lys, c) DRG1-Orn, d) DRG1-hLys, e) DRG1-LysMe, f) DRG1-HNle, g) DRG1-Ahp, h) DRG1-CysNH_2_, i) DRG1-CysNHMe, j) DRG1-LysN, k) DRG1-LysE, l) DRG1-nArg, m) DRG1-hGln, n) DRG1-4pyrA, o) DRG1-βhLys, p) DRG1-LysCMe, q) DRG1-LysNMe, and r) DRG1-Abg.

**

**

Figure S33 LC-MS data showing JMJD7-catalyzed (2 μM) hydroxylation of DRG1 peptides (10 μM) in the presence of 2OG (20 μM), FAS (10 μM) and LAA (100 μM). a) DRG1-Lys, b) DRG1-D-Lys, c) DRG1-Orn, d) DRG1-hLys, e) DRG1-LysMe, f) DRG1-HNle, g) DRG1-Ahp, h) DRG1-CysNH_2_, i) DRG1-CysNHme, j) DRG1-LysN, k) DRG1-LysE, l) DRG1-nArg, m) DRG1-hGln, n) DRG1-4pyrA, o) DRG1-βhLys, p) DRG1-LysCme, q) DRG1-LysNme, and r) DRG1-Abg.

**4. NMR supporting figures**


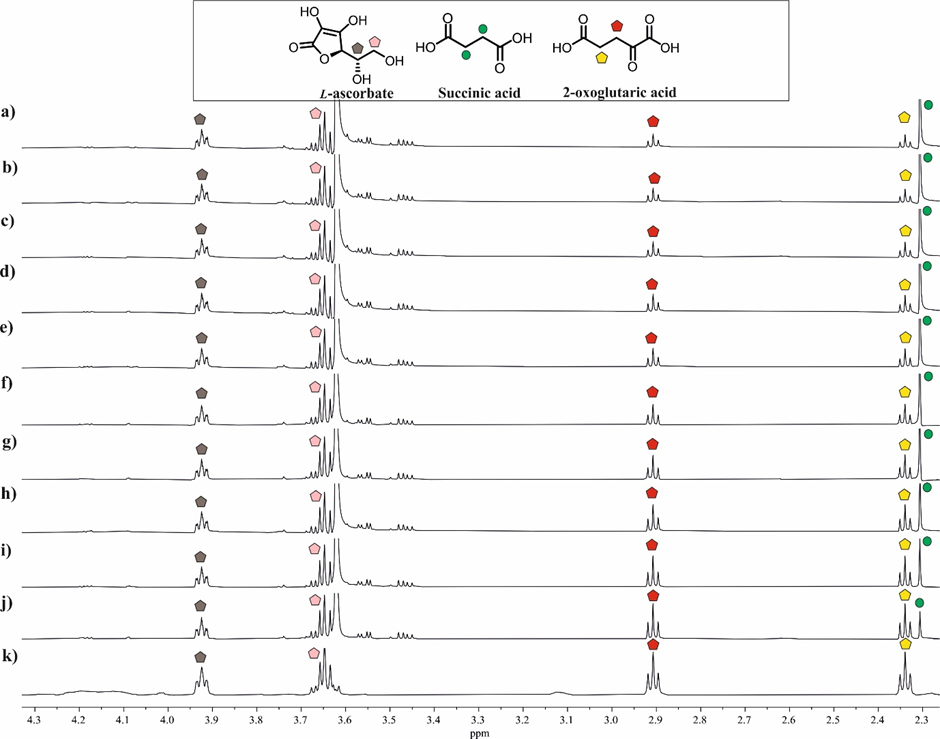


Figure S34 ^1^H NMR time-course analysis for the reaction of the DRG1-Lys peptide as a JMJD7 substrate. Conditions: 500 μM L-ascorbate, 200 μM 2OG, 200 μM peptide, 30 μM FAS, 10 μM JMJD7 in 50 mM sodium phosphate buffer (10% v/v D_2_O, pH 7.4). a)-j) NMR profiles after, a) 150 min, b) 135 min, c) 120 min, d) 105 min, e) 90 min, f) 75 min, g) 60 min, h) 45 min, i) 30 min, j) 15 min. k) no-enzyme control with the DRG1-Lys substrate under the reaction conditions. Note the conversion of 2OG to succinate.


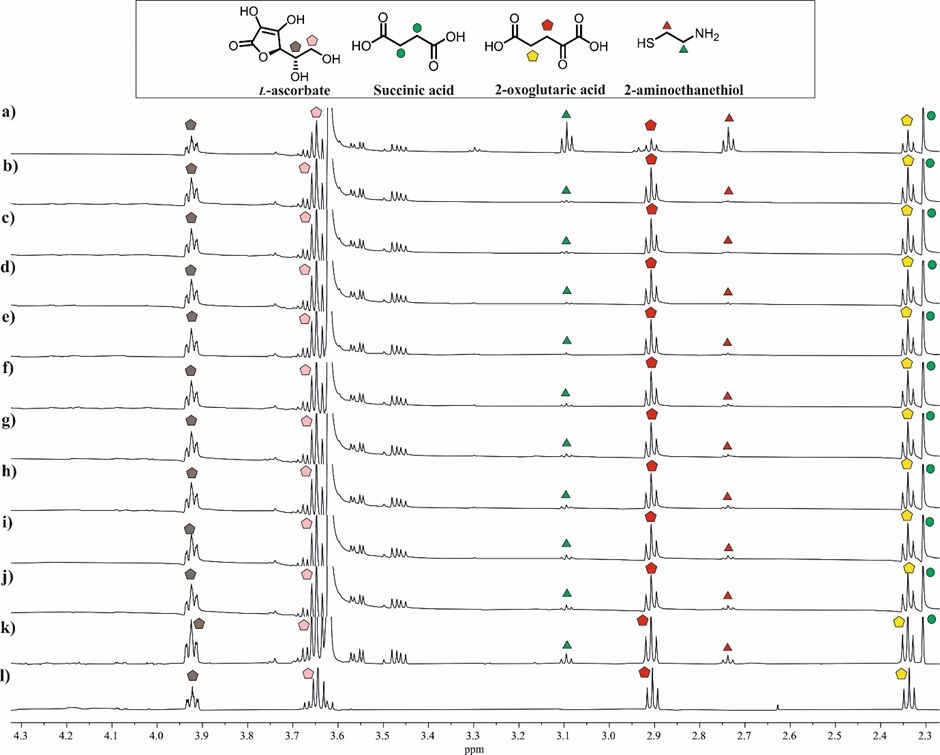


Figure S35 ^1^H NMR time-course analysis for the reaction of the DRG1-CysNH_2_ peptide as a JMJD7 substrate. Conditions: 500 μM L-ascorbate, 200 μM 2OG, 200 μM peptide, 30 μM FAS, 10 uM JMJD7 in 50 mM sodium phosphate buffer (10% v/v D_2_O, pH 7.4). a) The reaction mixture at 150 min was spiked with an authentic sample of 2-aminoethanethiol (cysteamine), b-j) NMR profiles after, b) 150 min c) 135 min, d) 120 min, e) 105 min, f) 90 min, g) 75 min, h) 60 min, i) 45 min, j) 30 min, k) 15 min. l) no-enzyme control with the DRG1-CysNH_2_ substrate under the reaction conditions. Note the conversion of 2OG to succinate and formation of 2-aminoethanethiol (cysteamine).


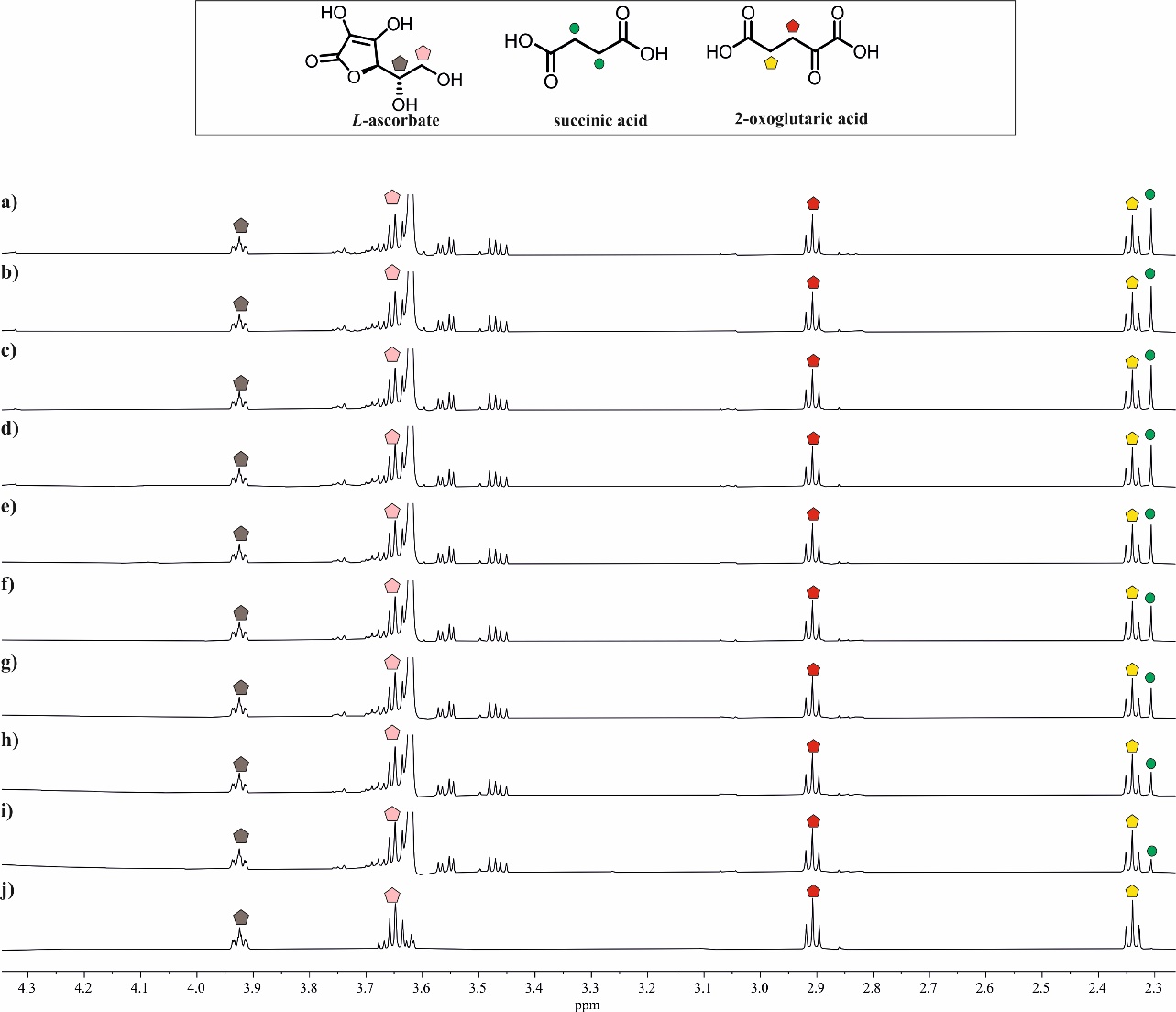


Figure S36 ^1^H NMR time-course analysis for the reaction of the DRG1-LysE peptide as a JMJD7 substrate. **a)-j)** Reaction times of 500 μM L-ascorbate, 200 μM 2-OG, 200 μM DRG1-LysE peptide, 30 μM Fe(II), 10 μM JMJD7 in 50 mM sodium phosphate buffer (10% v/v D_2_O, pH 7.4), a) 150 min, b) 120 min, c) 105 min, d) 90 min, e) 75min, f) 60 min, g) 45 min, h) 30 min, i) 15 min. **j)** 500 uM L-sscorbate, 200 μM 2-OG, 200 μM DRG1-LysE peptide, 30 μM Fe(II), 0 μM JMJD7 in 50 mM sodium phosphate buffer (10% v/v D_2_O, pH 7.4). Note the conversion of 2OG to succinate.

**5. Supporting LC-MS assay data**

**
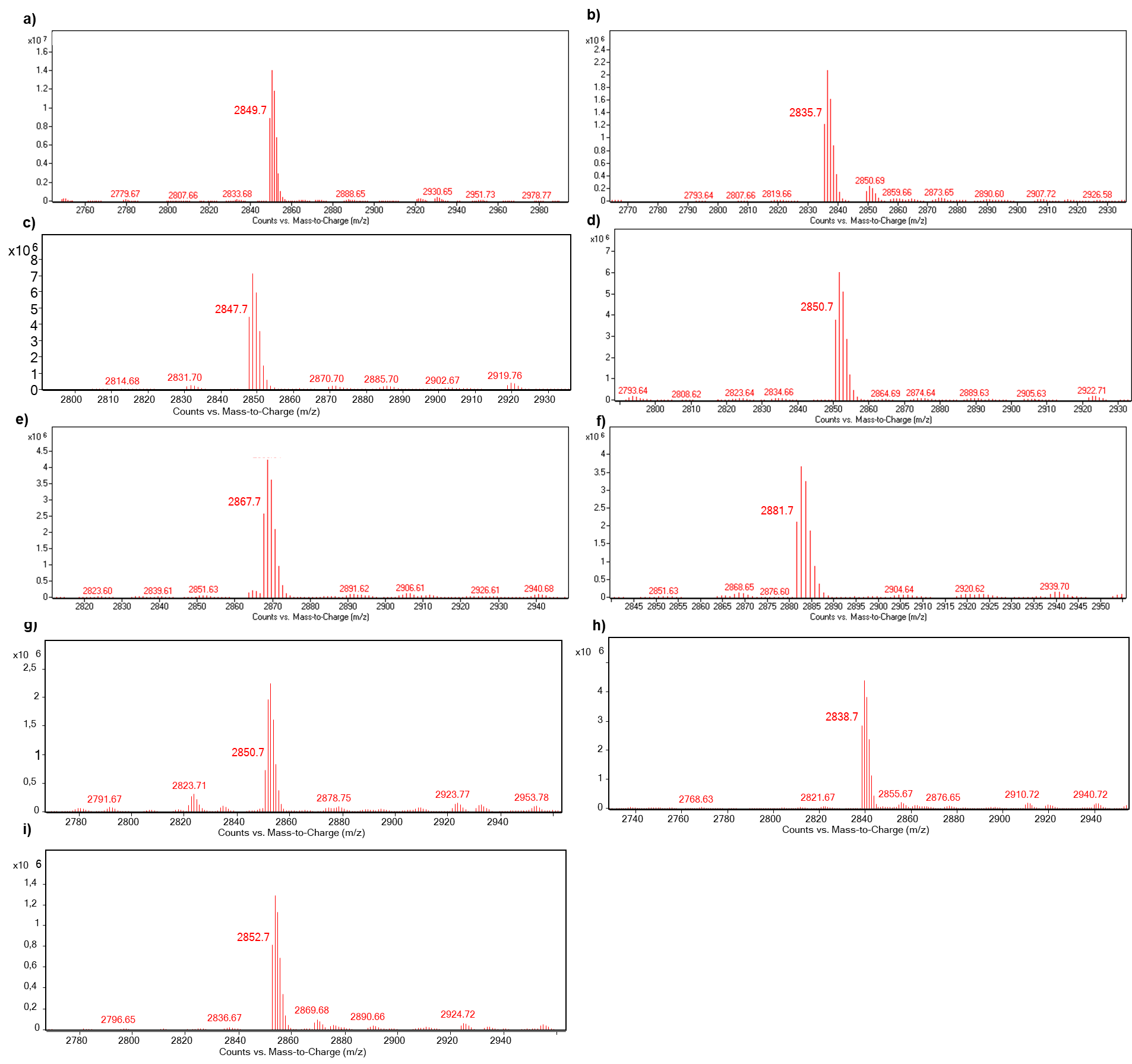
**

Figure S37 LC-MS data showing no-enzyme controls for the tested DRG1 substrate peptides (10 μM) in the presence of 2OG (20 μM), FAS (10 μM) and LAA (100 μM). a) DRG1-Lys, b) DRG1-Orn, c) DRG1-LysE, d) DRG1-HNle, e) DRG1-CysNH_2_, f) DRG1-CysNHme, g) DRG1-LysN, h) DRG1-CysMe, and i) DRG1-Met.


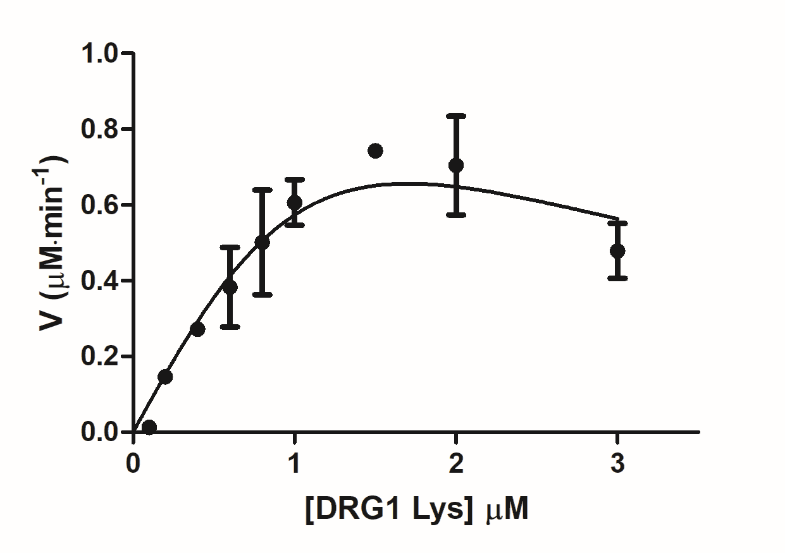


Figure S38 Plot for hydroxylation of the DRG1-Lys substrate showing evidence for substrate inhibition (100 nM JMJD7, 20 μM 2OG, 10 μM FAS, 100 μM LAA, pH 7.5).


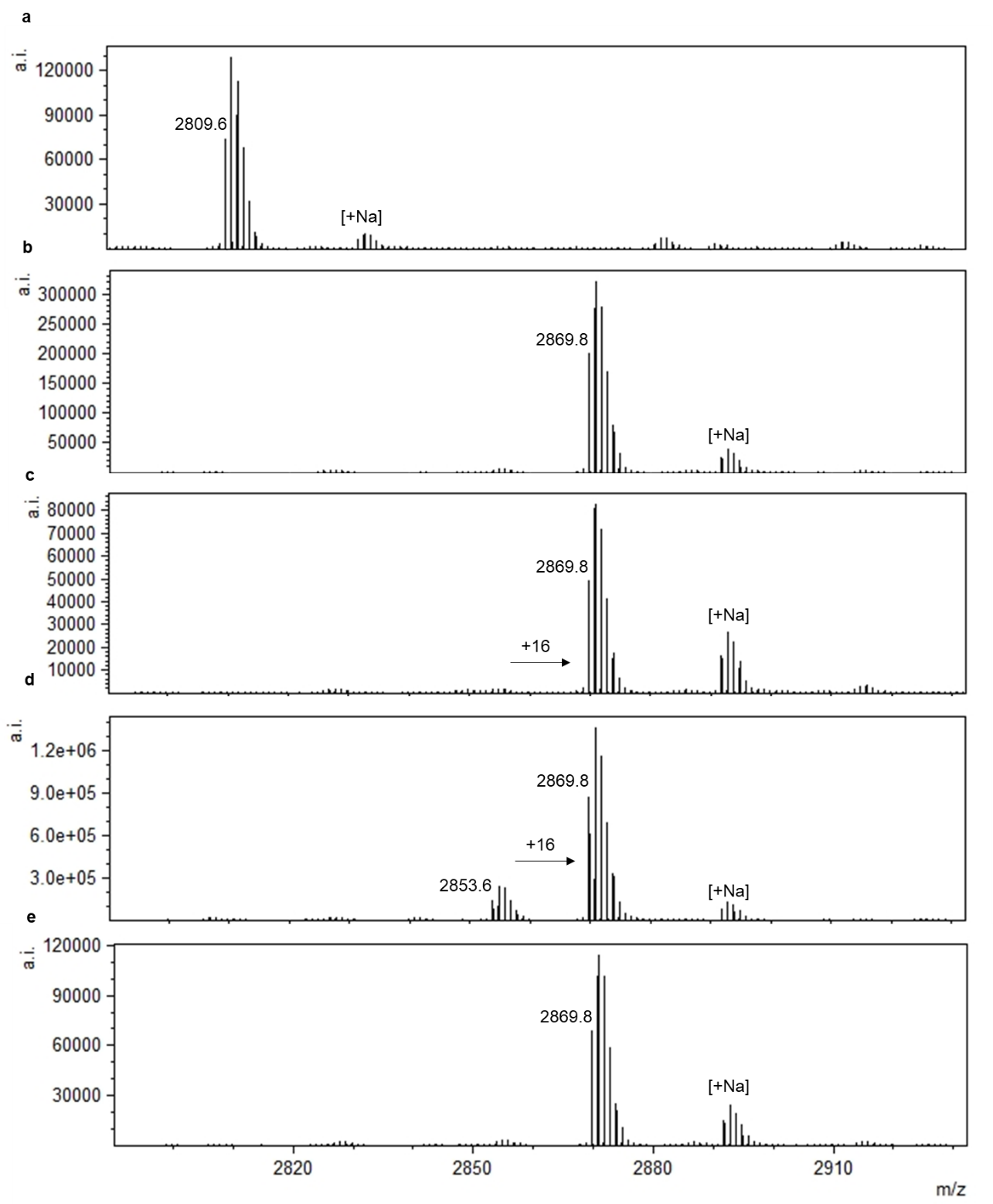


Figure S39 Chemoselective oxidation reactions of DRG1 peptides possessing Ser, MetO and Met with Selectflour measured by MALDI-TIMS-TOF MS: a) DRG1-Ser (10 μM), b) DRG1-MetO (10 μM), and c) DRG1-Met in the presence of 20 μM 2OG, 20 μM FAS, 50 μM LAA after 1 h reaction with Selectfluor (100 μM) and pyridine (50 μM) in 10 mM phosphate buffer at pH 7.0, d) JMJD7-catalyzed (2 μM) oxidation reaction of DRG1-Met (10 μM) peptide in the presence of 20 μM 2OG, 20 μM FAS, 50 μM LAA after 2 h, and e) JMJD7-catalyzed DRG1-Met peptide sample after 1 h reaction with Selectfluor (100 μM) and pyridine (50 μM) in 10 mM phosphate buffer at pH 7.0.


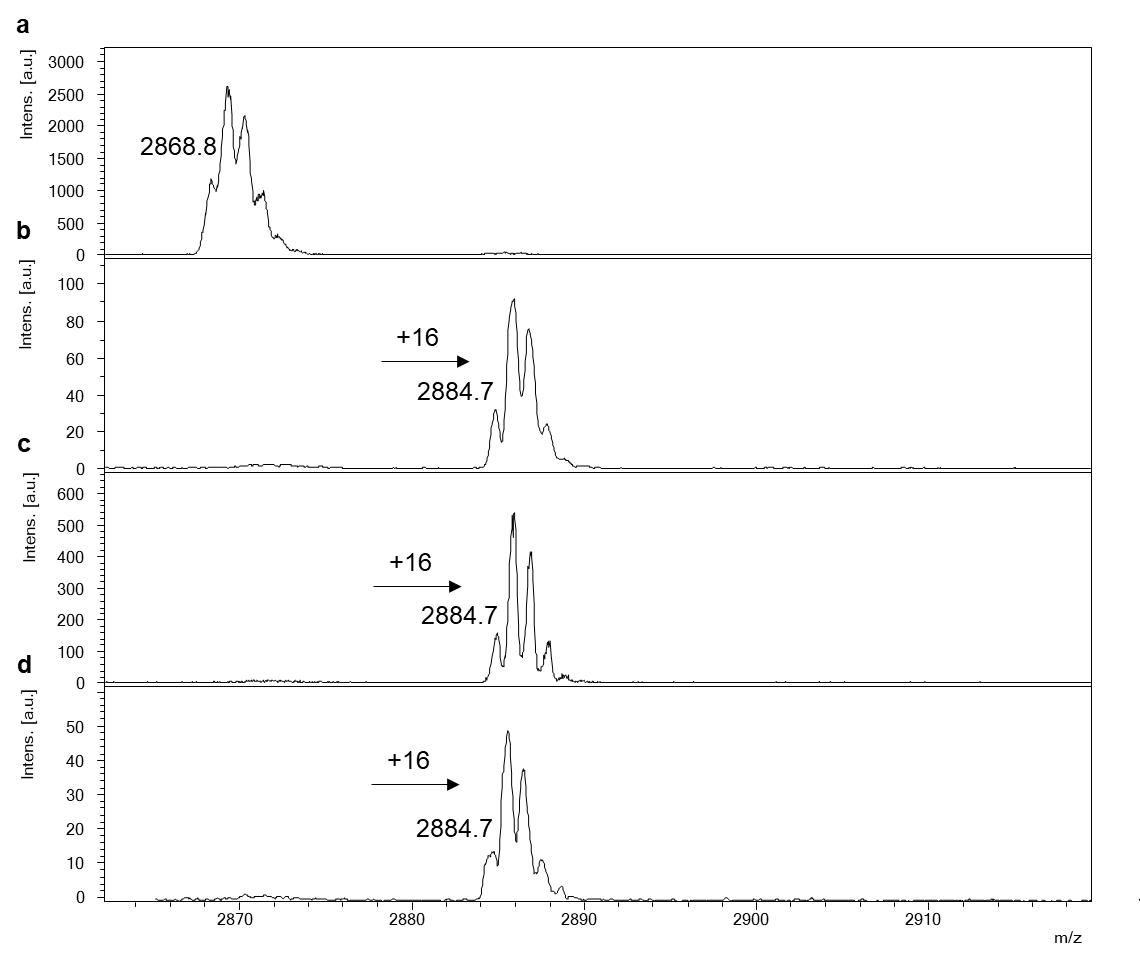


Figure S40 Chemoselective oxidation reactions of DRG1 peptides possessing hMet with Selectflour measured by MALDI-TOF MS: a) DRG1-hMet (10 μM) in the presence of 20 μM 2OG, 20 μM FAS, 50 μM LAA, b) DRG1-hMet after in the presence of 20 μM 2OG, 20 μM FAS, 50 μM LAA after 1 h reaction with Selectfluor (100 μM) and pyridine (50 μM) in 10 mM phosphate buffer at pH 7.0, c) JMJD7-catalyzed (2 μM) oxidation reaction of DRG1-hMet (10 μM) peptide in the presence of 20 μM 2OG, 20 μM FAS, 50 μM LAA after 2 h, and d) JMJD7-catalyzed DRG1-hMet peptide sample after 1 h reaction with Selectfluor (100 μM) and pyridine (50 μM) in 10 mM phosphate buffer at pH 7.0.


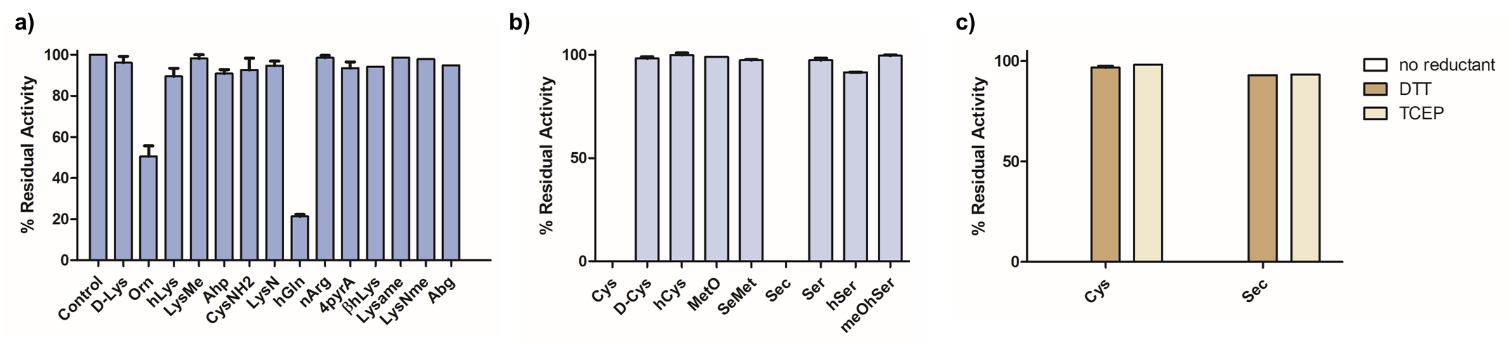


Figure S41 Single point inhibition data of human JMJD7 (200 nM) by: a) DRG1-Lys, b) DRG1-Cys analog peptides (10 μM) in the presence of the DRG1 substrate (5 μM), and c) Single point inhibition data of human JMJD7 (200 nM) by DRG1-Cys or DRG1-Sec peptides (50 μM) in the presence of DRG1-Lys substrate (5 μM), in the presence and absence of a reductant in reaction buffer (DTT or TCEP). Residue structures are defined in Figure 2/Figure 6 in the main text.


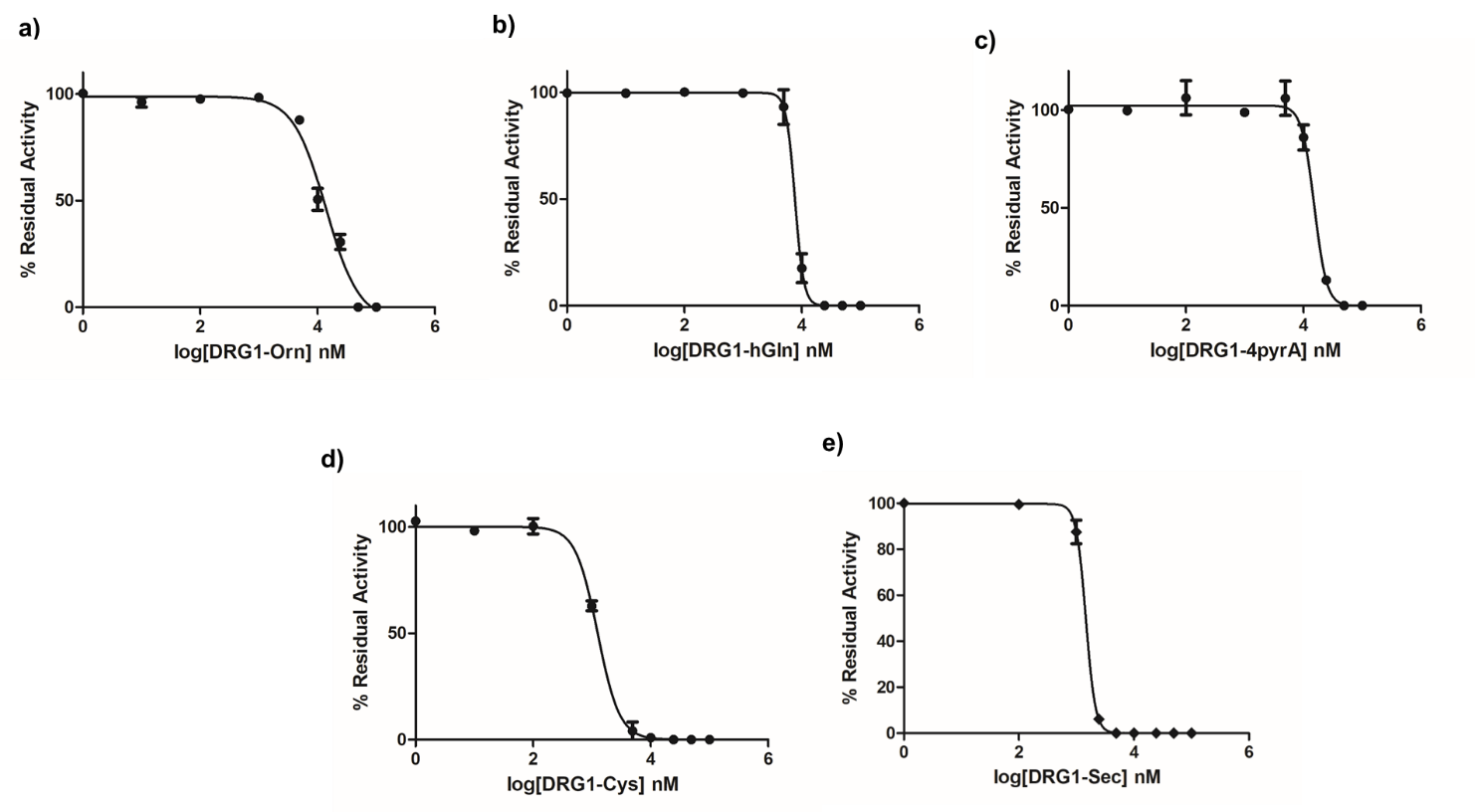


Figure S42 JMJD7 inhibition dose-response curves for: a) DRG1-Orn, b) DRG1-hGln, c) DRG1-4pyrA, d) DRG1-Cys, and e) DRG1-Sec, showing inhibition of JMJD7. Error bars reported as SEM (n = 2).


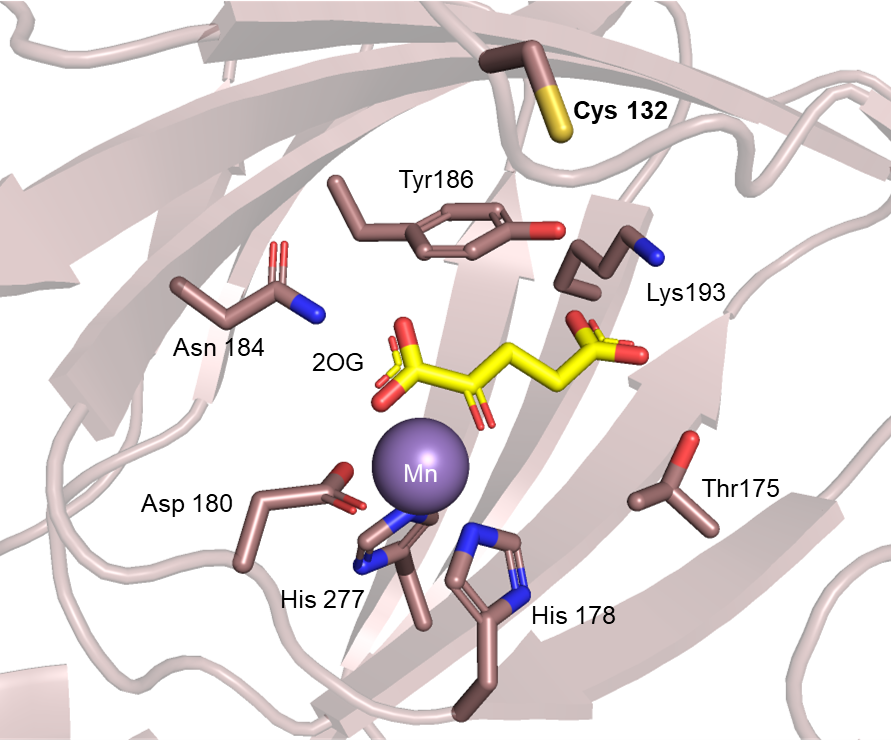


Figure S43 Close-up view of the active-site of JMJD7 in complex with 2OG (yellow) and Mn(II) (substituting for catalytically active Fe(II), purple) (PDB: 5NFN). Note the proximity of Cys132 to the active site.


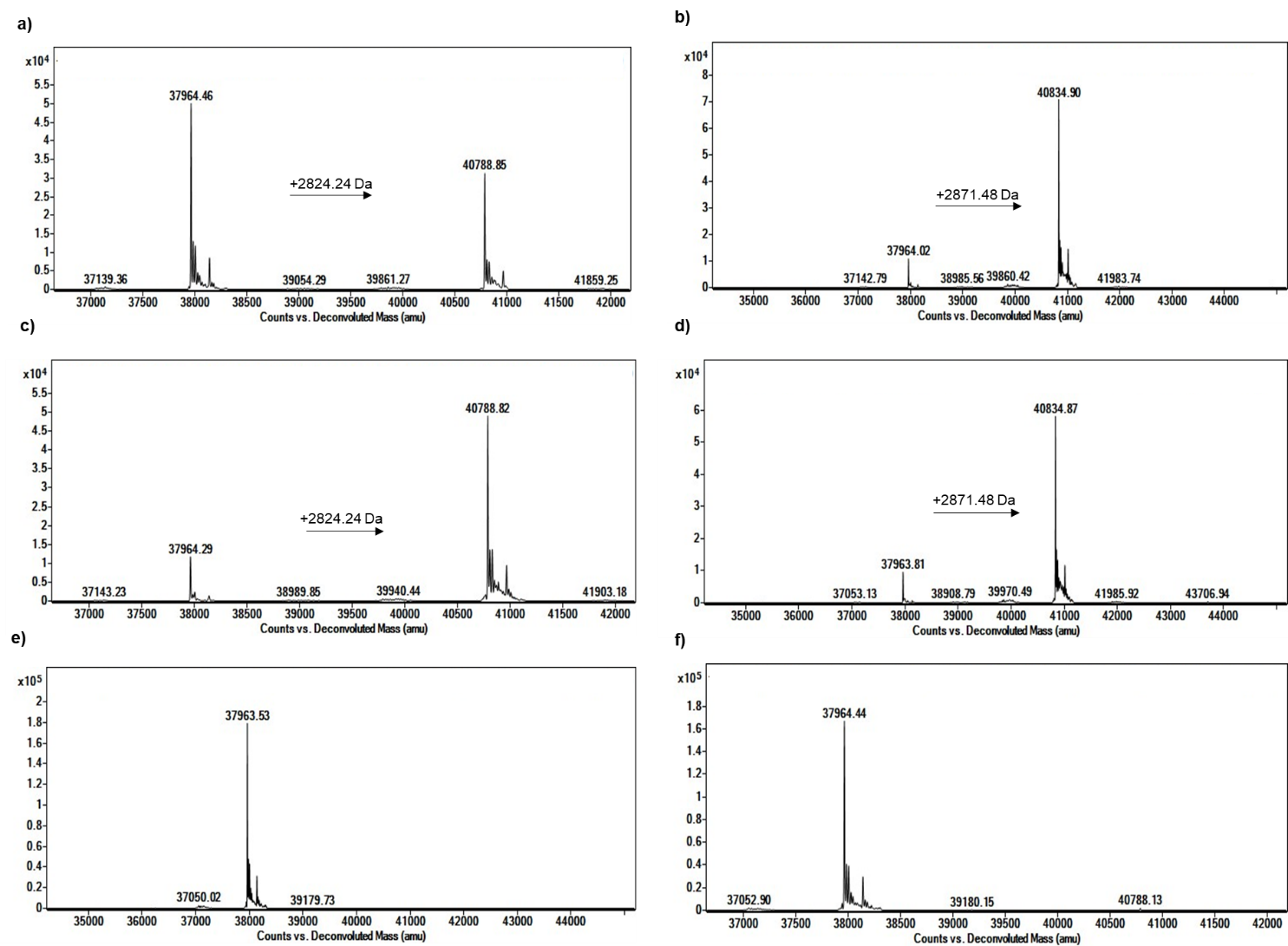


Figure S44 Q-TOF-MS data of intact JMJD7 (2 μM) after a) 1 min incubation with DRG1-Cys (20 μM), b) 1 min incubation with DRG1-Sec (20 μM), c) 30 min incubation with DRG1-Cys (20 μM) d) 30 min incubation with DRG1-Sec (20 μM), e) incubation with DRG1-Sec (20 μM) in the presence of DTT in the reaction buffer, and f) incubation with DRG1-Cys (20 μM) in the presence of DTT in the reaction buffer.


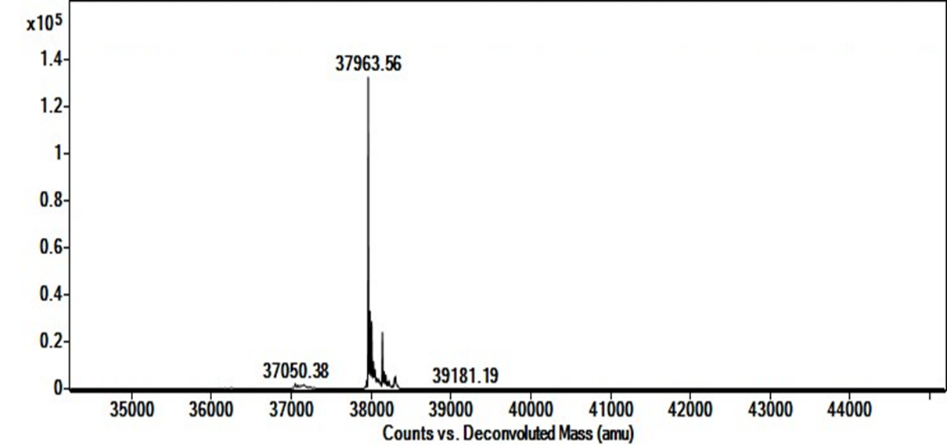


Figure S45 Q-TOF-MS data of intact JMJD7 (2 μM) after 30 min incubation with DRG1-Ser (20 μM).


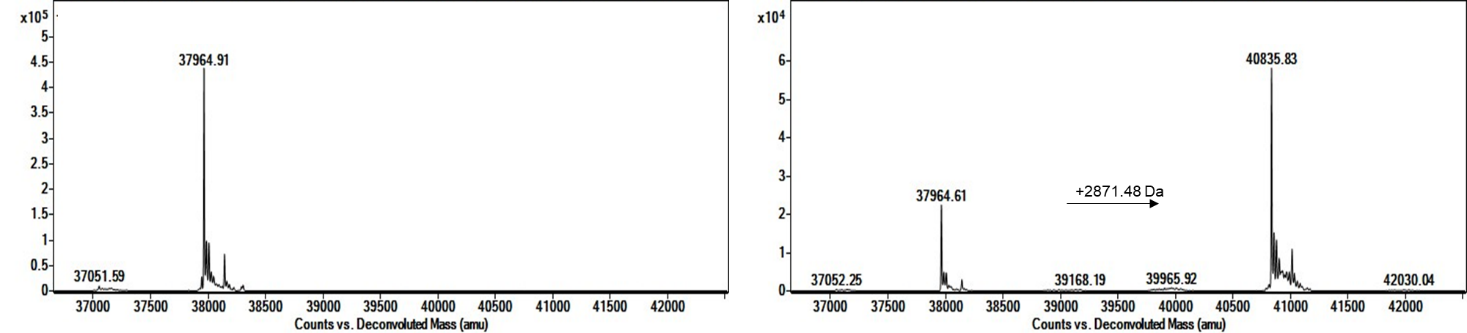


Figure S46 Q-TOF-MS data of intact JMJD7 (2 μM) after 30 min preincubation with DRG1-Lys (200 μM), followed by addition of DRG1-Sec (20 μM) (1 min, left and 15 min, right).
